# Comparative Transcriptomics Highlights New Features of the Iron Starvation Response in the Human Pathogen *Candida glabrata*

**DOI:** 10.3389/fmicb.2018.02689

**Published:** 2018-11-16

**Authors:** Médine Benchouaia, Hugues Ripoche, Mariam Sissoko, Antonin Thiébaut, Jawad Merhej, Thierry Delaveau, Laure Fasseu, Sabrina Benaissa, Geneviève Lorieux, Laurent Jourdren, Stéphane Le Crom, Gaëlle Lelandais, Eduardo Corel, Frédéric Devaux

**Affiliations:** ^1^Sorbonne Université, CNRS, Institut de Biologie Paris-Seine, UMR 7238, Laboratoire de Biologie Computationnelle et Quantitative, Paris, France; ^2^École Normale Supérieure, PSL Research University, CNRS, Inserm U1024, Institut de Biologie de l’École Normale Supérieure, Plateforme Génomique, Paris, France; ^3^Sorbonne Université, CNRS, Institut de Biologie Paris-Seine, UMR 7138, Évolution, Paris, France; ^4^UMR 9198, Institute for Integrative Biology of the Cell, CEA, CNRS, Université Paris-Sud, UPSay, Gif-sur-Yvette, France

**Keywords:** yeast, Aft, ChIP-seq, NO GO decay, evolution

## Abstract

In this work, we used comparative transcriptomics to identify regulatory outliers (ROs) in the human pathogen *Candida glabrata*. ROs are genes that have very different expression patterns compared to their orthologs in other species. From comparative transcriptome analyses of the response of eight yeast species to toxic doses of selenite, a pleiotropic stress inducer, we identified 38 ROs in *C. glabrata*. Using transcriptome analyses of *C. glabrata* response to five different stresses, we pointed out five ROs which were more particularly responsive to iron starvation, a process which is very important for *C. glabrata* virulence. Global chromatin Immunoprecipitation and gene profiling analyses showed that four of these genes are actually new targets of the iron starvation responsive Aft2 transcription factor in *C. glabrata*. Two of them (*HBS1* and *DOM34b*) are required for *C. glabrata* optimal growth in iron limited conditions. In *S. cerevisiae*, the orthologs of these two genes are involved in ribosome rescue by the NO GO decay (NGD) pathway. Hence, our results suggest a specific contribution of NGD co-factors to the *C. glabrata* adaptation to iron starvation.

## Introduction

Candidemia are systemic infections caused by different *Candida* yeast species. They are responsible for high mortality rates (40–50%) in immunocompromised patients, despite the existing treatments (Pfaller and Diekema, 2007). These last 20 years, *Candida glabrata* has become the second leading cause of candidemia, behind the extensively studied *Candida albicans* (Pfaller et al., 2014). Although they have similar names, *C. glabrata* and *C. albicans* are very different species (Brunke and Hube, 2013). *C. glabrata*, in evolutionary terms, is more closely related to the model yeast *Saccharomyces cerevisiae* than to *C. albicans* (Dujon et al., 2004). It actually belongs to the *Nakaseomyces* clade. In contrast to *C. albicans, C. glabrata* is a haploid. It is less susceptible to the azole compounds which are commonly used to treat candidemia and can rapidly develop high-level resistance (Pfaller and Diekema, 2007). Moreover, it has evolved distinct invasive strategies and unique transcriptional responses to stress compared to other pathogenic fungi. For instance, *Candida glabrata* is able to survive and multiply in macrophages by escaping or inhibiting most of the phagolysosome anti-microbial weapons (Kaur et al., 2007; Seider et al., 2011, 2014; Kasper et al., 2015). Identifying the specificities of *C. glabrata* is therefore a key issue to understand its virulence and eventually find efficient treatments.

One obvious way to find *C. glabrata* particularities is comparative genomics (Gabaldon and Carrete, 2015). The comparison of *C. glabrata* and *S. cerevisiae* genomes indicated that *C. glabrata* has lost some genes involved in galactose, phosphate, nitrogen, and sulfur metabolisms (Roetzer et al., 2011). These gene losses resulted in auxotrophy for nicotinic acid, pyridoxine, and thiamine (Dujon et al., 2004; Domergue et al., 2005). These features were hypothesized to be related to the pathogenic nature of *C. glabrata*. However, the sequencing of five more *Nakaseomyces* species, including two species found in human patients and three, non-pathogenic, environmental species, showed that most of these changes were actually shared by both pathogenic and non-pathogenic *Nakaseomyces* species (Gabaldon et al., 2013). This study identified the amplification of the *EPA* genes, which encode for glycosyl-phosphatidylinositol (GPI)-anchored cell wall proteins involved in cell adhesion, stress responses and recognition by the innate immune system (Cormack et al., 1999; De Las Penas et al., 2003, 2015; Domergue et al., 2005; Juarez-Cepeda et al., 2015; Vitenshtein et al., 2016) as the main genomic feature correlating with virulence in this clade (Gabaldon et al., 2013; Gabaldon and Carrete, 2015).

Besides gene gains and losses, phenotypic diversity can also arise from gene regulation divergence (Romero et al., 2012; Roy and Thompson, 2015; Thompson et al., 2015; Johnson, 2017). Hence, numerous cases have been described in which changes in the *cis*- or *trans*- regulatory elements of otherwise conserved genes can lead to the emergence of new functions (Romero et al., 2012; Thompson et al., 2015). For instance, the evolution of pregnancy in mammals was associated with transcriptional network rewiring driven by transposable elements (Lynch et al., 2011, 2015). In yeasts, the loss of an AT rich *cis*-regulatory element in the promoters of oxidative phosphorylation and mitochondrial ribosomal protein genes following a Whole Genome Duplication event (WGD) allowed for the appearance of a respiro-fermentative life style in extent post-WGD species (Ihmels et al., 2005; Habib et al., 2012; Thompson et al., 2013). Comparative transcriptomics (i.e., the comparison of gene expression profiles in different species) has been extensively used in yeasts to identify changes in gene regulation that accompanied the appearance of new physiological properties (Ihmels et al., 2005; Lavoie et al., 2010; Wapinski et al., 2010), to achieve model phylogeny for regulatory evolution (Roy et al., 2013; Thompson et al., 2013) or to predict transcriptional regulatory networks in non-model species (Koch et al., 2017). In the present work, we used comparative transcriptomics to identify regulatory outliers (ROs) in *C. glabrata* and in seven other yeast species. ROs are genes that have very different expression profiles from their orthologs in the other species. To find them, we designed REGULOUT, a program which automatically identifies genes with unique profiles among their group of orthologs (i.e., orthogroups). We applied REGULOUT to comparative transcriptome analyses of the response of eight yeast species to toxic doses of selenite, a pleiotropic stress inducer. From these data, REGULOUT identified 38 ROs in *C. glabrata*. Using transcriptome analyses of the *C. glabrata* response to five different stresses, we pointed out five *C. glabrata* ROs which were more particularly responsive to iron starvation, a process which is very important for *C. glabrata* virulence (Nevitt and Thiele, 2011; Srivastava et al., 2014). Global chromatin Immunoprecipitation (ChIP-seq) and gene profiling analyses showed that these five genes were under the control of the iron starvation responsive transcription factor Aft2 and that four of them were actually *C. glabrata* specific Aft2 targets as compared to *S. cerevisiae*. Phylogenetic analyses of the promoter sequences of these four genes suggest that their control by Aft2 was fixed after the WGD. Interestingly, the amount of Aft motifs in the promoters of those genes was particularly high in the *Nakaseomyces* sub-clade including the three potentially pathogenic species sequenced to date (namely *C. glabrata, Candida bracarensis* and *Candida nivariensis*), as compared with the non-pathogenic *Nakaseomyces* sub-clade or with the *Saccharomyces* genus. Among these four genes, two (*HBS1* and *DOM34b*) were required for optimal growth of *C. glabrata* in iron limited conditions. In *S. cerevisiae*, the orthologs of these two genes are involved in ribosome rescue by the NO GO decay (NGD) pathway. Hence, our results demonstrate the power of comparative functional genomics to identify novel regulatory systems in non-model species and suggest a specific contribution of NGD co-factors to the *C. glabrata* strategy for its adaptation to iron starvation.

## Materials and Methods

### Strains and Growth Conditions

For comparative transcriptomic analyses, we used the following strains: *Saccharomyces cerevisiae* S288C, *Candida glabrata* CBS138, *Lachancea kluyveri* CBS3082, *Lachancea thermotolerans* CBS6340, *Kluyveromyces lactis* CBS2359, *Candida albicans* SC5315, *Debaryomyces hansenii* CBS767, *Yarrowia lipolytica* CLIB122. All strains were grown in rich media at 30°C (YPD: 1% bacto peptone, 1% yeast extract, 2% glucose) on a rotating shaker (150 rpm), except the halophilic yeast *D. hansenii* which was grown in YPD supplemented with 0.5 M NaCl.

For *C. glabrata* mutant strain construction, we used the HTL background (*his3-, trp1-, leu2-*) (Schwarzmuller et al., 2014). The tagging of *DOM34a, DOM34b, HBS1*, and *MAK16b* with TAP tag was performed by PCR and homologous recombination as described in Merhej et al. (2015). The tagging cassette was PCR amplified from the pBS1479 plasmid (Puig et al., 2001). The tagging of *AFT2* with a myc epitope was performed exactly as described (Merhej et al., 2015) using the myc-His cassette from the Longtine’s collection (Longtine et al., 1998). The *AFT2* deleted strain was obtained from the Schwarzmuller et al. (2014) collection. The *MAK16b* deleted strain was obtained by PCR and homologous recombination as described previously (Merhej et al., 2015). The *HBS1, DOM34a*, and *DOM34b* deletion mutants were obtained by a two steps PCR process using large homologous regions as described previously (Schwarzmuller et al., 2014). For all deletions, we used the *TRP1* deletion cassette from the Longtine’s collection (Longtine et al., 1998). For all constructs, the positive clones were selected by growth on CSM-TRP media, except for the *AFT2-MYC* tagged construct which was screened on CSM-HIS media (2% glucose, 0.67% yeast nitrogen base, recommended amounts of CSM-HIS or CSM-TRP from MP Bio). The proper insertion of the cassettes at the targeted genomic loci and the absence of wild type versions of the targeted gene was controlled by PCR. All primers used in this study are available in Supplementary File S1.

### Multispecies Transcriptome Analyses: Basic Experimental Set Up and RNA Extractions

For each species, we first determined the dose of selenite required for a 100% decrease of the growth rate in exponential phase. The results were: 0.5 mM for *C. albicans, D. hansenii, K. lactis*, and *L. kluyveri*, 1 mM for *C. glabrata*, 5 mM for *L. thermotolerans*, 10 mM for *S. cerevisiae* and *Y. lipolytica*. Next, we performed kinetic experiments in which the cells were grown in YPD (or YPD + salt for *D. hansenii* as indicated above) at 30°C until they reach an optical density (OD) at 600 nm of 0.6–0.7. Then, we split the cultures in two: one sub-culture received a mock treatment while the second was treated by the indicated amount of selenite (sodium selenite from sigma, stock solution prepared at 0.2 M in water). This step defines time zero. Every 10 min from time 10 to time 80 min, 20 mL of each culture were collected and flash-frozen in 30 mL of cold (-80°C) absolute ethanol. The cells were centrifuged (5 min, 4,000 rpm), washed with cold (4°C) distilled sterilized water, centrifuged again and cell pellets were stored at -80°C. In parallel to sample collection, the OD of the two cultures was measured every 30 min for 3 h to assess the impact of the selenite treatment on the growth rate. For each species, 6–10 independent kinetic series were generated. Only the four series which were the closest to the 100% decrease of growth rate as compared to the mock treated cultures were used for RNA extraction and microarray analyses. RNAs were extracted using the RNeasy kit (Qiagen) following the protocol provided by the supplier. The quality of the RNA extracts was checked on agarose gels prior to reverse transcription.

### Multispecies Transcriptome Analyses: Microarray Design, Microarray Experiments, Data Analyses

Agilent 8x60k custom microarrays were designed for each species (array express accession numbers: A-MEXP-2402, A-MEXP-2365, A-MTAB-642 to 647). For probes design, we used the Teolenn software version 2.0.1 (Jourdren et al., 2010). The genome sequences and ORFs positions used to create probe sequences were downloaded from the Genolevures website. Only the coding sequences were used for probe design, introns and intergenic regions were not considered. The masked genomes (i.e., without repeated sequences) required for Teolenn were created using RepeatMasker version open-3.2.8 with the “-pa 4 -species fungi -xsmall” arguments. The main features of the design are summarized in Supplementary File S2. There were no probe replicates on the arrays but each ORF was covered by eight different probes in average. For microarray experiments, 1 μg of total RNA was used for fluorescent cDNA synthesis according to the amino-allyl protocol (Merhej et al., 2015). The cDNAs were labeled with Cy3 and Cy5 and hybridization was performed as previously described (Merhej et al., 2015). Four biologically independent experiments were performed for each condition, using dye switch. After overnight hybridization and washing, the slides were scanned using a 2-micron Agilent microarray scanner. The images were analyzed using the feature extraction software (Agilent Technologies) and normalized using global LOESS (Lemoine et al., 2006). The mean of the four biological replicates was calculated. A gene was considered as being induced (repressed) if its mean Log_2_(fold change) value was more than 0.75 (less than -0.75) and if its expression variation was considered as being statistically significant using the LIMMA package with a cut-off *p*-value of 0.01 (Ritchie et al., 2015) for at least two consecutive time points. The complete microarray data are available at Array express database under the accession numbers: E-MTAB-7022, E-MTAB-7023, E-MTAB-7044 to 7047, E-MTAB-7049 and E-MTAB-7053. The processed dataset is available in Supplementary Table T1.

### REGULOUT

REGULOUT is a python program which takes as an input a multispecies expression matrix and the repartition of the genes in orthogroups. There are two parameters which need to be set up by the user: the minimal size of the orthogroups to be used and the Euclidian distance cut-off to be applied. Then REGULOUT calculates, for each orthogroup reaching the defined minimal size, the pairwise distances between the expression profiles of the genes belonging to the orthogroup. In a next step, REGULOUT defines as ROs all the genes which minimal pairwise distance in their orthogroup is equal to or higher than the minimal distance cut-off. As an output, REGULOUT provides a text file with the name of the ROs, their minimal distance in their orthogroup, the ID number and the size of their orthogroup. It also generates automatically PNG files with the expression profiles of the orthogroups in which at least one RO has been identified. REGULOUT can be freely downloaded from www.lcqb.upmc.fr/REGULOUT/, together with a tutorial and the input data sets used in this study. More precisely, the input files used in this article were the expression matrix for all species and the composition of yeast orthogroups taken from the PhylomeDB database (Huerta-Cepas et al., 2014). The distribution of the sizes of the orthogroups is available in Supplementary File S3. The minimal size of the orthogroups to be used was set up to 8 to work mostly with genes which were conserved in all the eight yeast species that were considered. The impact of this filter on the number of expression profiles analyzed by REGULOUT is indicated in Supplementary File S2. The minimal distance cut-off was set up at 3, which corresponded to the 75 percentile of all the possible pairwise distances in the dataset. From the raw output of REGULOUT (440 genes), we filtered out the genes which were defined as ROs based on expression variations that were poorly reproducible (*p*-value > 0.01). Because the Euclidian distance that we used is very sensitive to stochastic variations for genes with a large amplitude in their expression changes, we also removed from the RO list the genes belonging to orthogroups in which all the members had the same directionality in their expression variations (see Supplementary File S4 for an illustration). The filtered REGULOUT output contained 289 ROs which are listed in Supplementary Table T2.

### Multi-Stress Transcriptome Analyses in *C. glabrata*

All cultures were conducted in a rotating shaker at 30°C in YPD (glucose 2%, yeast extract 1%, bacto peptone 1%). Stress conditions used were: 1 mM for sodium selenite, 1 M for NaCl, 2 mM for cadmium chloride, 5 mM for iron sulfate or 0.5 mM for bathophenanthroline disulfonate (BPS). Stressed and mock-treated cells were collected as described above, 20 min and 40 min after treatment. RNA extractions, microarray hybridization and data normalization were performed as described above. For the *aft2Δ* versus wild type comparisons, the stress concentrations were the same as above but the cells were collected only 30 min after treatment. The complete microarray data are available at Array express database under the accession numbers: E-MTAB-7023, E-MTAB-7042, and E-MTAB-7043.

### Chromatin Immunoprecipitation and High-Throughput Sequencing (ChIP-Seq)

For Aft2 ChIP experiments, myc-tagged strains were grown in YPD until exponential phase (OD = 0.8), they were then treated by 0.5 mM of BPS for 60 min. Cross-linking of the cells and ChIP were performed as described previously (Merhej et al., 2016). The parental HTL (untagged strain) was grown and processed the same way to provide the mock-IP samples. Sequencing of the IPs, Input DNAs and mock IPs samples and primary data analyses (quality controls and mapping of the reads) were performed as described previously (Merhej et al., 2016). Peak calling was performed with the bpeaks software, using both the Input DNA and the mock IP as references (Merhej et al., 2014). For peak calling using the Input DNA as reference, the bpeaks parameters were T1 = 1, T2 = 6, T3 = 1, T4 = 0.7. For peak calling using the Mock IP as reference, the bpeaks parameters were T1 = 1, T2 = 6, T3 = 1, and T4 = 0. Only the peaks that were found in both analyses were kept for further processing. These peaks were then manually checked on a genome browser (Thorvaldsdottir et al., 2013) to discard artifactual peaks (e.g., peaks centered on a tRNA locus, peaks perfectly overlapping a highly expressed ORF) which would have escaped the bpeaks filter. Peaks located outside of a promoter region (i.e., between convergent ORF or inside ORFs) were also discarded from the final list presented in Supplementary Table T3. For DNA motif prediction, DNA sequences of ChIP peaks were retrieved from their genomic locations (BED file) using the “getfasta” function from the BEDTOOLS suite (Quinlan and Hall, 2010). These genomic sequences were used as inputs for the peak-motif tool to search for regulatory motifs (Thomas-Chollier et al., 2012). An additional filtering step was added to the standard peak motif procedure to discard low complexity motifs (e.g., CCCCCCC). The list of Aft targets from *S. cerevisiae* was obtained from Yeastract (Teixeira et al., 2018) and completed by the “regulation” pages of the SGD ^1^, taking all the documented regulatory interactions using DNA binding or expression evidences and keeping only the targets which were found in relevant growth conditions (i.e., metal stress conditions). The raw ChIP-seq data can be downloaded from the GEO database with the accession number GSE116077.

### Bioinformatics Analyses: Gene Ontology, Hierarchical Clustering, Promoter Sequence Analyses

Gene ontology (GO) analyses were performed using the GO term finder tool at the Candida Genome Database ^2^ for *C. albicans, C. glabrata*, and *D. hansenii* (with the names of the *C. albicans* orthologs), at the Saccharomyces Genome Database for *S. cerevisiae*, the two *Lachancea* species, *K. lactis* and *Y. lipolytica* (using the names of the *S. cerevisiae* orthologs). The hierarchical clustering of Figure 2 was performed using Mev with Euclidian distance, optimization of gene leaf order and average linkage. For phylogenetic analyses of the promoter sequences, we downloaded the promoter sequences (i.e., 500 base pairs upstream of the ATG) from the Genome Resources for Yeast Chromosomes database (GRYC^3^), except for the *Saccharomyces* sequences which were taken from the www.saccharomycessensustricto.org website (Scannell et al., 2011) and for the *Kazachstania Africana* sequences which were downloaded from the NCBI website. The sequences used can be found in Supplementary Files S5–S8.

### Western Blot Analyses

The cells were grown in YPD at 30°C until they reach an OD at 600 nm of 0.6–0.7. Then, we split the cultures in two: one sub-culture received a mock treatment while the second was treated by 0.5 mM BPS. This step defines time zero. At 30, 60, and 90 min, 10 mL of each culture were collected and centrifuged (5 min, 4,000 rpm), washed with cold (4°C) distilled sterilized water, centrifuged again and cell pellets were stored at -80°C. Proteins were separated on 10% SDS-polyacrylamide gel electrophoresis (SDS-PAGE). Proteins were then transferred to Whatman^®^ Protan^®^ BA83 nitrocellulose membrane (GE Healthcare). Immunoblotting of the protein A-tagged proteins was performed with a rabbit IgG-HRP polyclonal antibody (PAP; code Z0113; Dako), which has a high affinity for Protein A. The membrane was stripped by boiling 30 min in 62.5 mM Tris-HCl pH 6.8, SDS 2% and 4 mM DTT, followed by 10 washes in PBS-Tween (0.1%). Then, immunoblotting of the ribosomal protein Rpl3, used as a loading and transfer control, was performed using 1:10000 rabbit IgG Anti-Rpl3 [gift from M. Garcia: refer to (Delaveau et al., 2016)] and 1:15000 anti-rabbit IgG-HRP (Promega) as primary and secondary antibodies, respectively. Detection of the signals was performed using G:BOX Chemi XT4 (Syngene) following incubation with UptiLightTM HRP blot chemiluminescent ECL substrate (Interchim).

### Growth Assays

Growth assays were performed in 96 well plates using a TECAN Sunrise machine. Exponential phase growing cultures of the strains to be tested were diluted to OD = 0.1 in a 50 mL sterile Falcon tube. The 96 well plate was filled with 90 μL of the cultures. Then, 10 μL of BPS 0.5 mM stock solution (iron starvation conditions, final concentration 0.05 mM) or 10 μL of sterile water (mock treatment) were added to each well to reach a final culture volume of 100 μL. Cell growth at 30°C was followed for 24 h. The slope of the linear part of the log(OD) = f(t) curve was extracted and used as the growth rate of each culture in exponential phase. Two technical replicates were made in each plate.

## Results

### Identifying Regulatory Outliers From Comparative Transcriptomics Data

The starting point of this study consisted in analyzing and comparing the response of eight yeast species (*S. cerevisiae, C. glabrata, L. kluyveri, L. thermotolerans, K. lactis, C. albicans, D. hansenii*, and *Y. lipolytica*) to the stress caused by detrimental doses of selenite. These eight species were chosen because they span the whole Hemiascomycetes tree and because of their high-quality genome annotation at the time when we started the study (Genolevures et al., 2009). Selenite was chosen because it was shown to induce both iron starvation and oxidative stress responses in *S. cerevisiae* (Salin et al., 2008; Herrero and Wellinger, 2015), two stress conditions that *C. glabrata* is facing when invading the human body and being internalized by the cells of the innate immune system (Nevitt and Thiele, 2011; Brunke and Hube, 2013). A key challenge in comparative transcriptomics is to set up the growth conditions to make the physiological state of the different species as similar as possible and to minimize irrelevant differences in gene expression (Kuo et al., 2010; Thompson et al., 2013). To do so, we adjusted the selenite doses used for each species to obtain the same 100% increase in the generation time. Then, RNA samples were collected at eight time points after selenite exposure and compared to the RNAs extracted from a control culture mock-treated for the same duration using microarrays. Four replicates were performed for each species and each time point. Supplementary Table T1 provides the average log_2_ of the expression ratios between treated and untreated cells for each measured gene in each species for the eight time points, together with a statistical assessment of the significance of the gene expression variations.

**FIGURE 1:**
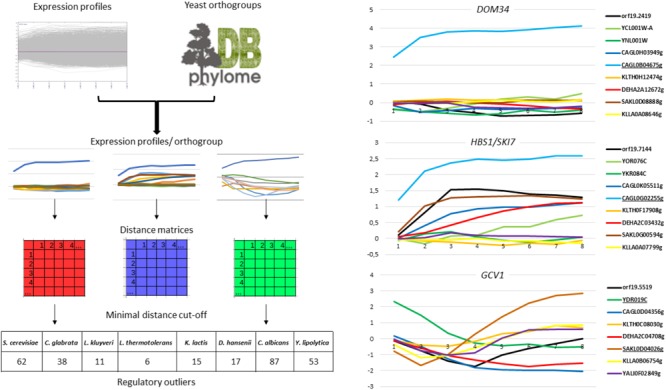
Rationale and outputs of REGULOUT. **(Left)** This scheme summarizes the rationale of REGULOUT. The table at the bottom indicates the number of regulatory outliers identified for each species with the parameters indicated in the material and methods. **(Right)** Expression profiles [Log_2_(selenite/mock treatment expression ratio)] as a function of time (1 unit = 10 min) for three orthogroup (*DOM34, HBS1/SKI7*, and *GCV1*). The names of the genes in the orthogroup are indicated at the right of each graph. The names of the regulatory outliers are underlined. The colors are: blue for *C. glabrata*, green for *S. cerevisiae*, black for *C. albicans*, orange for *L. thermotolerans*, red for *D. hansenii*, brown for *L. kluyveri*, yellow for *K. lactis*. The phylome DB logo was taken from the phylome DB database website (Huerta-Cepas et al., 2014).

As *a posteriori* validation of our approach, we examined the expression patterns of genes from the general Environmental Stress Response (ESR), which were shown to be highly conserved among the species we studied (Gasch et al., 2000, 2004; Gasch, 2007; Roy et al., 2013; Thompson et al., 2013). We observed that the induction of genes encoding proteasomal subunits and the repression of genes from the Ribosome Biogenesis (Ribi) regulon were consistent between the different species (Supplementary File S9). This was a good indication that the eight species were encountering physiologically similar stress conditions. To identify ROs from these 44,723 expression profiles, we designed a program called REGULOUT (Figure 1, left panel). REGULOUT takes as input files the expression profiles obtained in the different species and a table describing the orthology relationships between genes (i.e., the names of the genes composing each orthogroup). For each orthogroup taken independently, it calculates all the pairwise distances between the expression profiles of all the genes belonging to the orthogroup. Then, it looks for genes which minimal distance value in the orthogroup is higher than a distance cut-off set up by the user. Those genes with special expression profiles are the so-called “ROs.” The key parameters for REGULOUT are the minimal distance that the user accepts to define a RO and the minimal number of genes for an orthogroup to be considered in the analysis. We applied REGULOUT to our data with parameters corresponding to genes conserved in most of our yeast species (i.e., minimal orthogroup size = 8) and to a minimal distance cut-off equal to the 75 percentile of all the calculated pairwise distances (i.e., *d* = 3). With this criterion, we identified a total of 289 ROs in the eight species, ranging from 6 in *L. thermotolerans* up to 87 in *C. albicans* (Figure 1 and Supplementary Table T2). Careful examination of the corresponding expression profiles led us to distinguish three different situations: directional ROs, quantitative ROs and timing-related ROs. Directional ROs are unique in the directionality of their regulation (i.e., up-regulation, down-regulation or unchanged expression). This is exemplified by the *DOM34* orthogroup in Figure 1 (right panel). In this example, the expression of one *C. glabrata* member (*CAGL0B04675g*) is strongly induced by selenite while its paralog in *C. glabrata* and its orthologs in the seven other species show unchanged or reduced expression. The quantitative ROs have the same directionality of regulation than other members of the orthogroup, but with much larger amplitude. This is exemplified by the *HBS1/SKI7* orthogroup in the right panel of Figure 1. *HBS1* is induced by selenite in *C. albicans, D. hansenii, L. kluyveri*, and *C. glabrata*, but the induction of the *C. glabrata* gene *CAGL0G02255g* is sufficiently higher than its orthologs to be considered as a RO by REGULOUT. The timing-related ROs have the same directionality and range of regulation than some of their orthologs, but with a different timing. This is exemplified by the *GCV1* orthogroup in Figure 1 (right panel). *GCV1* is induced in several species following selenite exposure but the pattern of early and transient induction is unique to the *S. cerevisiae* member of this group (*YDR019c*).

Gene ontology analyses indicated that only a few GO terms were enriched in the RO lists of the different species (Supplementary File S10). These include genes of the arginine biosynthesis pathway (*ARG3, ARG4, ARG5,6, ARG8, CPA1, CPA2*), which were much more strongly induced by selenite in *C. albicans* than in any other species, and orthologs for the two main actors of a ribosome disassembly pathway named NGD (*DOM34* and *HBS1*), which were specifically induced in *C. glabrata*. Also, several amino-acid metabolism genes (*ARG3, HIS1, HIS4, HIS7, CPA1, LYS12, MET6, TRP3*) had special expression patterns in *C. glabrata*. This was already noticed in a previous comparative transcriptomics study analyzing the heat shock stress response in eight yeast species (Roy et al., 2013).

### Five ROs in *C. glabrata* Are Induced in Iron Starvation Conditions

**FIGURE 2:**
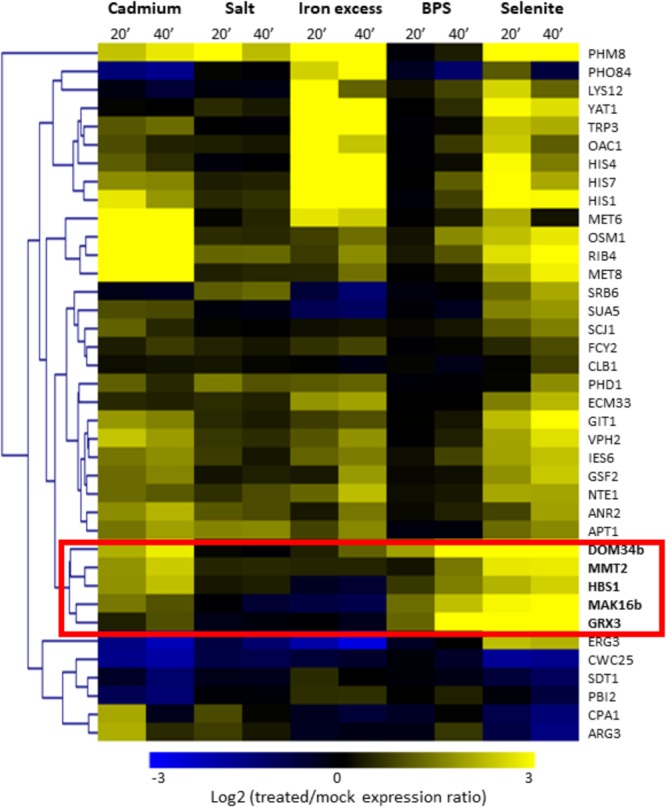
Hierarchical clustering of the 38 *C. glabrata* ROs based on their expression profiles in response to different stress conditions. Expression profiles [Log_2_(stressed cells/mock treatment expression ratio)] are represented by a color code (scale at the bottom). Two duration times of treatment were used (20 min and 40 min). The names of the genes are based on the names of their closest homologs in *S. cerevisiae*. A group of genes which is more particularly induced in iron limited conditions (BPS and selenite) has been highlighted by a red box.

As mentioned above, selenite triggers several stress responses (iron starvation, oxidative stress response, DNA damage…) (Pinson et al., 2000; Salin et al., 2008; Perez-Sampietro et al., 2016) and therefore the ROs identified from the selenite comparative transcriptomics data may actually respond to different physiological signals and pathways. We were more particularly interested in identifying ROs in *C. glabrata* which would be linked to the iron starvation response. With this goal in mind, we focused on the 38 *C. glabrata* ROs and took advantage of transcriptome data analyzing the response of *C. glabrata* to five different stress conditions (osmotic stress, iron excess, cadmium, BPS or selenite treatments), which were obtained in the frame of another project (unpublished data). For the present work, we only extracted the data for the *C. glabrata* ROs (Supplementary Table T4). We then clustered them according to their expression profiles in these five stress conditions (Figure 2). Doing so, we could point out a group of five ROs which showed early and strong induction in response to the two iron starvation conditions (namely BPS and selenite treatments) and unchanged or slightly increased expression in response to osmotic and iron excess stresses. This group included the *C. glabrata* orthologs of the *S. cerevisiae* genes encoding the Grx3 glutaredoxin (*CAGL0L11990g*), the Mmt2 mitochondrial iron transporter (*CAGL0E06006g*), the Mak16 ribosome biogenesis factor encoding (*CAGL0F00715g*) and the Dom34 and Hbs1 translation surveillance factors (*CAGL0B04675g* and *CAGL0G02255g*). For the sake of simplicity, we will call the *C. glabrata* genes by the name of their *S. cerevisiae* orthologs in the rest of the article. For *DOM34* and *MAK16*, two paralogs exist in *C. glabrata*. In these cases, the BPS and selenite inducible versions will be called *MAK16b* and *DOM34b* and the versions with unchanged or repressed expression patterns will be called *MAK16a* (*CAGL0G06248g*) and *DOM34a* (*CAGL0H03949g*). Among these five ROs, three were directional ones (*GRX3, DOM34b* and *MAK16b*), meaning that they were induced by selenite only in *C. glabrata*, and two were quantitative ROs (*HBS1* and *MMT2*), meaning that they were also induced in other species, but to a lesser extent than in *C. glabrata* (Supplementary File S11).

### *GRX3, DOM34b, HBS1*, and *MAK16b* Are New Targets of the Aft2 Transcription Factor in *C. glabrata*

Interestingly enough, these five genes were also induced by cadmium in *C. glabrata* (Figure 2). In the model yeast *S. cerevisiae*, BPS and selenite trigger an iron starvation transcriptional response which is controlled by the two paralogous transcription factors Aft1 and Aft2 (Blaiseau et al., 2001; Rutherford et al., 2001, 2003; Courel et al., 2005; Salin et al., 2008; Perez-Sampietro and Herrero, 2014; Perez-Sampietro et al., 2016). However, only the Aft2 regulon is reproducibly induced by cadmium, while most of the Aft1 regulon remains unchanged or is even repressed (Fauchon et al., 2002; Caetano et al., 2015). The expression profiles of the five ROs identified in Figure 2 hence suggested that their particular stress regulation pattern in *C. glabrata* could be due to Aft2.

**FIGURE 3:**
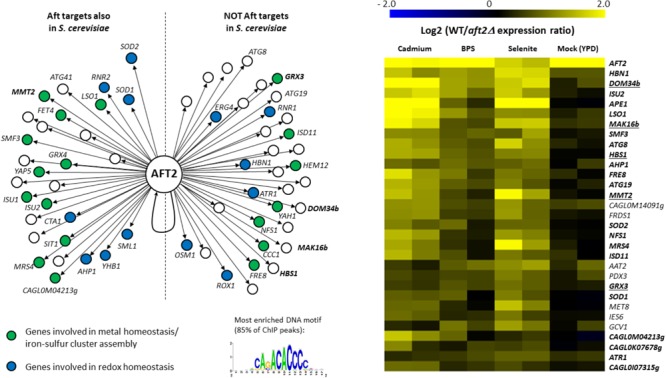
The Aft2 regulon in *C. glabrata*. **(Left)** The 63 Aft2 target promoters, as defined by ChIP-seq (Supplementary Table T3), were separated in two groups: target promoters which ortholog is also an Aft target in *S. cerevisiae* and target promoters which ortholog has not been identified as an Aft target in *S. cerevisiae*. Only the names of the genes which are discussed in the main text are indicated. The names of the five ROs of interest are in bold. The targets are colored according to the two main GO terms enriched among Aft2 targets: blue = redox homeostasis, green = iron metabolism. The most enriched DNA motif among all ChIP-peaks is indicated at the bottom right. **(Right)** Transcriptomic comparison of gene expression between the wild type and the *aft2Δ* mutant in four growth conditions. The 30 most affected genes are represented here. The names of genes which were also targeted by Aft2 in our ChIP-seq experiment are in bold. The names of the five ROs of interest have been underlined. The results of two biologically independent replicates are indicated for each growth condition. The complete results are available in Supplementary Table T5.

The Aft2 regulon was not previously deciphered in *C. glabrata*. Then, to test our hypothesis, we conducted genome-wide chromatin immunoprecipitation (ChIP-seq) on a Aft2 myc-tagged strain in iron starvation conditions induced by BPS treatment. ChIP-seq analyses identified 63 promoters bound by Aft2, corresponding to 88 potential gene targets (Supplementary Table T3 and Figure 3, left panel). Sequence analyses of the ChIP peaks identified the ACACCC motif as being the most enriched in the Aft2 bound locus, being present in 80% of the target promoters (Figure 3, left panel). This motif is identical to the consensus previously identified for Aft2 in *S. cerevisiae* (Courel et al., 2005; Conde e Silva et al., 2009). GO analyses revealed an enrichment in genes involved in iron homeostasis (*p*-value = 5.15e-08) and oxidative stress response (*p*-value = 0.00276) (Figure 3, left panel). More specifically, Aft2 targeted genes belonging to the iron homeostasis category were involved in the reductive iron uptake pathway (*FRE8, FET4*), the siderophore iron uptake pathway (*SIT1*), the intracellular iron transport (*MRS4, SMF3, CCC1, MMT2*) and the iron–sulfur cluster biogenesis and assembly pathway (*ISU1, ISU2, ISD11, NFS1, YAH1*). In connection with redox homeostasis, Aft2 bound the promoters of the genes encoding the reductases *HBN1, OSM1, YHB1, RNR1, RNR2*, and *ERG4*, the superoxide dismutases *SOD1* and *SOD2*, the catalase *CTA1* and the peroxiredoxin AHP1. Interestingly, Aft2 also targets genes involved in autophagy (*ATG8, ATG19*, and *ATG41*), which may be consistent with the recent finding that mitophagy is triggered by iron starvation in *C. glabrata* (Nagi et al., 2016).

Then we compared the set of targets identified in *C. glabrata* with those of Aft2 and Aft1 in *S. cerevisiae* (Figure 3, left panel). Half of the *C. glabrata* Aft2 targets were also targets of Aft1 and/or Aft2 in *S. cerevisiae* (Figure 3, left panel). Among these conserved targets is *MMT2*, which was consistent with our observation that *MMT1* and *MMT2* are also induced by selenite in *S. cerevisiae* (Supplementary File S11). Reciprocally, 50% of the Aft2 targets were specific to *C. glabrata* (“specific” meaning here as compared to *S. cerevisiae*). Among these *C. glabrata* “specific” Aft2 targets were *GRX3, DOM34b, HBS1*, and *MAK16b*.

To measure the impact of Aft2 on the expression of its potential targets, we performed transcriptome analyses comparing the *aft2Δ* and wild type strains grown either in optimal conditions (YPD), iron starvation caused by BPS or by selenite, and cadmium exposure. Twenty-four of the 63 promoters bound by Aft2 (38%) were associated to a gene which showed a significant decrease of expression in the *aft2Δ* cells in at least one growth condition. Reciprocally, among the 30 genes which were the most reproducibly affected by *AFT2* deletion, 23 (77%) were directly targeted by Aft2 according to our ChIP-seq results (Figure 3, right panel). Among the seven genes which showed changed expression but no binding in ChIP-seq, only one had a potential connection with iron homeostasis (*MET8*, encoding a ferrochelatase) but remarkably six of them (the exception being *GCV1*) had an Aft2 binding motif in the 500 bp upstream of their ATG (data not shown). These differences in expression were observed only in stress conditions and not in optimal growth conditions (except for *AFT2* itself, which was constitutively deleted in the mutant strain). The four new targets of interest (*GRX3, DOM34b, HBS1*, and *MAK16b*) showed decreased expression in the *aft2Δ* strain in all three stress conditions, but with different ranges (Figure 3, right panel). Hence, our results demonstrate that the increase of expression of *GRX3, DOM34b, HBS1*, and *MAK16b* in *C. glabrata* is controlled by Aft2.

### *DOM34b* and *HBS1* Are Required for Optimal Growth in Iron Starvation Conditions

This regulation of *MAK16b, DOM34b*, and *HBS1* by Aft2 in *C. glabrata* was particularly intriguing because none of the processes they are contributing to in the model yeast *S. cerevisiae*, namely ribosome biogenesis for Mak16, Dom34 and Hbs1 and ribosome rescue pathway for Dom34 and Hbs1, were directly connected to the transcriptional regulation of iron homeostasis.

**FIGURE 4:**
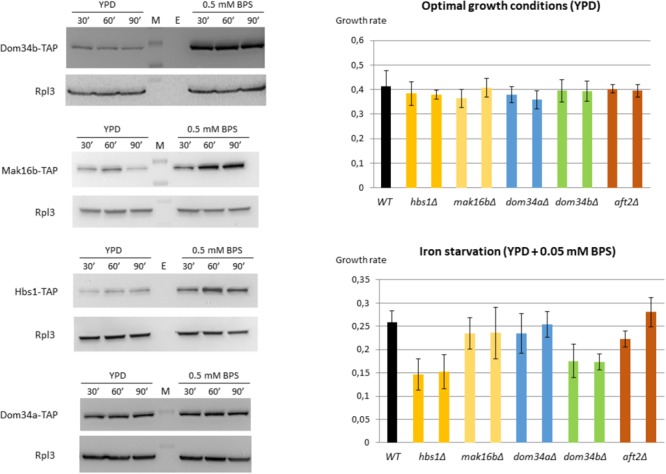
Protein expression levels and fitness analyses in iron starvation conditions. **(Left)** Western blot analyses of the Dom34b-TAP, Hbs1-TAP, Mak16b-TAP, and Dom34a-TAP strains grown in YPD or in YPD + 0.5 mM BPS. Cells were collected 30, 60, and 90 min after the start of BPS or mock treatments. The signal obtained for the Rpl3 ribosomal protein was used as a loading control. Full gel pictures are available in Supplementary File S12. A quantification of the Western blot signals from two biologically independent replicates is available in Supplementary File S13. **(Right)** Growth rates in exponential phase of different strains in YPD (upper) or in YPD + 0.05 mM BPS (lower). Two independent clones were tested for each mutant strains. The values are the average of six measurements: three independent biological replicates with two technical replicates for each of them.

Therefore, we assessed the physiological impact of these new regulatory interactions. First, we wanted to know if the increase that we observed at the mRNA level for *DOM34b, MAK16b*, and *HBS1* in iron starvation conditions was translated to the protein level. We constructed *C. glabrata* strains with genomic TAP-tagged versions of *DOM34b, MAK16b*, and *HBS1*. A strain tagged for *DOM34a* was also constructed as a control for a gene which is not induced by stress at the mRNA level. We performed Western blot analyses on these four strains grown in presence or absence of BPS. We observed a clear and fast induction of Dom34b, Mak16b, and Hbs1 in response to stress, which was perfectly mimicking what was observed at the mRNA level (Figure 4, left panel and Supplementary Files S12, S13). In contrast, Dom34a displayed unchanged expression levels, as expected.

Second, we assessed the importance of *MAK16b, DOM34b*, and *HBS1* in the adaptation to iron starved conditions. We constructed strains deleted for these three genes. We included in our phenotypic analyses strains deleted for *DOM34a* and for *AFT2*. The growth rates of these strains in exponential phase were measured in presence or absence of 0.05 mM BPS and compared to the growth rate of the isogenic wild type strain (Figure 4, right panel). Two independent clones were tested for each strain. All mutant strains had similar growth rates compared to the wild type in standard growth conditions (Figure 4, upper right panel). In presence of BPS, the *dom34aΔ, mak16bΔ* and *aft2Δ* strains did not show any growth defect (Figure 4, bottom right panel). In contrast, the *dom34bΔ* and *hbs1Δ* mutants had a decreased fitness in iron starvation conditions.

### The Regulation of *DOM34b* and *HBS1* by Iron Starvation Probably Arose From the Whole Genome Duplication (WGD)

**FIGURE 5:**
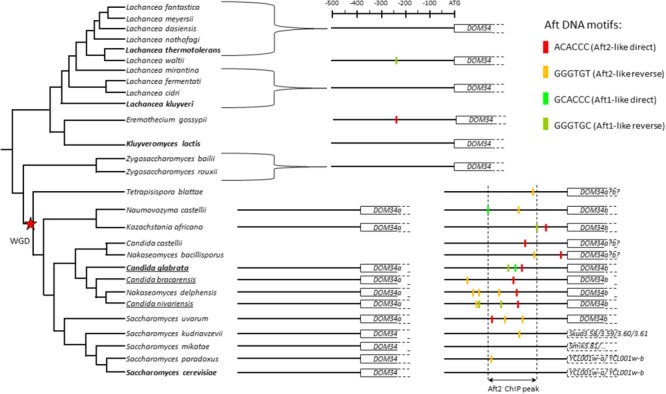
Analyses of the promoters of the *DOM34* orthogroup in 28 *Saccharomycetaceae* species. The tree is a schematic representation of the phylogeny of the species inspired from Vakirlis et al. (2016) for the *Lachancea* species and from Shen et al. (2016) for all the other species. The Whole Genome Duplication (WGD) is indicated by a red star. The positions of the Aft sites in the promoters of the genes are indicated by colored boxes (color code is on the upper right part). The boundaries of the Aft2 ChIP peak in the *C. glabrata DOM34b* gene are indicated by dashed vertical lines. The ORFs in the *DOM34b* lineage of the *Saccharomyces* genus (lower right part of the tree) are interrupted ORFs probably corresponding to pseudogenes. The names of the three pathogenic *Nakaseomyces* species are underlined. The sequences used for this analysis can be found in Supplementary File S5.

As mentioned above, in post-WGD species such as *S. cerevisiae* and *C. glabrata*, there are two Aft transcription factors: Aft2 having a preference for the ACACCC motif and Aft1 being more likely associated to TGCACCC (Courel et al., 2005; Conde e Silva et al., 2009; Goncalves et al., 2014; Srivastava et al., 2015; Gerwien et al., 2016; this work). Besides post-WGD species, the role of the Aft transcription factors in the control of the iron regulon is apparently conserved in the whole *Saccharomycetaceae* family, together with their DNA binding preference for PuGACCC motifs (Conde e Silva et al., 2009; Goncalves et al., 2014). To assess the evolution of the regulation of *DOM34b* and *HBS1* by Aft2 and iron starvation that we characterized in *C. glabrata*, we analyzed the promoter sequences of the orthologs and ohnologs of these two genes in several post- and pre-WGD yeast species spanning the S*accharomycetaceae* tree, looking for ACACCC (Aft2-like) and GCACCC (Aft1-like) motifs on both DNA strands (Figure 5 and Supplementary Files S5, S6, S14).

In post-WGD species, the evolution of the *DOM34* orthogroup is complex and different situations were observed. In some species (e.g., *Candida glabrata* and its three most related *Nakaseomyces* species, but also *Naumovozyma castellii, Kazachstania Africana*, and *Saccharomyces uvarum*) the two ohnologs *DOM34a* and *DOM34b* were retained. In others (e.g., *Tetrapisispora blattae, Candida castellii, Nakaseomyces bacillisporus*) only one copy remained and reciprocal BLAST or synteny analyses could not indicate without ambiguity if it corresponded to the *DOM34a* or *DOM34b* paralog in the other species. Finally, in most *Saccharomyces* species, one of the two ohnologs was split in two or more ORFs by non-coding insertions (e.g., *YCL001w-a* and *YCL001w-b* in *S. cerevisiae*) and is probably on its way to pseudogenization. Promoter analyses indicated a clear enrichment for Aft motifs in the *DOM34b* lineage of the post-WGD species compared to the *DOM34a* lineage or to the *DOM34* orthologs in the pre-WGD species (Figure 5). However, this enrichment is heterogeneous. It is particularly obvious in *C. glabrata* and its close relatives *C. bracarensis, N. delphensis*, and *C. nivariensis*, in which the position of one of the Aft2-like motif is particularly well-conserved while the rest of the promoter sequence largely diverged (Supplementary File S15). In contrast, there is a loss of the Aft motif enrichment in the *Saccharomyces* species, which seems to be correlated to the pseudogenization of one of the two ohnologs.

A similar pattern was observed for *HBS1*: there is a clear enrichment for Aft motif in the *HBS1* lineage of post-WGD species compared with its *SKI7* ohnolog or with the *SKI7/HBS1* orthologs in pre-WGD species (Supplementary File S14). Again, this enrichment in post-WGD species was heterogeneous with, for instance, a clear conservation in the *C. glabrata* sub-clade of the *Nakaseomyces* genus but no Aft motifs in promoters of the *HBS1* versions in the two other *Nakaseomyces* species *C. castellii* and *N. bacillisporus*.

This phylogenetic analysis strongly suggests that the Aft motifs were fixed in the promoters of *DOM34b* and *HBS1* after the whole genome duplication, in a process equivalent to a neo-functionalization event. Then their evolution varied from strong conservation in some species to disappearance in others, possibly reflecting different selection pressures exerted on this new regulation. Yet, it is very important to remember that the presence of an Aft motif does not imply that the motif is active and that the corresponding gene is under Aft regulation. Still, the high conservation of the position of Aft motifs in the promoter of *DOM34b* in *C. glabrata* and its three most related species, while the rest of the promoter diverged, strongly suggests that this motif is under positive selection in these species and then that the regulation that we characterized here for *C. glabrata* is also active in these three species (Supplementary File S15).

## Discussion

In this study, we designed a program, called REGULOUT, for the identification of conserved genes with divergent expression profiles from comparative transcriptomics datasets. The inputs of REGULOUT are multispecies gene expression profiles on one hand and the composition of orthogroups on the other. Hence, REGULOUT can be applied to any group of species for which this information is available. The output of REGULOUT is strongly influenced by the distance cut-off chosen and the type of distance used but also by the way the orthogroups were defined and by the stress conditions which were tested. Hence, the label of “ROs” that we introduced in this article is more a technical concept than a straightforward biological feature and REGULOUT should be used as a fast way to sort out lists of potentially interesting cases and not as a tool for gene annotation. We applied REGULOUT to transcriptomics data obtained from eight yeast species responding to the stress caused by detrimental doses of selenite. To assess the power of REGULOUT in highlighting new, biologically meaningful, regulations, we investigated further the 38 ROs identified in the human pathogen *C. glabrata* and we focused on those which were more particularly induced by stress conditions associated to iron starvation.

Iron acquisition is a critical challenge for most microorganisms and a key virulence factor for many fungal pathogens (reviewed in Bairwa et al., 2017; Gerwien et al., 2018). Iron acquisition genes are required for the survival of *C. glabrata* in the macrophages, its adhesion to epithelial cells and for its virulence in animal models (Nevitt and Thiele, 2011; Seider et al., 2014; Srivastava et al., 2014; Brunke et al., 2015). In *S. cerevisiae*, the iron starvation transcriptional response is controlled by the two paralogous transcription factors Aft1 and Aft2. Aft1 activates the expression of genes involved in iron uptake at the plasma membrane (e.g., *FET3, FTR1, ATX1, FRE1-3, SIT1*, …) and genes involved in the cytoplasmic adaptation to low iron conditions, especially the *CTH1* and *CTH2* genes encoding RNA binding proteins involved in the selective degradation of mRNAs encoding iron consuming proteins (Puig et al., 2005, 2008). Aft2 shares some targets with Aft1 but also specifically activates the expression of genes encoding, for instance, the vacuolar iron transporter Smf3 and the mitochondrial iron transporter Mrs4 (Rutherford et al., 2003; Courel et al., 2005; Martinez-Pastor et al., 2017).

Previous studies have shown that many aspects of the iron homeostasis regulation in *C. glabrata* resemble what had been shown in *S. cerevisiae*. Like *S. cerevisiae, C. glabrata* strongly depends on the reductive pathway and siderophore uptake for extracellular iron acquisition (Nevitt and Thiele, 2011; Srivastava et al., 2014; Gerwien et al., 2016). Low affinity iron transport mediated by the Fet4 protein also exists in *C. glabrata* (Srivastava et al., 2014; Gerwien et al., 2016). We showed here that *FET4* is targeted by Aft2 but the sole deletion of *AFT2* had no impact on its induction by BPS, selenite or cadmium (Supplementary Tables T3, T5). The key genes of the iron regulon in *S. cerevisiae* are also induced by iron starvation in *C. glabrata* (Srivastava et al., 2015; Gerwien et al., 2016; Nagi et al., 2016). *C. glabrata* Aft1 plays a major role in the up-regulation of membrane iron uptake and is itself induced by iron starvation (Srivastava et al., 2015; Gerwien et al., 2016). Moreover, the enrichment of Cth2 motif in the 3′UTR of genes from the *C. glabrata* iron regulon suggests that the post-transcriptional negative regulation of iron consuming genes is also active in this species (Gerwien et al., 2016). In this work, we showed that the role of Aft2 in the control of intracellular iron trafficking and homeostasis is globally conserved. Especially, the two main specific targets of Aft2 in *S. cerevisiae, MRS4* and *SMF3* (Courel et al., 2005), were also regulated by this factor in *C. glabrata* (Figure 3). As in *S. cerevisiae* (Blaiseau et al., 2001; Rutherford et al., 2001), the *aft2Δ* mutant exhibited no particular growth defect in response to stress in *C. glabrata* (Srivastava et al., 2014; Gerwien et al., 2016; Figure 4 of this work).

However, several important differences in iron homeostasis control have also been observed between *C. glabrata* and other model yeast species. For instance, the deletion of the *C. glabrata* ferric reductase encoding genes *FRE8* and *FRE6* does not seem to impact the *C. glabrata* extracellular iron uptake or extracellular ferric reduction activities, in contrast to what has been shown in *S. cerevisiae* and *C. albicans* (Gerwien et al., 2017). Still, these two genes are induced by iron starvation (Nagi et al., 2016; Gerwien et al., 2017) and regulated by Aft2 (this work) and Aft1 (Gerwien et al., 2017), respectively. Moreover, iron starvation was shown to induce the expression of the autophagy genes *ATG32, ATG11*, and *ATG8* (Srivastava et al., 2015; Nagi et al., 2016) and to trigger mitophagy in *C. glabrata* but not in *S. cerevisiae* (Nagi et al., 2016; reviewed in Fukuda and Kanki, 2018). We showed here that the iron starvation induction of *ATG8* is under the control of Aft2 (Figure 3). Of note, *ATG32* and *ATG11* are required for *C. glabrata* dissemination in mice and survival in macrophages, respectively (Roetzer et al., 2010; Nagi et al., 2016).

REGULOUT pointed out *GRX3, MAK16b, DOM34b*, and *HBS1* as being particularly responsive to selenite exposure in *C. glabrata*. Further analyses showed that in *C. glabrata* these four genes were also sensitive to BPS and cadmium treatment, but not to iron excess or osmotic stress (Figure 2). We investigated the regulatory mechanisms underlying these expression patterns and found that the stress response of these four genes is under the control of Aft2 in *C. glabrata* (Figure 3). Our analyses of the evolution of the promoter sequences of the *DOM34* and *HBS1* orthogroups in 14 post-WGD and 14 pre-WGD yeast species revealed enrichment for Aft-like DNA binding motifs in the *DOM34b* and *HBS1* orthologs but neither in the pre-WGD orthologs nor in the *DOM34a* and *SKI7* ohnologs (Figure 5). This strongly suggests that this regulation appeared after the whole genome duplication and was subsequently lost in *Saccharomyces cerevisiae*. This is consistent with previous, more global, observations that duplicated genes are often differentially expressed and evolve divergent regulatory patterns (Gu et al., 2004; Conant and Wolfe, 2006; Tirosh and Barkai, 2007; Wapinski et al., 2007; Thompson et al., 2013), with one of the paralogs retaining the ancestral expression profile while the regulation of the other copy evolves more rapidly (Gu et al., 2005). This can be achieved by changes in the transcription factors that regulate the paralogs (Wapinski et al., 2010; Perez et al., 2014; Pougach et al., 2014), by loss and/or gain of different *cis*-regulatory elements in their promoters (Papp et al., 2003; Zhang et al., 2004; Ihmels et al., 2005) or by a combination of both mechanisms. The latter scenario might be the one at work in our case. Indeed, based on the observation that the Aft transcription factor of the pre-WGD species *K. lactis* fulfills the Aft1 but not the Aft2 functions, it was proposed that the mitochondrial and vacuolar control of iron homeostasis by Aft2 appeared as a neo-functionalization event after the WGD and the duplication of the Aft ancestral gene (Conde e Silva et al., 2009). Then, the acquisition of Aft2 regulation by *DOM34b* and *HBS1* would be concomitant to the emergence of the specific role of this transcription factor. Among the post-WGD species, the enrichment of Aft motifs in the promoters of the *DOM34b* and *HBS1* was especially clear in the *Nakaseomyces* sub-lineage which contains *C. glabrata* and two other potential human pathogens (*C. bracarensis* and *C. nivariensis*) (Gabaldon et al., 2013) (Figure 5 and Supplementary File S14), as compared to the other *Nakaseomyces* sub-clade or to the *Saccharomyces* genus. A similar pattern was also observed for *GRX3/4*, which showed a dramatic enrichment of Aft motifs in the promoters of the *GRX3* lineage in the *C. glabrata* sub-clade (Supplementary File S16). *C. glabrata, C. bracarensis, C. nivariensis* and their non-pathogenic relative *N. delphensis* were also the only yeast species to have two *MAK16* orthologs, with the promoter of the *MAK16b* paralog being enriched in Aft DNA motifs (Supplementary File S17). Hence, the presence of two *DOM34, GRX3/4* and *MAK16* orthologs and the co-regulation of *DOM34b, HBS1, GRX3*, and *MAK16b* by Aft2 may be a specificity of this *Nakaseomyces* sub-clade, which is enriched in hosts of the human body (three species out of four). To go further in the characterization of this regulation, it would be interesting to determine the actual contribution of the different Aft motifs in the promoters of the genes by site-directed mutagenesis.

Why were these new regulations fixed in *C. glabrata* and lost in *S. cerevisiae*? In other terms, how could these four genes contribute to iron homeostasis in *C. glabrata*? In *S. cerevisiae*, the cytosolic monothiol glutaredoxins Grx3 and Grx4 play a central role in communicating the mitochondrial iron status to Aft1 and Aft2 (Ojeda et al., 2006; Pujol-Carrion et al., 2006; Kumanovics et al., 2008). In iron replete cells, the mitochondrial Fe–S clusters biogenesis is very active and Fe–S clusters are exported to the cytosol where Grx3 and Grx4 form Fe–S bridged homodimers which has the capacity to transfer its Fe–S cluster to Aft1 and Aft2, therefore decreasing their DNA binding affinity by favoring the formation of Fe/S bridged Aft homodimers (Kumanovics et al., 2008; Ueta et al., 2012; Poor et al., 2014; Chi et al., 2018). This role of Grx3/4 proteins in the regulation of iron homeostasis is conserved from yeasts to humans (Li et al., 2012; Labbe et al., 2013; Jacques et al., 2014; Banci et al., 2015; Encinar del Dedo et al., 2015). It may seem counter-intuitive that *C. glabrata* Aft2 induces the expression of a protein which would negatively control its activity. However, besides its role in the regulation of Aft1/2, Grx3 (and its ohnolog Grx4) also makes important contributions to the oxidative stress response and to the cytosolic and nuclear Fe–S cluster protein assembly (Molina et al., 2004; Herrero et al., 2010; Muhlenhoff et al., 2010; Pujol-Carrion and de la Torre-Ruiz, 2010; Vall-Llaura et al., 2016; Pujol-Carrion and Torre-Ruiz, 2017).

In *S. cerevisiae, MAK16* was initially identified in a screen for the maintenance of the killer virus double stranded RNA genome (Wickner and Leibowitz, 1979; Icho et al., 1986). Later on, genetic, biochemical and cryoEM analyses showed that Mak16 is actually involved in the biogenesis of the 60S ribosomal particles (Ohtake and Wickner, 1995; Capowski and Tracy, 2003; Pellett and Tracy, 2006; Altvater et al., 2012; Kater et al., 2017; Zhou et al., 2018). *MAK16* is an essential gene, conserved from yeast to human (Kaback et al., 1984; Wickner et al., 1987). As mentioned above, *C. glabrata* and its three most closely related *Nakaseomyces* species (*C. nivariensis, N. delphensis* and *C. bracarensis*) have two *MAK16* paralogs, which we named here *MAK16a* and *MAK16b*. *MAK16b* was obviously not essential in *C. glabrata* since the *mak16bΔ* mutant cells grew at wild type rates in YPD (Figure 4). As all members of the ribosome biogenesis (RiBi) regulon, *MAK16* was repressed by stress in all the species we examined (Supplementary File S11). This was also true for *MAK16a* in *Candida glabrata*, which was consistently repressed by selenite and by all the other stresses that we tested (Supplementary File S11, unpublished data). Then, the dramatic induction of *MAK16b* by selenite, cadmium and BPS, and its regulation by Aft2, is particularly intriguing. The sole link that can be made between Mak16 proteins and stress responses based on the literature is the fact that they were proposed to form an atypical class of glutathione-*S* transferases (GSTs) (McGoldrick et al., 2005). GSTs are enzymes which assist the cell in the defense against reactive oxygen species (reviewed in Kalinina et al., 2014). Moreover, glutathione plays a key role in iron sensing by Grx3/4 and in iron–sulfur cluster assembly (Muhlenhoff et al., 2010; Kumar et al., 2011; Ueta et al., 2012; Martinez-Pastor et al., 2017; Cardenas-Rodriguez et al., 2018). Still, this annotation of *MAK16* orthologs as GSTs only relies on immunological criteria, i.e., the Mak16 protein of the trematode *Schistosoma mansoni* was probed by an anti-serum against purified *S. mansoni* GSTs (Milhon et al., 2000), and an actual GST activity was never proven for these proteins. Moreover, the fact that the deletion of *MAK16b* does not alter the growth of *C. glabrata* in iron starvation conditions makes its actual contribution to the iron homeostasis in this species questionable.

In contrast to *MAK16b, DOM34b*, and *HBS1* were required for optimal growth in iron starvation conditions, suggesting a role for these genes in the adaptation to iron limitation in *C. glabrata* (Figure 4). Dom34 and Hbs1 are also highly conserved proteins which are found in all eukaryotes, but they are not essential in yeasts. In *S. cerevisiae*, Dom34 and Hbs1, together with the general translation termination factor Rli1, were initially described as being responsible for a translation surveillance pathway called NGD, which rescues and recycles the ribosomes that stall before translation is completed (Doma and Parker, 2006; Passos et al., 2009; Simms et al., 2014; see Buskirk and Green, 2017; Simms et al., 2017, for recent reviews). Later on, the NGD co-factors were shown to be involved in ribosome biogenesis (Kispal et al., 2005; Yarunin et al., 2005; Cole et al., 2009; Lebaron et al., 2012; Strunk et al., 2012) and in the re-activation of hibernating ribosomes prior to quiescence exit (van den Elzen et al., 2014). Dom34 and Hbs1 also have a role in preventing ectopic translation of mRNA 3′ untranslated regions (Guydosh and Green, 2014), that is especially important in conditions in which the activity of Rli1 in regular translation termination is impaired (Young et al., 2015). In *S. cerevisiae*, the expression of *DOM34* and *HBS1* is not regulated by stress and these proteins have not been connected to the regulation of iron homeostasis. So, what could be the functional meaning of the strong induction of *DOM34b* and *HBS1* by BPS, selenite and cadmium in *C. glabrata*? Actually, a quite direct connection can be made between iron starvation and the Dom34-Hbs1 co-factor Rli1. Indeed, Rli1 is an essential Fe–S cluster containing protein (Paul et al., 2015). This iron–sulfur cluster is required for most of Rli1 functions and is very sensitive to redox stress (Kispal et al., 2005; Yarunin et al., 2005; Alhebshi et al., 2012). As a consequence, the number of unrecycled ribosomes in 3′UTRs increases upon treatment with oxidizing agents, such as diamide, which targets iron–sulfur cluster proteins (Guydosh and Green, 2014). Some authors even called Rli1 the “Achilles’ heel” of the cells in oxidizing conditions and hypothesized that Rli1 dysfunction is the main cause of cell death in acute oxidative stress (Alhebshi et al., 2012).

**FIGURE 6:**
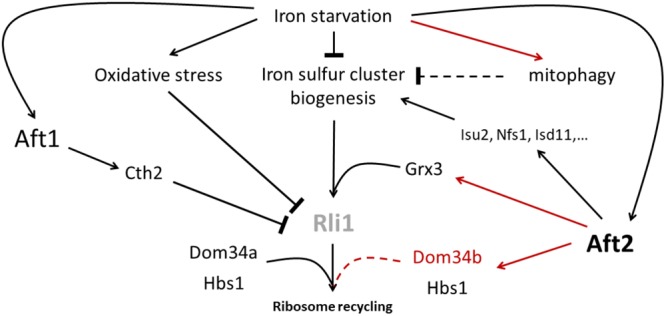
Hypothetical working model for the role of Dom34b and Hbs1 in iron starvation adaptation of *C. glabrata*. Iron starvation alters the functioning of the essential iron–sulfur cluster containing Rli1 in ribosome recycling at many levels: 1- by reducing the iron–sulfur cluster biogenesis potential, 2- by generating oxidative stress which was shown to strongly alter Rli1 activity, 3- by inducing the expression of the Cth2 RNA binding protein which targets part of the Rli1 mRNAs for degradation and 4- specifically in *C. glabrata*, by inducing mitophagy, which may reduce the mitochondrial biogenesis of iron–sulfur clusters. However, iron starvation also triggers the activation of Aft2 which may compensate the decreased activity of Rli1 by overexpressing 1- components of the mitochondrial core iron–sulfur cluster biogenesis machinery; 2- the glutaredoxin Grx3 which is involved in the delivery of iron–sulfur clusters to Rli1 in the cytosol and 3- the Dom34b and Hbs1 ribosome rescue factors, which, together with the constitutively expressed paralog Dom34a, may contribute to increase the ribosome recycling activity of Rli1. In this scheme, the red lines indicate the regulations which are specific to *C. glabrata* as compared to *S. cerevisiae*. The dashed lines indicate hypothetical activities which have not yet been supported by experimental data (namely: an impact of mitophagy on iron–sulfur cluster biogenesis and a role of Dom34b and Dom34a in ribosome rescue in *C. glabrata*).

Hence, iron starvation is likely to alter the essential Rli1 activity in ribosome rescue at many levels (Figure 6). Indeed, iron starvation has been shown to render yeast cells more sensitive to oxidative stress (Blaiseau et al., 2001; Matsuo et al., 2017). Moreover, the Fe–S cluster biogenesis, which takes place in the mitochondria, obviously depends on the iron supply (reviewed in Outten and Albetel, 2013). Finally, in *S. cerevisiae* and in *C. glabrata*, iron starvation provokes the Aft1-mediated overexpression of the RNA binding protein Cth2 which targets the Rli1 mRNA to degradation pathways (Puig et al., 2001, 2005; Gerwien et al., 2016). These effects may be particularly and specifically amplified in *C. glabrata* due to the active mitophagy triggered by iron limitation conditions (Nagi et al., 2016), which may also alter mitochondrial Fe–S cluster biogenesis. Thus, it is tempting to hypothesize that the transcriptional induction of *DOM34b* and *HBS1* would be a *C. glabrata* strategy to better cope with the decreased Rli1 activity caused by iron starvation (Figure 6). Interestingly enough, such a transcriptional up-regulation of *DOM34* and *HBS1* to compensate Rli1’s defects has already been described in human erythroblasts (Mills et al., 2016). When differentiating in red blood cells, erythroblasts face a difficult challenge: they completely get rid of their mitochondria and hence lose the capacity of producing iron–sulfur clusters, while having to maintain an active translation of hemoglobin, and therefore an active ribosome rescue process, in the last differentiation stages. They do so by transiently overexpressing Pelota (the human equivalent for *DOM34*) and HBS1L (the human ortholog of *HBS1*), which compensates for the progressive loss of active Rli1 (ABCE1 in Human) (Mills et al., 2016). Then, the hypothetical model that we propose for C. *glabrata* (Figure 6) would be analog to what was described for human erythroblasts. Of note, the induction of *GRX3* by Aft2 can also take place in this model, because Grx3 is involved in the transfer of the Fe–S cluster for the biogenesis of cytosolic and nuclear Fe–S proteins, such as Rli1 (Muhlenhoff et al., 2010) (Figure 6). This model is also consistent with our observation that in *C. glabrata* Aft2 targets several components of the core iron–sulfur cluster machinery. Obviously, many experiments, which go far beyond the scope of this study, will be required to test this working model. Especially, the actual contribution of Hbs1, Dom34a and Dom34b to ribosome rescue in *C. glabrata*, the precise role of Grx3 in the *C. glabrata* iron starvation response and the potential link between the regulation of these genes by Aft2 and the iron starvation-triggered mitophagy in *C. glabrata* will need to be thoroughly investigated.

## Author Contributions

MS, HR, and EC made the REGULOUT script and identified the ROs. LJ and SLC designed the microarrays. GeL, SB, and FD performed the multispecies microarray experiments. GaL, AT, and FD analyzed the microarray experiments. MB, LF, and TD built the strains and performed the Western blot analyzes. MB and LF made the growth assays in 96-well plates. AT and JM performed the multistress *C. glabrata* microarrays experiments. MB and AT performed the transcriptome analyses of the *aft2D* mutant strain. AT performed the ChIP experiments, the peak calling and the *cis*-regulatory motif enrichment analyzes. FD did the phylogenetic analyzes of the promoters, conceived the whole project, interpreted the results and wrote the manuscript.

## Conflict of Interest Statement

The authors declare that the research was conducted in the absence of any commercial or financial relationships that could be construed as a potential conflict of interest.

## Footnotes

1 www.yeastgenome.org

2 www.candidagenome.org

3 http://gryc.inra.fr/
