# Supplementary material for: Comparative Transcriptomics Highlights New Features of the Iron Starvation Response in the Human Pathogen Candida glabrata
Source: Front Microbiol. 2018 Nov 16;9:2689. doi: 10.3389/fmicb.2018.02689 (PMC6250833; doi:10.3389/fmicb.2018.02689)
Supplement: Supplementary file 6 [file Data_Sheet_1.PDF]

Supplementary file S1: Primers used in this study.

NAME	SEQUENCES (5'=>3')	Use
327-TRP1-For	TAC-GGC-ATT-GAT-ATC-GTC-CA	verification of deletions
772-CgAFT2Prom-del-For	GAAAGGATCGAGTTCTAAGGAC	Verification of aft2D
773-CgAFT2term-del-Rev	TCC-TTT-CAT-GTA-CAG-CAA-TGC-C	Verification of aft2D
773-CgAFT2term-del-Rev	TCC-TTT-CAT-GTA-CAG-CAA-TGC-C	Verification of Aft2 myc tagging
774-CgAFT2end-F1	ATT-ATT-TGA-CAA-GTA-TGT-TGA-TCT-ATC-AAC-CGT-AAT-GAT-TAA-TGA-TCC-TGC-GAA-GGA-AAC-TTA-CGA-CTC-TCT-TTT-TAG-TCG-GAT-CCC-CGG-GTT-AAT-TAA	Myc Tagging of Aft2
775-CgAFT2ter-R1	TAA-GAA-TAA-AAT-TAG-AAT-TAT-CTC-ACA-ACT-GTC-AAA-AAA-TCA-AGT-ATA-GTT-GTT-CAT-TTC-ACT-CGT-CTC-TCT-AAT-AAG-GGA-ATT-CGA-GCT-CGT-TTA-AAC	Myc Tagging of Aft2
792_pFA6a_verif1_For	GCT-AGG-ATA-CAG-TTC-TCA-CAT-C	Verification of Aft2 myc tagging
841MAKprimePromDelFor	ATT-GAA-TTG-AAA-GTT-ATT-TAG-CTG-TGT-GAA-AAT-AGA-AAT-TGG-CTG-TGT-TTT-CTT-CAT-TGT-AAA-TAT-TGG-ATT-TGA-GAA-GCG-GAT-CCC-CGG-GTT-AAT-TAA	deletion of MAK16b
842MAKprimeTermDelRev	TTG-ATT-ATT-TGA-AGA-TAT-TGA-ACA-AGG-TGT-CTG-TGG-CAC-AGC-ATA-TGG-ATA-GAC-GCC-TTG-GAA-TAT-ATA-TAT-TTT-GAG-AGA-ATT-CGA-GCT-CGT-TTA-AAC	deletion of MAK16b
846MAKprimeTermVerifDelRev	CAT-ATC-CTA-CAA-CTA-TAG-GTG-TAA-C	verification of MAK16b deletion
847MAKprimeORFSeqVerifFor	TCG-TCA-ACT-GAC-GAT-GAA-GGA-GAT-G	verification of MAK16b TAP tagging and deletion
894MAKprimeEndProtATrp1For	GGA-TAT-CAA-AAA-GAA-AAG-TAT-TGA-AGT-TGA-GCT-TGA-ATT-CGA-AAA-TGA-GAA-TGA-ACA-ACA-GCA-ATC-AAC-TGT-CTC-CAA-CTC-CAT-GGA-AAA-GAG-AAG	TAP-tagging of MAK16b
895MAKprimeTermProtApBSRev	TTG-ATT-ATT-TGA-AGA-TAT-TGA-ACA-AGG-TGT-CTG-TGG-CAC-AGC-ATA-TGG-ATA-GAC-GCC-TTG-GAA-TAT-ATA-TAT-TTT-GAG-ATA-CGA-CTC-ACT-ATA-GGG	TAP-tagging of MAK16b
914-TapGwenael-R	CGTCTACTTTCGGCGCCTG	verification of TAP-tagging
915-DOMlikeORFverif-For	TTC-GGG-TGT-TGA-GCT-ACA-GC	verification of DOM34b TAP tagging and deletion
916-HBS1ORFverif-For	AGC-TGC-CAT-CGT-TGA-GAT-CG	verification of HBS1 TAP tagging and deletion
918-DOM34ORFverif-For	GGT-GAA-GAG-CTA-GAT-AGG-ATG	verification of DOM34a TAP tagging and deletion
933-DOMlikeDel-F	CTT-TCA-GTC-CCA-TTA-GCG-TTT-AGT-TTA-GTT-AGT-CAA-AGA-GGT-TTA-TAG-AAT-TTA-GTG-TGG-AAT-AAC-TAG-CTC-AAA-TAC-GGA-TCC-CCG-GGT-TAA-TTA-A	deletion of DOM34b
934-DOMlikeDel-R	CTT-GAT-TTC-CAA-ACT-ATA-ATT-TAA-TCA-ATT-GAA-GAA-ATT-AAA-AAT-ACA-CAT-AGC-AGA-AAG-TAA-GCT-AAT-CTA-ATA-CCG-AAT-TCG-AGC-TCG-TTT-AAA-C	deletion of DOM34b
935-DOMlike-ProtAS-F	GTC-TTT-TGA-AAT-ACC-CAG-TTC-CTG-ATT-TAG-ATG-ATT-TAG-CAA-ATG-ATG-CAC-CAC-CAG-ATA-CTT-TAA-TTA-CTG-CGT-CCA-TGG-AAA-AGA-GAA-G	TAP-tagging of DOM34b
936-DOMlike-ProtAS-R	CTT-GAT-TTC-CAA-ACT-ATA-ATT-TAA-TCA-ATT-GAA-GAA-ATT-AAA-AAT-ACA-CAT-AGC-AGA-AAG-TAA-GCT-AAT-CTA-ATA-CCT-ACG-ACT-CAC-TAT-AGG-G	TAP-tagging of DOM34b
937-HBS1Del-F	TAT-AAG-AGT-AAT-ACC-CAC-AAT-TGC-CAA-TTC-ATG-ATA-AAG-TTG-AAT-AGA-ACC-AAC-CAG-ACA-ATT-GAT-TAA-CAA-CCG-GAT-CCC-CGG-GTT-AAT-TAA	deletion of HBS1
938-HBS1Del-R	GCT-GGG-GTT-TTT-GAA-GTG-CTG-TAC-AAT-AAT-GCA-AAT-TGG-TGT-GTC-ATT-GGT-GCG-GAG-TTA-ATT-GTC-TTC-ATT-AAA-GAA-TTC-GAG-CTC-GTT-TAA-AC	deletion of HBS1
939-HBS1-ProtAS-F	CAC-AAA-ATG-AAC-ACA-TAG-GAA-GAA-TTG-TGT-GCA-GAA-AAG-ATG-GAC-GTA-CTA-TTG-CCA-CAG-GTA-CTA-TAA-TGC-CGT-CCA-TGG-AAA-AGA-GAA-G	TAP-tagging of HBS1
940-HBS1-ProtAS-R	GCT-GGG-GTT-TTT-GAA-GTG-CTG-TAC-AAT-AAT-GCA-AAT-TGG-TGT-GTC-ATT-GGT-GCG-GAG-TTA-ATT-GTC-TTC-ATT-AAA-TAC-GAC-TCA-CTA-TAG-GG	TAP-tagging of HBS1
941-DOM34Del-F	GCC-ATA-GAA-TTG-AGA-GCG-AGT-GTC-AAT-TGA-AAT-ATA-GCC-TGA-GAA-AAG-CTG-AGT-GAA-AAT-ATT-TAG-GTA-GTA-TAC-GGA-TCC-CCG-GGT-TAA-TTA-A	deletion of DOM34a
942-DOM34Del-R	GCA-CAA-GCA-GCT-TTT-CAT-GCT-ATT-GAA-TCT-ACG-GGA-ATT-TAT-GGA-AGT-AGT-TAT-CGG-AGC-AAA-ATT-AAA-CTT-ATT-GAA-TTC-GAG-CTC-GTT-TAA-AC	deletion of DOM34a
948-DOM34-ProtAS-F	CTA-AAC-TAT-CCA-TTA-CCA-GAT-CTG-GAC-GAA-GAT-CTT-GAG-GAT-GAA-GAA-TCT-GAG-AAC-GAT-AAT-GAA-TTT-GAA-ATT-TCC-ATG-GAA-AAG-AGA-AG	TAP-tagging of DOM34a
949-DOM34-ProtAS-R	GCA-CAA-GCA-GCT-TTT-CAT-GCT-ATT-GAA-TCT-ACG-GGA-ATT-TAT-GGA-AGT-AGT-TAT-CGG-AGC-AAA-ATT-AAA-CTT-ATT-TAC-GAC-TCA-CTA-TAG-GG	TAP-tagging of DOM34a
962-DOMlikeDelLonguePromF	CTT-AGA-GAG-ATA-ACC-TCC-AGC	deletion of DOM34b
963-DOMlikeDelLonguePromR	TAT-TTG-AGC-TAG-TTA-TTC-CAC-ACT	deletion of DOM34b
964-DOMlikeDelLongueTermF	GGT-ATT-AGA-TTA-GCT-TAC-TTT-CAG-C	deletion of DOM34b
965-DOMlikeDelLongueTermR	AAG-ACC-ACT-AAT-TGC-ACA-TTG-TAC	deletion of DOM34b
966-DOMlikeDelLongueVerifTermR	GCA-AAT-TCC-TGA-TTC-GGT-ACT-TG	verification of DOM34b deletion
967-qPCR-DOMlikeProtA-R	CGC-AGT-AAT-TAA-AGT-ATC-TGG-TGG	verification of DOM34b deletion
968-HBS1DelLonguePromF	GAG-ACA-AGA-GCT-GTG-GTA-TAG-G	deletion of HBS1
969-HBS1DelLonguePromR	GTT-GTT-AAT-CAA-TTG-TCT-GGT-TGG	deletion of HBS1
970-HBS1DelLongueTermF	GAC-AAT-TAA-CTC-CGC-ACC-AAT-G	deletion of HBS1
971-HBS1DelLongueTermR	CTC-ACC-TCA-ATA-CTC-ATC-TCA-TC	deletion of HBS1
972-HBS1DelLongueVerifTermR	CAT-TAG-CCA-CAG-ACT-CTG-CAT-TC	verification of HBS1 deletion
973-qPCR-HBS1ProtA-R	ATA-GTA-CCT-GTG-GCA-ATA-GTA-CG	verification of HBS1 deletion
974-DOM34DelLonguePromF	GGA-TGT-TGG-TCT-TTA-GGA-CCT-G	deletion of DOM34a
975-DOM34DelLonguePromR	TTC-ACT-CAG-CTT-TTC-TCA-GGC-T	deletion of DOM34a
976-DOM34DelLongueTermF	ATT-TTG-CTC-CGA-TAA-CTA-CTT-CC	deletion of DOM34a
977-DOM34DelLongueTermR	AAC-TGC-CTC-TAA-GCT-ATG-CGA-C	deletion of DOM34a
978-DOM34DelLongueVerifTermR	CTC-AGA-ATG-GCA-AAG-ACA-CAA-GG	verification of DOM34a deletion
979-qPCR-DOM34ProtA-R	CGT-TCT-CAG-ATT-CTT-CAT-CCT-C	verification of DOM34a deletion
1074_QPCR-Makprime-R	GGA-GAC-AGT-TGA-TTG-CAT-ATG-G	verification of MAK16b deletion

	C. albicans	C. glabrata	D. hansenii	K. lactis	K. thermotolerans	S. cerevisiae	S. kluyveri	Y. lipolytica
annotated ORFs	6 205	5 209	6 230	5 073	5 092	5 902	5 321	6 409
designed ORFs	6 186	5 199	6 225	5 033	5 086	5 793	5 313	6 378
missing ORFs	19	10	5	40	6	109	8	31
Total probe number	49 152	43 159	48 951	40 408	40 577	45 771	42 849	51 730
average probe/ORF	8	8	8	8	8	8	8	8
% ORFs design	99,69%	99,81%	99,92%	99,21%	99,88%	98,15%	99,85%	99,52%
ORFs with complete expression profile	6120	5156	6084	5031	5054	5650	5285	6343
ORFs with orthogroup size >7	4022	3891	3916	3695	3668	4239	3805	3925
% ORFs in REGULOUT	64,82%	74,70%	62,86%	72,84%	72,03%	71,82%	71,51%	61,24%

Supplementary file S2: Features of the microarray design for each species: number of annotated ORF in the databases, number of ORFs for which the probe design was successfull, number of missing ORFs, percentage of probed ORFs. Features of the expression dataset: number of ORFs with complete expression profiles measurements, number of remaining ORFs after the REGULOUT filter on the size of the orthogroupes, percentage of ORFs considered in the REGULOUT analysis (as compared to the total number of annotated ORFs).

Supplementary file S3: distribution of the number of sequences per orthogroup

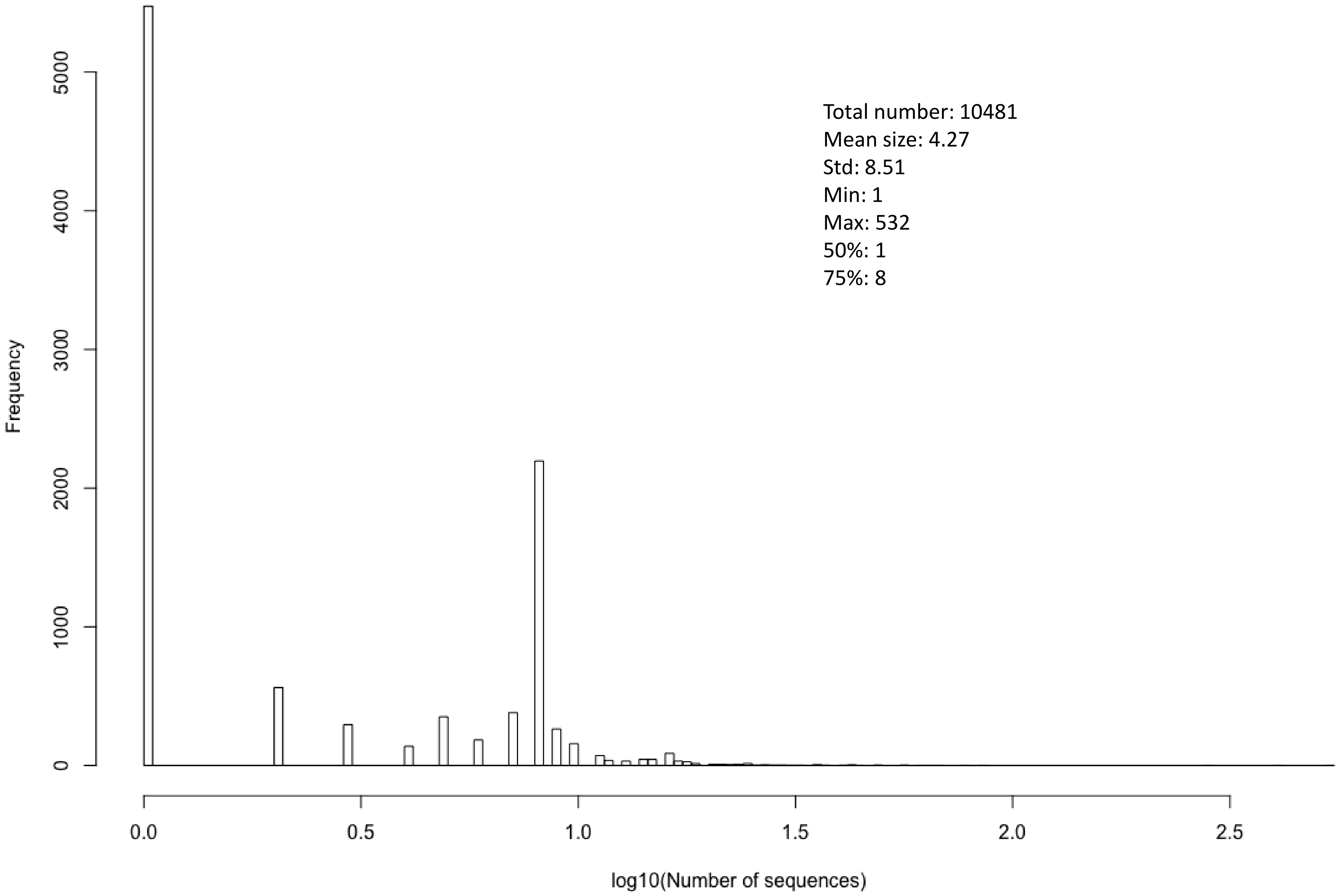

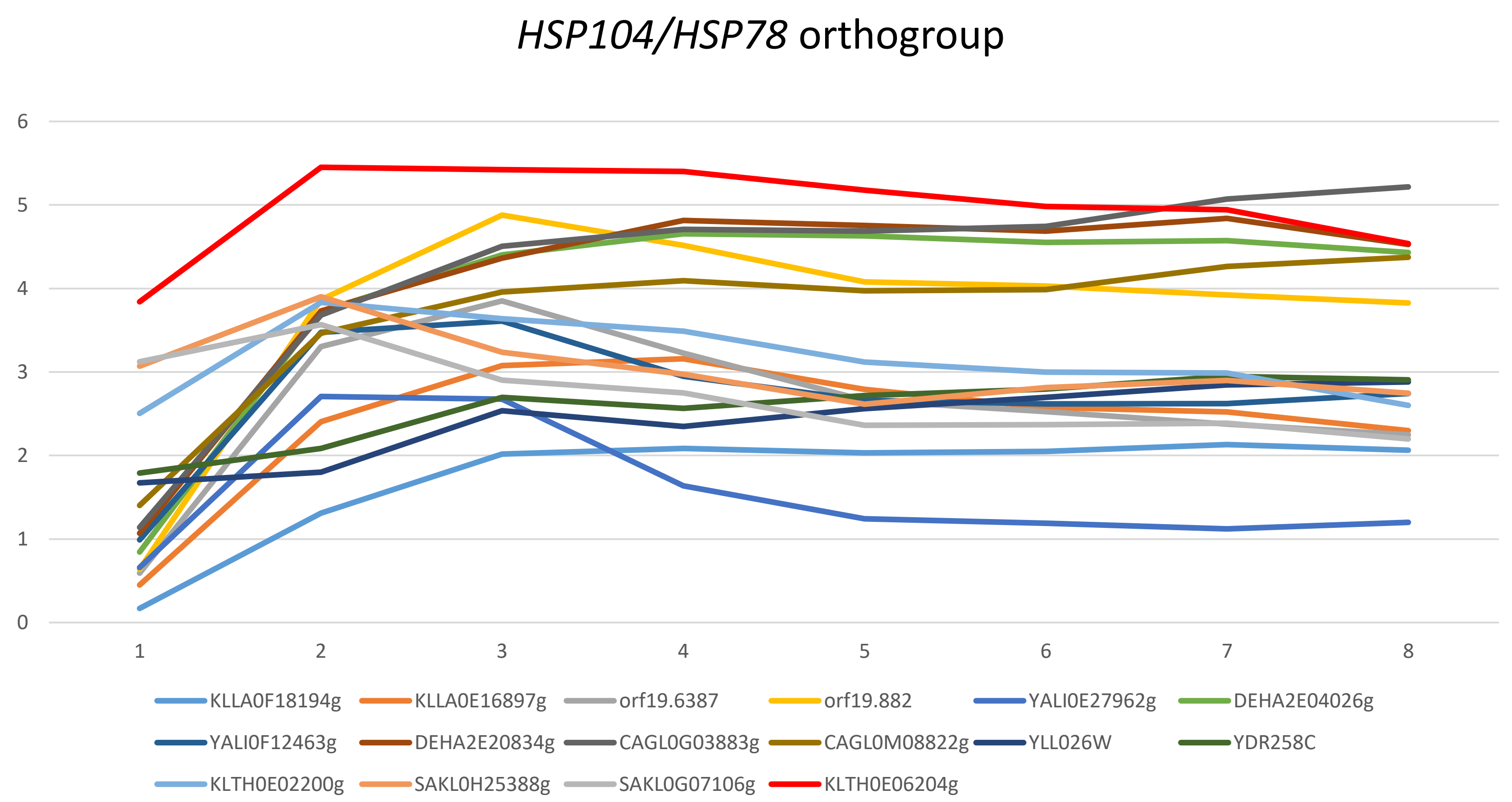

Supplementary file S4: The *HSP104/HSP78* orthogroup as an example of the REGULOUT false positives which were removed from the final list presented in supplementary table T2. All members of this orthogroup are strongly and rapidly induced by selenite with no gene having a completely different profile from all the others. Still, because euclidian distance is very sensitive to stochastic variations for genes with large amplitudes in their expression changes, the *KLTH0E06204g* gene (bright red line) was considered as a RO by REGULOUT.

Supplementary file S5: Sequences of the promoters from the *DOM34a/b* orthologues. The Aft2-like motifs are highlighted in red/orange. The Aft1-like motifs are highlighted in bright/dark green.

Prom *DOM34a C. bracarensis* reverse
GTATGTCGACCCTCCGTTTGCACCAGGTTCTGCCTTCAAGACTGCTTAATT
TAGAGATAGATGGCCATTAAAACTGCCATATTATATAAAAAAACGTCTTATATGCCCAAA
GCAGAGCATCTGTTACCCGGATACTCCACAGAAACTCCAGTAGCAACTTCTATTAATTTA
TTCAATATATAACCATTATCCAATGATGTCCAATGGTGATGCTACAGCTGCTATATTGAT
TCATTGTACTGGTTGTAATTGGGTATTTATGAAGTTCATTTGCTCTGTTTAGAAGTTCAT
TGCTTTGTGGAATTTGAGGCTCAAGGACATCCTCTATTGATACAGTACTACTAGTTACAG
TACACTATTCACATAGAGCATAATTATTCTAGTCACGTAAATATGTTTTATTGGGTCCTT
TTATATATTAAATATGTTTTAGTTTAGCCTTATACGGGAGGGGAGGGGATAGATTAACAA
CTCATATGCTCTTCTAGCTTTCTTGCTCTTTCTAATAGTTCTTACTCCACGAATTCAGGT

Prom *DOM34b C. bracarensis* direct
ATTAACTTCTACAAGTGTTAACAATGGTTGTTCCCCCTACTTTATTGGCCCCAGAAGCAA
CGTAAGTCTACCGATTAAGGAAAAT**GGGTGT**CTGCCATAGAAATATAGAGAAAAAAGAAA
TTTAGGAAAGCAGTTCAAATGGAAGAGCATTAGTAATCTTAATCCACTTTTTAAAACTCC
ATGAAGCAAGGTTATTAGGTGAGGTGCATTTGCACAACTCGTACCTTTGTATCGTTCCTT
TCTTTTTTTCTAACATATAAGTCAAATT**ACACCC**ATATTTTCAGGTACTTTATATAAGTT
GTTAATTATATATATATATTTGGGCCTTTGCTATACAAGTAAGTTTCCTGAGGTTAATAA
GATGCTTGATTTACTGTTACCTATTAGAACTTGTATATATTATCATTTTGTTACGTACTC
ACATTAATATTTAGGTAGTTATTTAGCGCCATATACCTTATAAGAGTCATATATAACTAC
TAGGAAAGGATAAAG

Prom *DOM34b N. delphensis* direct
GTCTCTAATTAAATGTGTTTCTTTAATTTCATGGTGGTTGCAATTTTTTTATGCCTTATT
TTTTAGATGGCTTAACTTTTTCACTTCTAGTCAATAAGCAGATGG**GGGTGT**CTAAAGAAT
ATCCTCGTCT**GGGTGT**CTGACTATTTATTTTAGTAGCTATTAAGAGAGTTTAAGTGAGTT
TAGCTTGCATGCTCTAGAGCATGATGCGATG**GGGTGT**GGGACTACCATCATCGATTTGAA
CAATATCAATTTTTTATTCTTTCTTTCTTTTTTTTTTTATCCAATTT**ACACCC**TTGTTTTA
ATGAATATATTAATAATCTGCTTAACAATTTATGCCATATATAAGTATCATTTTGACAGT
ATAATCAAAGAAGATCAGGAGAAATAGTTCTTGGCTGCATCTTCTTATATCTATTCTTCT
TCAACGTATATAAAAAATTAAAGATATAAGGTAACGTTTAGACACTTAATACTGAAGTAT
TAATAGTAAATTAAAAG

Prom *DOM34b C. nivariensis* direct
AGGTTGTAATAAAGCCCACTCTGTACTTTGATATAATATTGTGTAATAAAACTCTTATAC
TTAATAAACTTTTCTCTTGTTATAAAATAAGCTGCCGGAGTACCAGATTAAACATTTTGA
TTTCCACACCGATGAGATGGTCATTGAG**GGGTGT**CTGGCTCAA**GGGTGC**AAGGAAGAAAA
CAGAAATGATCTTTTTGATTTTATTTAATCAAAAACTGTTTGTTACTGAAATTGTTGGAT
ACTGCGATG**GGGTGC**AAGAATGCATGAATGCAGTATTTCTTTTATTTTTTTTCTTGCCAA
TTTTTCTTTTTAAAAGCAATTT**ACACCC**ATGTTTCTAATAACAACAATTAATAATCAGAA
GATGTCTACTTGAATGCAAAGGATATAAAAGACAATCATATGCTAAATGTACATAATTTT
TGTTATATAGTTTTATTTTAGGAATTTTTTATTTATTTTTTTCTTTAATGTTTTGTACCC
ACTTACAGTGTAAACTAGCGCTAAGTATTTAAAATAGTAATAACGAAATA

Prom *DOM34a C. glabrata* direct
AAGTAAAACATTTGGGCATTATAGTAGTAATATCATATCGGCGTATAGAATATCATATAG
CTGATAACATGTGGGATATTAAATTTAATGTTTTTATCAGCATTCCCTTTGTGTTATCAG
CTTAGTTTAGTTTAAGTCGAATTTTCATGCCATTCATGACCGATGCAATTTGAACTAGTA
AGAAATGGTGTTTTACATTTGATTATTGGCTCTACTGAATGCATTGCTGTAGTGTAACTA
CTTTCTGCTAGAGATCTATCGAGGTTGATCAAGATGTCAACCTACTGCTTATAATTATGT
ATAGTTGCATCATTTTGTGCGATGGTTTCTGTATGAAAGAATCATAGCCCATCACTTCAA
GTAATTTTTTATAGTGAAAACTGAATAATAGTCCAACTCAAACTGTTGCAGTAGTATAAA
AGTAGTGGTAATTGATCATTAACACTGCCATAGAATTGAGAGCGAGTGTCAATTGAAATA
TAGCCTGAGAAAAGCTGAGTGAAAATATTTAGGTAGTATAATG

Prom *DOM34b C. glabrata* direct
GCACACAGATGATACATATACAATTGTAGTTGTGGAGATGGCGCTGTAAAATCTACAATG

GAGTGATCTAAACTGTCACAGTCATGAAGGCTACCACTATTCAATATGCGAACACTCGCA
GTTTTCGTAATCAAAAATCCATCGATACAATAACTAGTATACTTTGGAACAATAACATTT
ACTGATCAACCCAACTACCGCAGCATAAACTACTTCATCTTGTGTTCGAATTTTGAGTAT
CGTGATAAGAACTTTTTGTTATTCGGATGG**GGGTGC**GAGATGAGATGGTTCATTTGGCTT
TAAA**GCACCC**ATTTCTTGG**ACACCC**CCCGAATGTTTTACGTATGAGCATAATAAAATGCA
CTTGCCTTGTAATATAACTTGAACAAGGTTGAATATATAAAATGTACATTTGGATGCTGC
TGGAGTGGGCCCTATATAGTTTTCTTTCAGTCCCATTAGCGTTTAGTTTAGTTAGTCAAA
GAGGTTTATAGAATTTAGTGTGGAATAACTAGCTCAAATA

Prom *DOM34a C. nivariensis* direct
GGGCTATTACCTATCTATGCCAGCTTCATCCATTAGTAGTACCACGTCGTTCTGATCTAA
GACCGGAATTCGTCGAGTAATCCCCCTTCTTTTATTGCTAAAAAAACAGAAAAAAAAATA
GTATATAACATGTATAATATAATATTGTCACTTATTTGTGCTTGACGTTATGTGTTCATT
AAATCATTGTCACGCTGTGCATCATGACAGCAGTGTGCTCCAAGAACATGAGAAGGAAAT
CATTGGCACAATAGTGAAAGGTTAGAGAGTACGGGGAAACTCTCTAGATTTGATTGTTAC
AAGTACAGAGTATCTTGGCCATCGTTACAACATACAAATGCGTTTATCTATAGTATATAT
AGCCCTTTATGAAGAGTTTCATTACAATCTGCTAAGTAAGTTTAAAAAAATTTTTTTTTC
AATTTTTCTAATTGCGATTCTTAAAACAAAAATATTACACTAACATTACAAGTTTGCTAT
TTGATACCTAATATACCCACTGCTACAGCA

Prom *DOM34a N. delphensis* direct
GATATTATTTGATGTAACGATTTTTTTATCTAATTATTTCTGTATAATATAATCATTCCC
ATTTACGTTGCAAGAGTCTCACATACTAAATAGAATCTCCTCTTCTCTGCTAAAGAATTC
AAGACATTGGTAGGCTCGTTAATTTGTATTTTACAAAACCTATTTCATAAAATAAAGTAC
TAACCTGGTTGTGAGGGAAGAGGCCCGAAATCCAATAGCCACCCCAATAATGTCTTTCAT
TGTTAACACCAAACGTCCATCAACGTTTGAAATAATATCTATTATATACTCAATATCTAT
TATATTCAATATTTTTTGCTGGCAATTTGCGATTCTGCGAGACCCATCTCAAAATAAAAA
ATCTTTCAATCTTTTATATTTTTGGTTGCAAGCAAGTATCCAATAACTAAACGCTGTGCA
CGCATACAAATGTCATCACGGTATAGTCTCAATTGAATCAAGGTTTTTGGCTAACTTATT
TACACGGGAAACATATAGAGGGAGTATATTCGAAGAA

Prom *DOM34a?b? N. bacillisporus* reverse
AGCTCAATATTGTTG
CGTATTTCCT**GGGTGT**GGAGGTTTAAAAGTCCTGAATGCTGCCTTTCATGTCTTGAATAA
ATCGCAATTAGTCGCATCTATATACTTTTGAAATAATATTGGGTGAATAATCAAAAATGC
TTGAAAAAGGCATTAG**ACACCC**AATTAAAAAAATAATAATAGTATAACAAACATTTATGTA
TAAGACCTGACATGCATATGTCTATCTATATATGGATATATATGGATATATATATATATG
TGAACTCTATCCAGAGGTGTACTTCTTTTTACCAATGTCCAAAGGTATGTACTTGTATTT
GATCATATGCTGGATTTGTCTTCTCATGGTTATTGAGAACAACAAAACGAAATAGATAAG
CAAGATGGGCCAAAACACAGGTATGTCAGAGATACGAGTGACTGTGAGGACAAGAGATAA
AGCGACCGCCCTGGCGGAATTGTACCAGAACTTAAATTCTGGCAACCTTCTGATAAAAGG

Prom *DOM34a?b? C. castellii* direct
ATGAATCGATACCTATCATTAGTTTCTGCATCAGCTTACGATGAGAACTCGCAGTAACTT
TAATATTGTACAAAGATGTATAGGGCAACAGCCCAAGATTGTATATAATATGATTAGTGC
TAGATGGTATTCGAGACAAATATAAGATTTCAACTATCGCATGTGGATCACCCATATATT
TGAGAATATGAACAGTACCACTACGGCAAGGTAAAATCTCATGTTGGTATTTTCATATAT
TACGTGAGTTGATATTTAAGATATATTCTACTCTAACTCAACCTCGTAAACCCCATGT**AC**
ACCCTTGGTGTTTTTCTGAATATTAATAGTTCATAATGTCTAAACTGACACCTCGGCTGC
AATGAGAAATACACATCAAAATAGATCTAGGGTTTTTTGGTTATTTGAGTAGTTAAAGCT
AACTTAGACACAAACTACTTATACAATAAAACCAACAAAAGTAACGAACAAAGAAGTTAA
TAGG

Prom *DOM34 S. cerevisiae* direct
AGAGGTAGCCAAAGTCTTTGCAACTATACTTTCAGCTCTGACAAATTTGTTCTTATTACT
TCTCTTTTTTTTTGATTTGTTCTTCCCTCTTTTTCTTAGCTAATTCTTGTCTTTCGATTCT
AGTTCTATCAGCATTTCTTCTTTTCCTCAAAAGAATTTCTGGATTAGAATTAAGAGTTTG
CGCTTTACTGTCTTGAGTTGAAGACATTGTGACTTGTATATCTTATTCTTTTTCCCTGTG
GCAGCCTCTTCTTTCTAGGAACTTAATAACGAAAGTACCAAACAACTTAGACTTTTCTCA
TCTCATCGCTTCCCAGCTTTTTTTAAGCAGAGCTGAAAAAAATTTCTTTTTACCAAAAA
TCCGTACTGCGTGATGAAAGTGTACATACATTATTCAATATTCACCTAGTTTTGTATATT

AATGTAAAATGTAATTTAATGAAGATCCCAAAAAATTAAGCATTCGTTGCTGCATCGTTG
TCATTTTGTTCAATTATCGCATTCCTATCATAGCAAAAAT

Prom *YCL001w-a S. cerevisiae* direct
ACAGAATAATTGTGATTATTTTCTCCTTCTACTCATCTTCTCTTATATTGGAAGAATACA
TGAAGTTATTGATTAAGCAAAATTGAAGGGAAGAGGCTCATTTTCTTCCATTTATAAATG
GAGAGCTTGAGATGCCTCCAATTGATAAATTTTTGTAAAGACTTTCAAGAAACATGTCTA
AAAAGAATTGACAGCTTCGAACCTTTCTGCATAATTACTAATACCAAATTAGTGAGAAAT
GGGGTATGTCGAGGAATAAAGGATGCAAAAGAGAGAGTAATGTCATGCAAGTTGTGAGTT
TGAAATGTTCACACGTTTATAATGTCCATACTCCGTGGCGTGCATGTGTTTTAGTGTATT
AGTTTTCCAAGTTATTAAAGAACTTAGTAAATTTATCATCGAACGCATTTTGATTAAGTG
GCACAATAGACTTCAATGGAAAATGAAAATCATATCTGTAGCAAAAACGACAGCGAAGAT
AACGGTTACACAATTATCACGTTGATTCGTCAACTTAAAG

Prom *DOM34b N. castellii NCAS_0C05020* direct
CCAACACATGATCAAATATAGATACATCCCATTAGATATTGGTAAGAAGAGATACAATTC
TTCCGCTTAGAGGTTTGCTTTAAACAACCTGTATAGACATTGTTCACCTTGGCTCAAAAA
AATTAAAATAAAATAAAATACAATAAAAAAAACGAATAAGACAATTCAAAAGAGGATGGA
TGAATG**GCACCC**TGGTTTTCCATTGATAGTTCACAGGTTGCTTACGGACATACGTACGTA
CGTATGTGTGTGCTTGACACATTTTGTTTGGGCTTATTAAGCTGTTCTACTTTAAAAGTT
AAAAGT**GGGTGT**AGGATGCACGGAAGTTGTGTCCATTACGAATAATAGCATTAATCCACC
TAAAAGTGTCTATATACCTACACACACATATATTTTTATATATATATATAAGCATGCGTA
TTCGATTTGTAACTACCTCATCGTCATTTCTTTCTATAGTCATATTTCTTCCTGTCATGC
ATCAACTGTAACAAATTATAGAAAATAAGCACT

Prom *DOM34a N. castellii NCAS_0G03270* direct
CGGCAAGTACTTTCAAATCTTTTAATCTCGAGGAACGCACTTTGAAGGCTTGAGATTTAC
CTCCATCACTAAAAGTAACATTATTTAACGTTAGACCCTTATTAGTAGCTTTCGCAATTT
TCCCTGTGATCAGTTTCCCGTCTTTCAGTTCCACTTGAACTTCAAATCCTATAAATTGGG
ACATTATGAGTTGTCTATTGGTATAGTGTAGACAGGGTTCAAAGAAATTCTGGTAGATAT
TTGCTCTAGATTCCAGGTTATTTGATGAATGAAACAAGTTTCTCAAGCTGTTATTGAATG
TTCAACTGTTTTTTTTGAAAACGTCACGTGGAACGAAGTTGTTTAAATAGCTGCTAATAA
TCTACATTAACAATGAAGGATGCTCGAATCGCAACTGAAAATTTAATCTTATACGAACAT
AATAGTTAACAGGGACCAATCGAATATTGAAATTGAAATAAGCTATCTAACTTTATTTGC
AATATTTTTACGAAAAGAGAAA

Prom *DOM34a?b? T. blattae* reverse
TCTGATTCAATGGTGATTG
CCTCTAGTCATAACTTTAGTAAAGGTTCCCCCTCTCACTTTTTTTCACTTTTTTTTCATT
TTTTTTTATACCATCTGTAAATCTAAAAACATCACAATGGCATTCGCCCGAATTACTAAC
C**ACACCC**TCATTCTATCTATGCATTACACCTACAAAATTCACTATACATACACCTATTAA
TTCCTGGCTACATAACTTTTCTTACCAATATCTAATGGTATATACTTGTATTTCATCATA
TGTTGGATTTGTCTTCTCATTGTTAGGAAAAACAAAATGATAAAATAAACAACTAACACG
GGCCAAAATACAGGGATATCAAATACTTGGAAACACGACATCACCACACAAACTAATGTG
GCTCTCATCGAATTGTACCAGAATTTAAATTCAGGTAATCTTCTAATGAAGGGGCGGAAT
TCTTCACTTCTTTCCCCAGATTCCAATTCTTTGTTCTCTTCATCTTGTTGTAATGACATA

Prom *DOM34 S. uvarum* direct
TTTAACCTGATTTCTTGTACTCTTTTTGCTTCTATTAGCTCTCTCCTTGAAGAAACTTTTTATTAAAAGATGTAA
GTAATCGAGGTTTTTCTCATCTCATCGCTTCTTTTTNNNNNNNNNNNNNNNNNNNNNNNNNNNNNNNNNGCTTCTT
TTTTTTTTTTTGTAAGCAATGGTGAAAAAATTTCTTTTTCAGGTACAAACAATCCGTACAGTATGATAAACTA
ATGAAATATTGAATATACATTAGAAAATGTAAAGCTCTTTATGATTGTTACTTCTACGTATATAATGTTCGCTGA
GGAAGCAAGCGAAAATTAAGCCGACTGCCTAGGGATTATTTTTTGTTTTTCTTTCGTTTTGTATCTGTAACTTAA
AGTATCGCACGTCACCTCCGAAAGCT

Prom *DOM34 S. mikatae* direct
TATGATTTATGTTATTAACTCTTCGTGCTTTTCTCAGTCTCTATTTATTAAAGACGGTTCTCAAAAATATTAAGC
TATTAAGACTCTTCTCATCTCATCGCTTCCGATTTCTTTCAAGCAAAGGTGAAAAAAATTTCTTTCTTGCAAAA
AACCCCACTCCGTACAGTGTGATGGAGTTAATACTTTTAAACATGCTCCTCAGGACCTGAAGTTTTTTAATACCA
TTAATGAGAATAGCATAACAATTGCAAGAAAATTGAAAATTCATTGCCTCTACAGTGTTATTGCGTCATTTAGGT
AAATTATTGCATTTCTCTCACAAAAAGCT

Prom *DOM34 S. paradoxus* direct
TGTGGCTTGTATTTCTTACTCTTCTTCCTTGTCTCAGCCTCTTCTTTCTAGAAACTTAACAACAAAAGTATCAAA
CAATTGAGACTTATCTCATCTCATCGCTTCTCATCTTTTTTTTAAAGCAAAGTTGAAAAAAATTTTCTTTTTATG
AAAAAAATCCGTACAGTGTGACGTAAGTAAACACCATTAAATGCTGCACCTCAGAGAGTAAAGTTTTTTACTAAC
ATTAACTGTAAATTGATATAATGATCGCAAGAAAATTAAACGGGTCATTGTCCCTATAGTATTGTTGTTGCGCCT
ATAATTTCTTTGTCTAATTATTGCATTTTTATCACGATAAACT

Prom *DOM34 S. kudriavzevii* direct
TGTGATTTAGTTTTCTTAATCTTCTGTTTCTTTTAGCTCCTTCTTTCACAAAGTTTCTGCTAGTATTATTAATCG
AGATTTTTCTCATCTCATCGCTTCTTTATTGGGCTAAGGTGAAAAAATTTTCTTTTGTAGGTACAAAGATCCCGT
ACATCGTGATGAAGTAATATTATTGAATATTTACTTTATAAAGTAAAGTTTCACATAGTCATTACTTCCACTTAG
CATAGAATTCGCGGATAAGTTAAATAAAAACCACTTAAACATTTAATAAATTGCGTCTTTACATTGTTTTGCCCA
ATTACCGCAATTCACCCAGAGCAAGTT

Prom *YCL001w-a S. uvarum* direct
TATTCTTGGAATCCACATACCTCTTCAATGGACACATTCGTATTTTTTTAACTATATATAAATAAAGAAATTTGA
TCATTCAAAAGATTTTGTTTTCTTTCCTTTTGCTGCCTGTACCAAATGTTTTTTTGAAAATTATATACCATTTAC
ATATAAGGTCGGTGAGAAGGATCCATTTTTGATCATCATGTATAGATAAGCGTTTCAAAATATTCCAAGA**ACACC**
CACTGTTTTAAATCTATTACCAACCGTCTGTTATGTTTAGTTTTATGTT**GGGTGT**ATTCATTAGTTTCTAACCTT
AACAATGAGGCCTGCAACCTAAAATAATGAGAAATG**GGGTGT**GTCAAAAATATGGGGTGCAGAAGGAAGAGCAGG
CTAAAATATGAGAGTTGAAAATCAATTAACAGGTCACGACTTTACACCGCTCATATATAAGATAAAGCGTACCCT
GTATTTGCCAAATTCATTATGAAGGATCCAATAATCTGTTATTAAAACAATTTAAATAAGTGCCTCAGCGGGATC
TAAAAAGCA

Prom *YCL001w-a S. mikatae* direct
TATTCCCGGACTCCACATACTTATTTAGCGAACACATTCGTATTTTTAATTGTACTCAAATCAAATTATGAGGTC
TTCAGATGATGATTTTCATTATCTTCTCTTTCTATCCACCTCTTTTATGCCTTGAGGAATATCCAATTTCTGATT
ACTTAAAATCGAAGAGAGTACAGTACGGCACAATTTCTGCCGTTTATGAATGAAAAGTGGCAGATATCCAAATTA
AGCCGTGATTGCAAAATTATTTATTCAAGTGTTTTATATGGGTCAATAGGTTCGAGTTTCGCTTAATAATGAACT
CAGCTATCCGAGAAGTTTGAATGAGACATAAAAGAGGAATAGGAAGTTGGACGTCCATTTTACTTCTACATTGAA
AATATCTATATAAAATCTGTGTCAGGCATGGGTTCCTCAAGATTTAGAAGAATTTAGTGGATTTTATTGTCGAAC
ACATTCCAATGAAACCGTACGATAGGCTTCAAAAGACAATGAGTAATTTATCAATAGTTAATAAAAGACTTTAAA
AATAATGATAATTGAATT

Prom *YCL001w-a S. paradoxus* direct
TATGTGGACTCCACATACCTATTCAACGAACACATTCGTATTTTAATTATATGTAAATCAAATACCTGATAGTTA
CAGAATAATTGTTATTACTTTCTTCTACACATCCTTTTTTATATTTGGAAGAGTACATGAAATTATTGATTAGGT
AATTGACAGAAAGAGGCTCAATTTCTGCCATTCATGGATAAACAGCTCCAAATGGCTCCAATTGACCATGTTTTG
TAAAGGTTTTGAGGCGACTGTCTTATATAGATCGACATCTTCAAGCCATCGCTCAATACTGAGCTTAGTTGCCAA
ATTAGTGAGAAATG**GGGTGT**GTCGAGGAATAGAGGGTGCAAAAAGAGAGGGCAATTTGCCATGGAAGGTGAGAGT
TCGTTAAATGTTCATACTTGACAATGTCCATATATAATCGGTGACATGCATGAGTTAAATGCATGCGCCTCCCAA
GAACTTTACCATTGAACGAAATTAATTAAGCTCCACGATAGACTTCAATAGACAATGAAAATCATAACCGTGGCA
GAAGACTCCGCAGATAACGATAATTCAATCATCACGTTAATCCCTCAAAATAAAGATGATCTTTTTACGATTTAT
CAAATAATAGACAAAGAGGATGAAGTC

Prom *YCL001w-a S. kudriavzevii* direct
TATTTTTGGACTTCACATACTTATTCAACGAACACATTCGTACTTTCAATTTTATGTAGATCAAGTCACCTGATC
ATTCACAATATTATTATTCTCATTTTCCACCGCCTTCTTATTTGAAAACACATACGCAAATATGAATAACGTAGA
ATTGCCCAAAGAAGCTCATTTTTCTGATCATTCACAAGTAAAATATTATAGACGGGTTAATTAGAATCTTCATCT
ATCAGTTTTTCGAAAACCTTCGGGTTCAGCTATTTTATATGAGGTTAGCAGCTTCTAGACTTTGCTCGATAAACA
GTGAGAAATG**GGGTGT**GTCAAGAAATAGAGGGTGCAAAAGGAAAACAGGTTTCTTATGAAAAGTTGGGAATTCAT
CACACGTTGATAACCGATAGCGTTCATATATAAGTGGAGGCATGCTCTATGCTATCAAAAGTCTTCTGAAAAATT
CAAAGAATTTTAAGAATTTTAACTCTGAACAAATTAGATAAAGCGCTTGATAGACACTAAAAGGCA

Prom *DOM34a K. africana KAFR_0H03070* reverse
GAATGGAACTACCTTATCAATGCTTCTCAACGACTTGACCTTGTTATGAGAAAATGA
AATAATTTTCTTTTATGCTGCGCTGAAAAATCATGAAGTAGTTTTCCAATTTCGCCCATAGGATAAGATG
AGATGGTAGGATATTGAAATTTGAATGCTATTGACCAGTTTCTCAAAAACCTCTCTTATTGCTATGCATC
GAATGAAATTCAAATTTGAGGGAGAATTGTCTTTTAATAATTTCACGATGACTTCCTCTCAGAGAGGTGT
TTTCTTAACTGGAATATGAGTTGCGCTGTCAGTCCACCTCAAACAAACTTTTGTATAAAATTTAGAAATA

AGGCACTAGAACCCTGAATCAAATGTACGCTTTTATTTATATATAGTATATCTACTTATATGATTGGGAA
ATGGTATTCTACACTCTATTAACTTTTGTTTATTGGGTATCAAAACCCTATGCTACATTTAGGCTAAGCA
TACTTTGTTAAGAGCCTTATTTTGCTGAACTTCCGTACTTTCTTTTGCCAATATCAAGTGGAATATACTT
ATACTTCACCATATGTTGAATTTGTCTTCTCATAGTCAATAAGAACAAAATTATGAAATACATCAGTAGA

Prom *DOM34b K. africana KAFR0I02320* direct
GATTAAATGTCAAACGATGACATGGATAATGAAATTGCACCGTCTTTTATGTACTGAGAAAACCCATTGT
GACCAGCATTCTATCCAAACGAAGACATACTTTTGAATCTTGCGTTCGAACTTCTGTTTCCCTTATCGAG
TACATGACACTAGTCGATTACATAATTACAATTTATTAAATACTAAATGAGATGAGCTACAAAGAAATAG
TTAACATGGCTAGACGTGCTATCGTACGTTGAAGCTGATCAAGGTAACTCGTCCATCCTGCAGTAGGCTG
TAAATACCTCTTTAGTATTTGTACACTCCCGAGTGTCAGAAGTATCATGAGAGCGGAAAGAGTATTGGAC
CATAAACCCGCCGCAGAGATGATCTTCTAACGTATAGTAAAACTCAGTGCTACTTCAAGCATTATCGTCA
TATCTATATATGTGAACTATCTTTTTCTTGCCATTTGACTTCATGTCATAATAATTA**GGGTGC**ACTAATT
AATAAGCGATTTATTAGTG**ACACCC**CAAAACTAACAAAATTCTATATAAAGAGAGATGTGAATAAAACGT
TGGTTTTGGTCAGCAAATGTCGTAGTAGCTAAAACCAAGTCAAG

Prom *DOM34 L. cidri* direct
CAAGAGCAACTCTGATCTCCCTACTCATGACTTTTTTCAACATATTTGATATACCTGTAT
TTTGGCCGATCCTAGTGATGTACTTCATTCTTCTATTTGCCTTGACAATGAGGAGACAAA
TACAGCATATGATCAAGTATAGGTACGTTCCACTAGACATAGGGAAAAAGAAGTACAACG
CTAGTAAATGAGTAGACAATGGTCAAGATCTGAAATATATAAGAATTTTTCTTTTACATC
TGATACGTCATAGTGTAATCTTTCCTAGTTTAAAGGCTCTAGGCAATACTAATGTCTTAT
GTAGAGTATCTACTACAGGCTTATCCTCCAATTAGCACTACTTTATTTATCACTGTCACA
TATTGGCGATATTACCTTCATCTTCATTTGTCCCTTCAAACGATGAAACATGACAAGTTG
CACTTATTAAAGAGTTCATTATCGCTTCTTGTCAATATCACAGCAATCAGCGTTAATTGC
GGCATTCACAAC

Prom *DOM34 L. fermentati* direct
TTTTTGACACTATTCAACATATTTGATATTCCCGTTTTCTGGCCCATCCTCTTGATGTAC
TTTATTATACTGTTCGCCTTGACTATGAGAAGACAAATACAGCATATGATCAAATACAGA
TATGTTCCTTTAGATATTGGCAAGAAAAGATACAATAATAAGAAATAAGAACAAAGTGCT
GGGGTATTTTTCGTGAATGACCACGTTTTCCTTGTCAAAGACCTCTGGAATATGCACATT
TAACTTAGCTTTAGATGTGCTTGAAGCACACATGAAAAGCTATTTAAGTAATCAAGCACA
TATTTTTTTAAATAGCTTGTGTTCTGTTGACTTCATGAATATAAGAGCTAGCTCTTGACG
ACGGATGTTATCTCGTAGCTTTGGCTTAATCCCCAGCTATTATCTTTTTAAACGACGCTA
ACTTAAGACTATATTCTTTCATTTTGTCATATAAATCAACGGACAACTACCGGAATTCAA
CTAACCACA

Prom *DOM34 L. waltii* direct
GAATTCAAGTTTTGGCACAATGCTGTCAGAGCCGTCATACTGAGCTCGTTCTTGACTCTT
TCCCGCATCTTCGATATTCCAGTCTTTTGGCCCATTCTCGTCCTGTACTTTATTGTACTA
TTTGCGCTCACGATGAAAAGACAGATTCAACATATGGTCAAATACCGCTACGTTCCCTTG
GATATCGGCAAGAAGAGATACTCCTCAAGGGACTAAACTAAGTTGGGGTCATTTCATAGA
AGTTACATCCCATAGTTCAT**GGGTGC**TATAATCATTTTCGTGACCAATGCAGGATAATAT
TACAGCTCCACAGACCTTTGTAGAAGGGGTGATGTTATATAGAATATCTTCAATTGGGAT
TCACTTCAAAATCTGATATATTCGTCTGTGAAGACCACTTTTGATGGGTGGGAGTTAACT
CATAAGAAAAATAGCAAAATAAGAATTTCTTTGGTTGGGTCATCTAGCATTAGCAGACAT
AGATCACTAGTTTGAAGAATC

Prom *DOM34 L. mirantina* direct
TTATCAGGAGATTACCAGAATTCAAGTTTTGGTACAATTCTGTGAGAGCAACGGTCGCTT
GTTTTGTGATGTCGTTCTTCACCTTCCTCGATTTGCCCGTATTCTGGCCCATATTAGTGT
TTTACTTCATTTTGCTCTTCTTACTGACAATGAGAAGACAAATCCAGCATATGGTGAAGT
ACAAATATGTTCCGCTCGATATTGGCAAGAAGAAGTATGGAAGTAAGCATTAAGCCTATT
TTAAAACGGGAACGAGTTGAAATCAAAGTCCTCAATGGCGTCTTTGAGTTAATGATCTAT
CTATTGTCGTCAAGGCGCAGCCAAGGATGGCCCACAGCGGGCACATGGATGCGAGACAGT
ATAGGTTAGCGCTTCCATGATTCGGAAGCCTTTAACGTCATTCCGTACGCTTGGATTAAG
TAGTCTAAAGGAAATTAGTTATCCTCATCGGTTTGCACTTTTAACATGTCAAAGTGAAAA
AGGCTAATAAA

Prom *DOM34 L. thermotolerans* direct

GTAAGGGCTGTCTGTCTTTGTTCAGTCCTAACGTTGTTCCGCGTTTTTGATATCCCAGTT
TTTTGGCCTATTTTGGTTGTATATTTTGTCTTGTTATTTGCGCTAACCATGAGGCGGCAG
ATTCAACACATGATTAAGTATAGGTACATCCCATTGGACATTGGCAAGAAGAGATACCGC
TCTAGAGACTGAATCTCTCGGCTTGAATTCCGATCTTAATTTCTATCACGTGCCATAAGG
TTTTGGTGATAGTCAATGCCCCTTGGGTTCAGCCACGTACCCGGCATCTACGTAGCGTTT
CTAGCAACCCCGATTTTGCAGCAATGGGCCCGCATATGATATGTTATTTCTCATATGTAC
AAGACTTTTGCTTGATTGTCAAAAGGCCTGACTAAATTCAATAGCAGTTTTTGAAATTTA
AGTTAACCGGTGTAAATTACATACAATTCCAACTCCAAAACTCATCAACAAATCTTCCCC
CTCA

Prom *DOM34 L. nothofagi* direct
GTTATTCCGAATCTTTGATATACCTGTATTCTGGCCCATTTTGGTCATGTATCTTATCCT
GCTCTTCGCCTTGACCATGAGAAGACAAATTCAGCACATGATAAAATACAAGTACACTCC
TTTAGACATTGGTAAGAAAAGGTACACTTCCAGGGACTAAAGGCCACTAAGGCAGTCAAA
AAGATCCGTTTCATAATTCAAACGAATGCCAATTATGTCCTACTTCACAATTTATCTCCC
ATTTAACGTGGGCCTTAAGTATTATTTTTCAATCCAAGTGCATAGCATGCTATCATTCAA
AAAGTCGAAGTATAAATCCTTTCCGTAGGTTCATTTAGTGACTATGAACATATTTTCTGT
TCGTTCGCGAAGACTGATCAAGGCCAGAGGCAATCTCCGTTCATGAAAAAAATAGATAAT
TTAATAGGATCAGTTCAAAACCTTAGTAACTAACTCTCTCACCAACGAACGTAGTCAAGC
ACATA

Prom *DOM34 L. kluyveri* reverse
TTTGTTATACCTTTCTTGTTAGCGGGAACAAAAGGATAAG
TAGGCGATAATTAAGCAGAAACTTCCTTAACAATAACCTACAGAGTTTTGCTTCTAGGGA
AGTGAAGAATTTTATTTTTTACTTTCAAATCTCACGTTTCTCGTCTTCTGTGACTAATAG
ATTCCATCAAAACTATTTTCCCCAGTCGAGTGTTTATATGATTCGGAAAAGAGATAGAAG
ATATATCAAATACCAGTGTACTTTTTCATCTTTTTATTTTCTCTCTCCTATTTTTTTATT
CGTTCGTCTATTTGTTGAATTTAATTATGCTCTTATTTATTTTAGAGGTGTTAGTTTGCT
TCTCTTCAGTAATGACAAGCACTAAATTACATGTACATAAATACCAGTGAGATAACTAAG
CTGATGTGCTCTTGATCAATAAGCACTATTCACTAGTTAATCTAGTTATTTGGGGCATTG
TATTTTCTCTTACCGACGTCAAGTGGAATGTATCTATATTTAATCATGTGTTGAATCTGC

Prom *DOM34 E. gossypii* direct
AGTTTTGGCACAATTCCATTAGGGCAACGCTGCTATCCCACTTCCTCGCAACATTTTCGG
TCTTTGATATCCCAGTTTACTGGCCTATATTGTTGATCTACTTTATTTTGCTGTTTGCCT
TGACCATGAGGAGACAAATCAACCATATGATTAAGTACAAGTACCTGCCGCTAGACATTG
GTAAGAAGAAGTACTCTCATAAATAAACCAAAGTCTGTTCAAGCGTTTGAGTATGTCCAA
TAAGGATACCGACCATTCAACT**ACACCC**TTACTGCAGCTCCTGACCATCTACGATTAACC
GTTAATGGGTATATTATATTTACGTATTCATTCAACTATAATTACTCGCCGTGAATATAC
TTAGATTGAGCAACCGCTATAACCTTGCATCCCATGCTGTGCATGTCACGTGCTGTGAGT
AACGGCTTGCTTGTCGAACGTTTAAAGAGCGTTAGGCGATAAAAAAAGCTCTCTGTAACG
CAGGCGCAGTGACAAGCAGCCCAATAACCTATT

Prom *DOM34 L. dasiensis* direct
TGAGGGCACTGATTTTGTGTTCTGCAATGACGCTTTTCCGTATTTTTGATGTCCCAGTGT
TTTGGCCAATTCTTGTAGTATATTTCATTCTGCTCTTTGCGTTGACCATGAGAAGGCAAA
TTCAGCATATGATAAAGTACAAGTACATCCCGCTAGATATTGGGAAAAAAAGATACAGTA
CCAAAGAGTAAGTAGCGGGTATCGAATTTTATTTAAATCACCGGCCGTCTAACAGAAGCA
AGGACGAAGTCTCAATCATATGCTTTTTGCTAAACTTCCCTTACGGATCTCATATATATG
AAAGTAAGAACCTGCTTGTTTCTCAAAGAACCCATTCCTTGCTGCAAAAGGTGTCTGATA
TAGGGATATGGGGTCAAGATAGTCTTGTCTCAAATTCTTTCGTCGGTGAAGACTGACGAG
TCTTGACCAAAGTGAAAAAAATCGTATCAGCCAAGAAGAGGCTACAAACGCAATCCTCAG
CTAGAAAAAATTC

Prom *DOM34 L. meyersii* direct
TCCATGAGAGCATTGGCGCTATGTTCTAGCTTGACTCTCTTCCGCATCTTTGATATCCCA
GTTTTCTGGCCCATTCTCGTTATGTACTTTATCCTACTGTTCGCCTTGACCATGAGAAGG
CAGATTCAGCACATGATAAAGTACAAGTACATTCCCCTTGATATCGGCAAGAAGAGATAC
GCTGCAAAAGAATGAGGACCTTACATTGAAGAGTATATTCACAAATTAGAAGTTGTGAAA
CCTGGAATCTGTGTCTACGCCCCAACTCAAAACCTTTAATTATATATATAGCTACAGCAA
TACACAATGGTCGTCCGTTTGATGTTGATGCTGATTATCTCCAGAACCTGACTTGGGCTT

TTCGTTCACGAAGATCCGCTAAAGCTTTTTTCAAACTGGAAGACTATTAATTTAAGCAAA
AAGATGTGTGAACCAAGAGTAAATGATAACTGACAACGCAGTAAAGAGGTCCTCAGGAAG
CGCGCA

Prom *DOM34 K. lactis* direct
CATCAGAGCAGCTGTCTTGTGTTTCTTCATGACTTTCTTCTCTGTATTTGACATTCCTGT
GTTCTGGCCTATTTTACTGATGTACTTCATCGTGCTTTTCGCGTTGACCATGAGAAGACA
GATAAATCATATGATTAAGTATAAATACGTGCCTCTCGATATTGGCAAGAAGAAGTACGC
TTCTAAATGAATGACTGAAAAGCCTTTAGCATACTATATATATATATAATTGTATATTAT
GTCATATTATAAGATATCGATAACAACTGCAAGTTATTTTACACATTAAGCCATACGAAC
AAACTTTTGTCAATGTAATTGTCCATGTAACTTACCTAGTTGCTTCTTCTCTTCGGTATC
TTCATGGCAAACCTTTTTTTTTCAAAGCTTAAAAATGAAATCTGACTATAAATCATGAAA
TAAAGAGGTATTGGCAAAAGCATACCTTATACACCTGACAGCAATTTATTGAAACAGCAC
TTAATACAGAACGTACCCAAAGAGGTGTCTCAAGTTAACGGGTAAAGAAGCA

Prom *DOM34 L. fantastica* direct
TAAGAAGACTGCCAGAATTCAAGTTTTGGTATAACTCCGCTAGAGCACTAGTTTTGTGCT
CGATTCTCACACTTTTCCGTATCTTTGACATTCCAGTCTTCTGGCCTATTCTCGTGATGT
ACTTTATCCTGTTATTCGCTTTGACCATGAGAAGGCAGATTCAGCACATGGTTAAGTACA
AGTACGTTCCACTCGATATCGGGAAGAAGAGATATACCCCCCGAGACTAAGTTTTATACT
ACCGAAGCAAGATTTTTAAGTCGCAATTGCGTTGGTTGTGCACAAGCATGCCATTACTAG
ACCTTTACTCTCATTACATAACAGAAATCATCTCACAATGATGAGAGGTGCTACATCCTC
TTCTTTATTATGGCCTATTCGATCACGGTTTTCGTTCACGAAGATCCTTCATACCTATTT
TTCAGAAACAAAAGAGACAATTATTTCGAGCAAAATCCTGTGCAGACACACTCAAATTTA
ACCCAAGGCCACCTAGCACTCCAATAAGGCCTGGC

Prom *DOM34 Z. rouxii* reverse
AATCCTTCAGCAGGTGTTTCTATAACTTGTTGTCATCTTTCAAATACTGAC
ACGGAGTAAACAATTATTAAACTACCTGGAAAGCTCCTTTCACCTCGAGAAGAATATCGT
ACGCAATTATACGCGGCTCGATAAGCCACATAAGACAAATACAAGTTTTTTAAATCTATA
TATGAGAAGATCGATGTAATTCCTTAAATCAATGATATTGAACTATATCTACTACGAATT
CTACAACTGTACAACTGTGCTAATGCATTCAATTTCTCCGCCGATCCACGAGGATGACCG
GAAACATTTTCTAACGAGTACATTTCATTTTGAACCATATTTCTTCTTGCCAATATCCAA
TGGGATATATCTGTATTTCAACATGTGTTGAATTTGTCTTTTCATGGTTAAAACGAAGAT
GATAATGAAGTAGACGAGTAGAATGGGCCAAAAGACTGGAACGTCTGTAATATTGAAAAG
TGTAGAGACCAAAGCTACTAAAGTAGCTCTGGCACTATAATGCCAGAATTTGAATTCTGG

Prom *DOM34 Z. bailii* direct
GAGAGCAGATGAATTTAAGCCATTTATCAGAAGGCTACCGGAATTCAAATTCTGGCATAT
CTCTGTCAAGGCTACTTTGATAGCATTGATTTCCACCATTTTCAATTTCACCGATATCCC
TGTATTCTGGCCAATTTTGCTCGTCTACTTCATCACTATTTTCTTTTTAACCATGAAAAG
ACAGATCCAGCATATGATAAAATACAAATATATTCCATTAGATATTGGGAAAAAGAAGTA
TACCAGTTCAAAATAGAGGCATATTTGGTGCATCATTGGTGGCTATTAGTTACATAACTT
GCGGTTTACGGTAAATATTAGATCGTACTTAACTCCATTACGAGGAAGCAAGTCTTTCCA
CGGAGATGTACAAGCCTTTTCATTGCGGATACCACGTATGTTCTATTGACTCCTCACCCA
TGCACATTATTTGGAGTATAATTGTATACGATTTATGTCGCTTTTAAAAGACTGGAATCT
TTGGGCTTATTGCGTCAAAGAGTACTGAAAGTACTTCCTCATTGACCAACTGTGGAAAT

Supplementary file S6: Sequences of the promoters from the *HBS1/SKI7* orthologues. The Aft2-like motifs are highlighted in red/orange. The Aft1-like motifs are highlighted in bright/dark green.

Seq prom *HBS1 Saccharomyces species* reverse

```
>Skud:YKR084Cu_YKR085Cd [1/Skud_11.321/-1/Skud_11.322/-1]
CTTGTCAGTCTATGGTGCTCAGAACAGACGTGTGCAGTCCTCTTCAAACTCCATTTTACGTCTCCTTCGAGCACT
TCAGTAGTTGTCTTCCATGTAATATGTCAAAATCATAGTAAAGAGGGCGTGTCCGTGCACTGTGATCAATATACG
AATACAAAATGC-TTATAGAGTAGTATTTACAGGATTGTTATCGATATCTATATAGCAGT
>Smik:YKR084Cu_YKR085Cd [1/Smik_11.347/-1/Smik_11.348/-1]
TTTGATTCTGTCTATAGCGTTCAAATCTGAAGCGTTTCACCCCGTCAAGGTTGCATATATGTCCTACTTAGGCAG
TTTAATATTGTCTTTTATGTAATATGCTTAATTCGCAGTAACTGGGAGTGTTTCCGTGAATTGTGATCAATATAC
GAATACAAAATGCCTTACAGAAGAGTATTTACATGATTGTTATT-ATATTTATATAGCATT
>Spar:YKR084Cu_YKR085Cd [1/Spar_11.375/-1/Spar_11.376/-1]
CTCTTCAGTCGATAATTTTCAAAGCAGAAGCAGTTTGGTTGCTTTCAAACTGTACTTGTACTCTCTTTAAGCAGC
TCAGTGGTTGACTTTCATGTAATATGTTAAAGTCACACTAATGGGGGTGTGTCCGTGCATTGTGATCAATATATG
AATACGAAGTGCTTATAGAATAGTATTTACACGATTGTTATT-ATATTTATATAGCATT
>Suva:YKR084Cu_YKR085Cd [1/Suva_11.320/-1/Suva_11.321/-1]
CTTATCAGTCTGTAGTGTTCAACACGGATGTTCCAGCTCCTCGAAAGCCTTTTCGAAACTTGCTTGAATAGCTTA
TTGATTAATTTTTATGTAGCATGCTATGTTGGCAATTATGGGGTGTGTCCGTGCATTGTGATCAGTATACGAATA
CGAATGCTTATAGAGTAATATTTACATGATTATTATTGATCCTTATATAGCATT
>Scer:YKR084Cu_YKR085Cd [1/Scer_11.350/-1/Scer_11.351/-1]
CTCGATAGTCAATAGTTGTCGAAGCAGAAGCCTTTGGGTGTCTCCAAGCTGTACTTAACTCTTTTTAAATTGCTT
ATTGGTTAGCTTTCATGTGGTATATTCAAGTCACATTAATGGGCGTCTCCGTGCATTGTGATCAATATATGAATA
CGAAGTACTTATAGAGTAGTATTTACACGATTGTTATTATATTTATATAGCAT
```

Seq prom *HBS1 Naumovozyma castellii* reverse

```
TTCACAGTAGTGGTAGTTTTTGCTTTAGT
TTTCTTGCCTTAGAACAAGTTTCCTCGATGAGATGAAATGTGTCTTTGCTTATATAAAAG
GCAGATTGATAGATCTTAATAAGGGTGCCGCTACTACGCTTATTAATTTTTCAAAGATTT
TCAGTGTCGTGTTTGGGTTTTTTGGGTGTGTCAGTAAATTTTATTTTATTAGACAATAAG
CAAGTTATAGAAGTATAGATAGCCAGAATATACAGCGATAATATATGGGTAATCAAGGTC
TGGCGGGTTCCTTAGCTGCAAACTGGAAGTCCTTGAAACTATTTTTCACGATGGGGGATT
GCAAGATGTCGATGAACCATCTCGTTATGTTCGGATGATGAGCTCTCCATTCATCACCCA
GCACGTTTCTCATCCCAAAGGTTAATGGTGCAAACGTCATTAGGTCAGCCAACGTAATAT
GAGTATCATTAATCAAGTAATGCTGCTTCTTTAAGATGTTTTCATAAACGTCGAACATCG
```

Seq prom *HBS1 C. nivariensis* reverse

```
TTAATTAAATTGCTATCCTTTTTGTGTGGTATTAATTTGATACTTTGCTTGAACAGGAAG
AATGTTTTCAAATCTACCAGTTGATAAGCTTCCATTGGTATTTATATTCGAGAAGAGGAA
AGTTCATCAAAATTTTTCAGCTTCAAAAAAAAAGTTACATTGCTTCTACGATACTTTCTT
AGCCAAAATGGGTGTGTGAATGTAAAGACACCCAAGAATAACCTCAAGCACCAGCTCATC
GCATTGAATTTCATTTCATCTTTCTCTCTTTAAAATGCAATGTTCAGCTGATATCAGCTC
CATTATGGCCGAATTATCATCATATGAACATGGTTTCTGTTATCAATTTTATTTACATGG
TTATATATATGGGGATATATTACTACTATATATTTATGCTCTGTACCACAGTTCTTGTCT
CTTCTTTCTATCCTCTCTGGCAATAGCTCTCTTTGGGTGCCATTTTGATTTAATTGCAGA
CAGTCTGGAGTTCATTTCTTGTTTGTGGGCTTGTGGAGCATCGCTTACAATGGATATAAA
```

Seq prom *HBS1 C. glabrata* reverse

```
GTTGTTAA
TCAATTGTCTGGTTGGTTCTATTCAACTTTATCATGAATTGGCAATTGTGGGTATTACTC
TTATATAATTATACAGTGAATGATGTTAGGACACCCCCATCCACAGAAACCTGATGCCTA
AACTATCTTTGATGCTACGATTTTTGCCATCTCGAGTGATAATATCGTGATAACAATAAT
```

TTTAT**GGGTGT**ATTATGGATTTCCTAAAGTCAACAG**ACACCC**CCTCCAGTCTAATAGGAG
TAGCATTTCAGGTTGAAGTTTGGACTAGATTTCTGAAACTAGCTTACATGTATCGAGGTT
TTCATGAGAGCTATTTAACCTTTACATTTCCCAACCAAATGATAATCGTTTGGTGATTTG
ATATTTAATGAAAAGTATGTACAAAATTTTGTTAATTTTGGGATATATAATCTTGTTACG
TCCTTGATTGGCAATGTTATTTACAAGTATTGTCTTTTTGATATGTTGTCGTGATTGTAT

Seq prom *HBS1 N. bracarensis* reverse

CCTCTTTTAGTTAGCGTCTTGTCCT
GCTTGACACTAATAGAATCTCTTTTCAATTGATGTCCTGTAGAAACTCTTGGCCCTTTAT
AGGCCATCGAGCAGTTTTGCTTATATGTCATCAAATTTTAACAATTTTTTTTTCAGTTAA
GGTTCTACGGTATTTTATGAGAGGAACCTGATTGTTATTAATAATTCTATTAAAGA**GCAC**
CCATATTTCAAATAATGCTAGCAATTAACAATGCCATATTCAATCAATCGACTTATACGA
ACCGATCTACAGTTATATTAATTTCACTTTATTCTAGAAATAATTTAATTAGTAATCAGC
TTAGTGAACACCTATATTTACATGAATTTTAAATAAAGAAAAAAAAGAGAACTATTGTTG
TACATATTATTTTCAAGGATATTAGTTTGTTTAATTTTTTTTAATGGAATTTATTTTAGA
ATAAATTATGCTCTGTACCACAGTTCCTGTCTTTTCTTTCTGTCTTCTCTAGCGATAGCC

Seq prom *HBS1 N. delphensis* direct

GGGCATAAACCTGATAAATAACCCGCATATAATTTTCCATGTAAATAATGAAAATAATAA
AAAAAGACATATACATATACCTACATATTATAATATCTGCTTTCCTGGTCCTAGATTGAA
AAGGGTCGATGCCTCCGCCTGTGGATGAGGGCAGAGGCGGGGCAATGCATCCCAAATCGT
TCTTAGAGCAATACGTCGCCATAGCAGAGGATGGAAAGGCTTATGTATAGCAGTGATCCG
TGACATTTAAAAAT**GGGTGT**CTCTTATTCAGTTCAG**ACACCC**ATTTTCTCTAAGAAAATG
CCGTAGGGACGAGGCCAAAAAACAACTACAAAACGGTTAGAAGCCGAACCAATATTTGAA
CAATTTCAACGTCTTCGAATAATTATTATAAAATAAGGATTGGTATCGCAAACAGCACTC
ATTTTGTCAATCTCCTTGGCAAGAAACACAGCAGAGTAAGCTAATTATTCTTTAGGATAG
AGAAAAAAAAAACCGCTAACAAAAAGA

Seq prom *HBS1 T. blattae* direct

ATTCTTAAGAACAATTTTCATGTATATAAAAATCTTCGGATATGGTTCTTAGGTGTTCAA
GATATTTATATAATTAACAACAACTATAGTACTACTAGTCACGACAGTCTTTGTAAATAA
GTATATAAATACGTTTATACCAAATTAAATTAATTAATTCATATAAAATTATATCAAGTA
CAATCTGAGAAGCTCAATTGAAAAAAACAATATTATTTTTTTGTGTTGTCATAAAACGTG
GCTTATTCTACAGATAAAGAACCAGCACATTATGAATAGTTATTTTTTTTCTATCTAAGT
AGAATTAACATGCCATACAATGCACTCTTCGTCATCGCTTTTAGAGATTTTTCTATCTTG
CAGATTTT**ACACCC**AGGGACCGAATAATGAATCTTTTTGCACCTATGAAACGTAAGAAAA
TTATTATAAAATCTTATCTAGCGTATAACATTTTCTAAAAAAAAATCAATCCTAATTTTC
AAAATGCTTAATATAGCTACGTAATACAAGTTTACTATTAGCAAAAGTTCTATAAAGAAA
G

Seq prom *HBS1/SKI7 L. thermotolerans* reverse

CTTTCAGTCGGTTG
TTGACGAGCCTTATTTCGTTATTGTAGCTTCTTTACTATCTTTGCTCAAAATACAACTAT
TTTCAAGAAGCTGCAGTTTATACACATTTTTCAGCTACGTTGCTACTATAAGCGTGAGTG
TCAAGAAACATACAATGGGAAGCTAACAGCCCTGTTTTTACGTTCATGATATCATGAAGA
TGCGAAGTACGAGAAAGGCACGTCCCAGCCAAGTTTCGCAGCACTCTTGCCGATGACCTT
GAGATTTGGCCAGTCAACGAATGCGTACAACATGATTTAAGGTTAAATCAGCTCTTTGTG
AGCAAACATTCAAAAGTTCTTCAGCTTCACTACAGGGCTTCGCTGGTATTTGTAACTTTG
TCGCTACACAACTGATATTTTCTCAATGGGTGGGTTGTAGTAGTAATTTTCGCCCGGGCG
CATCCTGACGTCGATATATACCTTCAATAGGGCCTGGTTCAGCCACGTTTAACGAAACAC

Seq prom *HBS1 C. castellii* reverse

ATCTGTGTTGATCTTTTAGAT
CAGTACGTTGCGAAGCTCTTCTGCTTTTTGCATAAATGAAGAGTAATTAGAGATATATGA
TATATAGAATTTTGGTTCTCACAGACGCTGAATATGTACAGGTATTATATGTAGTCAACT
CAAGATATATGAAGACTGAGAATGCGAGTGATCAAATAAGTTGCGCATTCATTTGATGGA
TTTGCATTTAGAACTTCGATCCAGGATCATGGCAAGAACATAATATGGTATATCGAGCGA
ACCAGGGAAAAAAAAAACGCTAAAAGAATTCATGAAAAAGAACTTTAGACCCTATCCCAA
AAGTGATCAACAAACCATGATGTTAGGCGATTCAAGGATGTTGAATCAACAACCAGAGGA
GTAATACTCGCTCTTAAAGACAACAAGTTTAAATCAATACGTAAAAATGTCCCGATGACG
AATGTTTGATAGAAACGCGTCGGCATGACGCGTTGATTGGGAAGATATTTCATGAAGACC

Seq prom *HBS1 N. bacillisporus* direct

CCACCTGTATTGGTCAGCAAATCTACAATTGAGGGTAAGACTTACCATTTGAAGCCCGAA
CAAATACAAGAAATTATCGATTTGAGGACAAAAGATCCTGATACATATACCAGAAAGAAA
TTAGCATTGATGTACAACGTATCCCCACTGTTTATTTCCACTGTGGCCAGCACTACGAAG
GACAGGAAACAACAAATGCAGGAGAGATTAGAGGTGATTAAAAGCCAATGGAATCATCGT
CGCCTGGACGCTAGAAATGATAGAAAAAAAAGAAAAGCTCTATGGTATCGAGCATAACCA
CGTTGATATACCATTTATCTTGTTTATATGTAAATTCTTTACCTACTTTTTTCTACACTC
ATGTATATATATAATTTCATCTTTTAGTATCGTTCATAATCAATCAATCATAAAGCGAAT
TAGTAATCCTCTTCAAAAGTTTTAAAAACAAACAGTAGTTGAAGGATACACACCATTAAG
ACGATAGATAGACAAAACTAACTGTTATCACG

Seq prom *HBS1/SKI7 Z. balii* direct

TTTGCCATAATACTGCTTTCACTCATATTCATGTATTCCTCGAGCACTACATCACCTCGA
AATAATTTACACCGTTGACCAGCAAATGCCCGTTCATTTGTTAAATCTGAGGGCAGCACA
TTGTAGAGCACTCCCTGGTGAAATAGAGTACTCCTAGCTAAGTACACCGACCCTTTGGAC
GGCACCTCCGCCTCCTGATTACTCATTGACGTAGTTACAGCCAGCATCGCATCGAACTCT
CTAGTCACTTGATAATTCTAAAAAAAACCCGTAATCTTTTGTAACAGCTTCCACCCATCG
ATTAATTTACGACTAAAAGCTACAACGAGTTGGTTTGGTTATCGTGCCGTTTTTGACTTC
ACCGTTAGTCAATCAATGTAGTCATGTAAGCGTTTTTTTTTNNNNNNNNNAAAAAGAACA
CCGATTAGACACTGCAAACTTCACTTACCAGATAAAGCTATCGGTAACTTATTTATTAAA
CTTACTCTATATATTTGGAGTCACG

Seq prom *HBS1/SKI7 Z. rouxii* reverse

ACTGGTCTAATGATACTTCTTACCACTTACT
GTATACCTTATTGAATCGTTTTGTAGTGATTATCTTTTGCCCCAGTGCTCTTTTTTTTTT
TCATTTTTTTTCCGACGGGCATGACAAGCATCGCTTCCAAAAGGTCGCTCGGTTAACTTG
ACTCATTATGAAGTCAGAAATCTTTGATAATCACCAACATACAAAACTTTAGAACACATT
TTATAAGTCATTTGCTCGATTCTTGACTAAATTTTTAGCGTCCTACGTCTTGTTGAAGTC
GATGAATTTCGATGTTAGCCGTTAGTTCGATGAGTAGTAGTCGAGAAACTGCCACCACCA
AGAGTTCGTCACTAATACCGTCAACTTTCATTTTGGCCAGATCGAGTAGATATCACAAGT
GTGCTTTGTTCAACGTGCTGCCGTCTGATCTTACAAATGAAAGTGCGTTTGCCGGTGAAA
AGTGTAGATTATTTTATGGAGATGTAGTTCTTGATGAATATATTGAGAACGGCAGTAGTA

Seq prom *HBS1/SKI7 L. dasiensis* direct
GCAAGATGAGCATAGATAAAAGCCAAATCGTACACAGTGATAACAATACTCATTTCTTCT
TTATATAGTTTGACAGAGAATTTAGGAACGCCACTAGTCAGAATCCAAGTATGGTCTCTT
TTCAAGCAACTGTGAGCTTCTAAACTGTGTGTTTAATGATCATTTAGTTAACTAGCTAAA
TGTTCAAGCTACATCACTACGGACGACTATGATGCTTTCATCATTAGTGTTAGAGAAAGG
CCACCGGGTAAGAGAATCAGCACTTTACT**GGGTGT**CAGGCGTTTATTTTACTGTCCACTA
TAAACAACTACTGTAAGTTCACTTACTCATAGAATGTTTTGCGTTGACTACCAAATTTAC
TATGACTACCGTGTTTTTTACTTGCAATCTCGCAGTGCAATAATGAAGAATTAATTTTAA
TGATTCTACCCTACCGACTTGAAAGTCTCTTTAACAGACATAAGTCTGCAACTCCAATAC

GGTTTATCCTGTACTTGCCACA

Seq prom *HBS1/SKI7 L. waltii* reverse
ATTCGGTGATTGATGTACAATTGGTTAGGTGGTAGTTGCAATTG
CAATTGACCTTATGAACTTGAAATGCTTGACCTATGAAGTAGCATTATACACGAAGATTG
CTCACGATAAGTTACCGTTGGCTCTACCAGATATCCCGAAGGTGATATAAGTACTACTGG
TGCTATGTTTGTGTCTTGGAATTGGTAGCTCGAGCTCTTTCGCGGAGTGCTGACTGATTT
TTTCAGTTTGAAGATCTTATATTACATTTCAATAGTGCTTCTTTTATGACAGTATTCCAC
AATCGTTTGAGACGCCAGTTTGCTTAATCTTAGTCCCAATGTTTTAGTTGTATCAGTAGA
GCTGCTCAGGGAGTACTAAATATATAGTATTGAGATACCGAATTTGTCCTTTGACAAATT
ATGGGCCTGTGTCATTTGTATCAAAAGGCTCCGAAAGCAGCTCAATGACAATATATAGAC
ATTATACAGTTCGAGCTTTACCAGGTCGAGGTGTAGGAACGAGTCTACCTCTCTCCCGCA

Seq prom *HBS1/SKI7 L. nothofagi* reverse
CTTACAAATGGGTAAT
GTTTAGCTCTTAGTGCTCTTCTTCTTTTCGTAAATGAGAAGTTGGAAGGAACTTAAGTCG
ATTTTGCCATTTGACAATACTGCAACTGCTGTTGGAAAATCAAAAAAATCAAATATTTAC
GATTTTATCCTGGCTTATAGAGCCTCACAAGTGAGCAATTGGTAGCGGAAACCGATAGGC
TAGAGGCCTGTCAATGCTGGCATAAAAAGAGGTCACAAGTTCCCAACTTACTATTTAACT
TGCTATTATCACTATCGACGAAGTGTAATGCAGGCAAGATTATGTCTACAACGGTGAGTT
GAGGACTTTTAGGAAGTACGTGCTAACTTTACTGCTCAGCTGCTATAATATTAGCAATTC
GATTTTTCATTCCATCGAATTATTACAGCTGCGCGCTTAATTATGACCTCGTTTCCCACG
AATAATTATTATTCCTGATCTTTTCATCCGAAAGAATCATTGCCTATAGGTGAATAACGA

Seq prom *HBS1/SKI7 L. fermentati* direct
AGCTCTGACACGCACATTTTACCTTGAAATAGTCTACATTGGGGTTCCCACGTCAGCGAA
GATGACGGTGGTAAAACATTATAAAGCATTACAGTGTCAAAAAGCTGTTTCCTTGAAACC
TTATCGTTATCCATAAAACTGAAAGGCTCTGGAAGGTTGCTAATCGTCTTTGTTCGACTG
GACGGCAGTGATTAAATTTGAAATGTTTATGTTAACTCCTTTCCACGATAGTTCCATGAT
TGGTGTATGGGTGACTTGATAACAGACAACTCAGCTATTAGAAGACATCACGGTACAGTA
TGCACAAGATGTGAAGGATAGTGAGGACGATATTCGTTTGGGCAAAACCCCGTTATTAGG
TATTCTTCGAAACAGGTAATTTAGGTATCCTCTGATAGACATGTGTTCTTTGTTTTTTTC
GAAAGTTTGCCATGATTAAAAACGAAATTTTTACAAGCAATATCGGGTTATTCAATTGAA
GTTACTTTTGAAAGCCAAA

Seq prom *HBS1/SKI7 K. lactis* direct
ATAAGCTTCCCCTTCCATAGCAAATGCTGGGGTTCCCACGCTCTTGCAATGCTTCGCGGT
ACGATATTATACAAAACACAACTCTGTATCACTCGTTGATGCGTTGTCAGCACATTCTCC
GGAACTGTCATCCGAAAACGTATCGGCTATATCACTTCTGTTCTCTTCAAAATATTACCT
AGGAAATTCAACTCGTTCACCTGTTAAATCACCTACCGGGAATTACCTTCTTGACCAAAA
AAAGAATGTACTAATGGACAAACTGCACTGTTTACAACTCACTTCATGCTATCGATAACA
ACTCATCTCATCTCATCGATAAAGAGATTGCATGTCGACGCAGCAACTGATAAAGAAATT
TTTAAAGTCACGTGACAAGTATCAACAGTTTCTGAAAAAATCTGAAAATCTCAATTACAA
AAGGTTTCTAATCGCAAGCAATTCTCTTGTTATTCGGTTCAACCTGCATTTAAACTCGAA
TTATACCGTCTATTGCCTGATTCTCTGACTAAGCGAAGCAGTTTATT

Seq prom *HBS1/SKI7 L. meyersii* direct
GCTTACGATGGCACTGGCAGATATCGCATTAGTAAACTGTTGATGAAGAAAGTGTCGGTG
TCAATGACAAACAAGTCACTTTGAACGAGGGTAGTTACTGAATTGCGTCCGTAATTGGAT
CTTTGAACTCGAAGATGTTGTAAACTGTTGTATTCCGCCATCCAAGTTGCAGGCGAGCTA
CCACAGTTTTAAGAAGCTCTTAGAAATCTTTTTGTTGGCTTCAATTACTAATTTGCACTA
CTCTGTTTCAAGACAAAACCCAACATAACATGGCGTTTTCTTGTACGCGATGATCGATGA
GCCCAAAATTTGAAGGTAACTGTAAACAAAATAGTTGTACACGCATGGGACTTGACATTT
AATGTCACCGGCAAGCTTAAAGTTTGCACGTGATGTCACATGCTCTTCAATTTTTGCTTA
CATAATCGCTTTTCCAATTAAATGAAAGGTAAAAGTGCCACTCAACTCACATTAACCATC
CTCCAGCAACACTTTCTTTCAACAATAAACCCCATTACATCCAGGTAGTACA

Seq prom *HBS1/SKI7 L. fantastica* reverse
TGTCGAACCTTATCACTTGTTGTCAATGGTGTTGGTGTTATGCGATCTACTGGAAAGCATAG
TTGTCAAGAATATCTAATTAATTGTGGTCGTTATTTGCGATTACCGTACAAAATGCATAT

TTTGATCACGTGTACCTCAATCGATGTCACTATCATTTTTACGTTAGTAGCGGATACCAG
GATTGATTCTCAGATTAAAGGGTTCCTTACAAAGTGATTGCGTTCTTCTTGGATACTTTT
TCTTTTGTTAGATTTTAATAAGTCTTTTTGATATGTATTTTGGAACTAATCGAGCCAATA
TGCAATACTTATAGAGCTTCTTACGTATTATTTTGAAACTGTTTGAAATGCGACATTAGC
ATTCAATCTCCCAGAAACGTGAGCACATTAAGTGAAGTAAACTGACGAATTGACTACTAC
TCAACGAAACATTTGCAGTTGCAATTAGTGATGACAGACAATTCAACGTGATAGTACTTA
TCAATAAGTGAGATATATTTTCGTACCATGTGATATTCAAAAAAAAAATAGAGCGACTGC

Seq prom *HBS1/SKI7 L. mirantina* reverse
TTCATGCATAAGTTCGTACTCGTCGTCATTTAAATAATCGTCG
AACTCGGCCTCATCCTGGAACTCAGGAACGTCGTTTTCATAATCAACATCTGCATCATCA
TCATAAGGCCCTGACATAATAGCTTCTAAGAGGTTTGAAGTTCGTTAAGTATTGGTGATA
ATTGGTGATTCTTAATTTAAGAACTTCATTTTTCAAAAATCTCTATAGTGATGAACTACT
TTTGCCGCAAGAGAAATGATGATGACATGAATAAACAAGGCATTTTAAGCAGAGAGGCAG
TGTAATCGAATACTCTAAAAATTTCAAGCCTCCAGTAAAATGCTGTAAACACCTTTCAGC
GCTTCAAAATGTCAACCAAAAGATGAGATACGGTGCCGTTAAGAAGCCGACCAGTCAACT
CCATCATACTTCGAATGCTTTACTAATTACAGGACTACCAGATCATATATACCGAGACTT
TCAAGCTCTGTACCATAGTTCTTTTCTCTTCTTACGGTCGTCTCTCGCCAGGGTTCGTTT

Seq prom *HBS1/SKI7 L. cidri* reverse
CACGACGTTTGGTACTTTTTGGGCTTGAAGTTGAAAGT
GTTGAAGATTTGCTTTGGAAAAATCATTTTTTATTATATATCATGTTAAGAAAAGAGACG
AAAATTATCTCTACGCATATTTGACGTTAAATTCGTATAAGCCGTTGCGATTTAAAACAA
GAGCTTGGTATGCTGAAAATGTAGAGTCCATACGGATAGCTTATAACTATTCTTGCATCC
ATTAGTTCCGTCTTGATTAAAACTATTGACTAATCTAGCATATCAATTCACCCACACACC
ACTGCGGAGATACCGTCAACATAATTAATCACTGGCTGGATCGAACCTTCACGATATCGA
AACTTCAAAGAGATAGACAGTATCATTAGTGACAGTATAAAGTGGACAGCATAACGGCTT
ATGTCAGTAGGAAAAATTTCACGAAAGCAGTTATTTGGCGCTGTATCGCTGTATAACGTC
TTACCACCGTCGTCTTCGTTGACATGGGAACCTCAATGCAAGTTGTTCGAAGGAATAATG

Seq prom *HBS1/SKI7 L. kluyveri* direct
CATCTATCCTTCTTTTCTATTGTTTCGTTAAGCTGACCCAAGCTTTTAATAACTTGTGTA
TTACAGAACTTAGCCTCGGTCTTATTCCACTGAAAATTTAGCTGGTAATGCGGCATGTCA
TCATCAGACCTGCTAGGTATACTGTTACCACAGGTTATTAATTCCTCCAAGGAAAGTTGA
CCCTCGAATAGTTTGCATTGCGGTTCCCACGACAACGATGAAGACGGTGGTAGAACGTTG
TACAAGGACGCTGTATGAAATACCTTTTTAAGCGATACTATACTGAGAGACATACCAAGA
TACTGTAACTAAAGCTAAGGCTCCTCCAATTTGAAAAATAAATAAATAAATTGATTAGGA
CAATCCAGCGATAGTTTTCTATCATGTTTAGAAAAAGAGCATGAGTCAAGTTGAAGAAAA
AATTCATCGGAAAAATGTAGATTTCGCGGCACGAAAGAAAAATGGTTTTGAAATTTGAAG
TGAGCTTCCTTTAGTTTTGACGGTGGCAAAAAGCAAACGGATATACAAAC

Seq prom *HBS1/SKI7 E. gossypii* reverse
CTCAACTGGCTAGCACTGCACGACAGCGGTATGGGCTTTGTGCTTATCCCTTAGTCT
AGGGAACGTCTGGATGAAAAGGCCTAGAATGTGCGAACAGGTTGAAAAAGAAAGGCTGTC
AATCATCTGATTCGATGCGGCGCCTACTTCGCTATTTTAGTTTGGTGCAAGTCATCCGGG
TTTACGCTTAGGTGGCTATACGCAGGCAGGCTTATAGCGGGCCACGGCTGTGCTGGGGAT
GGCTGCAGATAGTTATATAGTAACACACAGGCGGAATATCATGCAGTGCCAGCTGTTCAA
CGTTGTTCCTACGCAAAAACGTAAACGGGAGTCGCAGCAGCGGCTTTGGGCGGGAAAAGC
CGTGTTCGAGGAGCTCATGCTCCCGGAAATGGGGCAATGGACGCCGACGTACCAGCTGAA
GTTCGAGTATGAGAGCACGCCGCATGTGTTCTGCCGGACACGAATAATGCGGAGGCTGAG
AGACGTGAAACGGGTTCTGCAACGGCAGCCGGACGGAGCATTCCACGTGCTGGCACGGGC

Seq prom *HBS1 K. africana* direct
ACTATTGTTTCTACCAAAGGATGATATTAGACGCAGATACATCGATCAAATCAGGGCACATTCTAAAGAC
AAGGTACAGAGTCAGGTGGCACCGGCAGTACTTTGCAGCAAGAGCACTATGAGAGGTCATAAGACTTACC

ATTTGAAACCTGAGCAGGTACAGGAGATCATCAAGCTACGTAAGGAAAACCCCACTATCTACACGAGGAA
AACTCTGGCGAAGAAGTACGATGTGTCGCCTTTGTTCATCTCGATTGTGTCAAGTGTCTCACCGGAGAGA
TCCAAGGAAATGGATAGAAGACTGCAAACTATCAAATCAAGATGGCATGAACAGAGAGCTGTTGCCAGAG
AAGATCGAAAGAAAAGAAAACAGATGTGGTACATAGGATGAATGGCTCTTGTTAGAGCCACTGACTACAG
TTAAGTCCATTGTTTC**GCACCC**AATATCGGCTCATC**ACACCC**TACAAATTACTAATCGTAAATAATTTCA
TAAGCTTCATCTTGTATATAAGAAAAATTAAGTCTCTGCCGAGTTTTAGGTGAAAAATTTACAGCATCGC
TATATTCAGGAACAACATCATCACTAGGAATAT**ACACCC**AAGCAACGCTTAAAG

Seq prom *SKI7 Saccharomyces species* reverse
>Suva:YOR076Cu_YOR077Wu [1/Suva_8.125/-1/Suva_8.126/1]
GATTGATGGTAATATAACTTTTATGGACAGCCTCCTCTGACGGACTTTGGGAGATACCTAAGGTTCAATATTTCA
TTTAGTTACGAAGTAACTAATTATACTTTATAGCACACACTCGTCCGGGTCATATAAATGTTAACCCGCAGCTTG
AAAATTGAAAAATGAGTGCCACGAGCAGAAGATAATCTCAAATGAAAAAGCTCGGTGTTAACATTATAAAGCTCA
TCAATACACAACTTGCGAAAAGGTCTCAGTCTAAAGCGGCTCAGCAGGACAAATCACCAAC
>Scer:YOR076Cu_YOR077Wu [1/Scer_15.287/-1/Scer_15.288/1]
GTCAGGTGGTACTGTAAGTTTCGAAGACCACCTCCTCGTACGTGTATTGTAGAAGGGTCTCTAGGTTTATACCTC
CAATCTGTTATAGTACATATTATAGTACACCAATGTAAATCTGGTCCGGGTTACACAACACTTTGTCCTGTACTT
TGAAAACTGGAAAAACTCCGCTAGTTGAAATTAATATCAAATGGAAAAGTCAGTATCATCATTCTTTTCTTGACA
AGTCCTAAAAAGAGCGAAAACACAGGGTTGTTTGATTGTAGAAAATCACAGCG
>Skud:YOR076Cu_YOR077Wu [1/Skud_15.241/-1/Skud_15.242/1]
GTCTACTTGTACTATTAGTTTCAGAGACAGCCTTTTCTCATGTATGATAGAAATCTTCAAAGGTCAATTCTTCTA
CCCAGTTACGTAGTAAGTAATAACACACTTAAATATGCCTTTGTCCGGGTCATACGGCACGTTCACCCGCAGCTT
GGAAAATTTGGAAAAAGAAATCCGCCAGCTGAAGTAAATAATCAAATGAGAAAGCCCATTGTTGTCACTTTTTTC
TGAAGCTCACAGCACATAATCTTCCAGAAAACGCATATAAAGAGATTTCCAGCGGTAATAAAACA
>Smik:YOR076Cu_YOR077Wu [1/Smik_15.254/-1/Smik_15.255/1]
ACTTCCAGTCAGTTATAGTACACATTAATACATTCTTGTCCGGGTCATGCGACAAGCTCAAACCCGCAAACTTGA
AAATCGTGAAAAGAGTCTTATTAGTTGAAGTTCAGTACCAGATGGGAGGACTAAATATTATCATTTTTCTTGGGG
AATAAATCAATGAGAGGAGTCAAAACAGCTTCTATAATATCACAGAA
>Spar:YOR076Cu_YOR077Wu [1/Spar_15.283/-1/Spar_15.284/1]
GTCCGGTGATACTATGAGTTTCAAATACCACTTTCTCACACGTACAATGTAGAGGGCTCTAGAGATTCATACCGT
CACTCAGTTATATTATAATACTTATACACTCCGGTCCGGGTTATACAATACTTTGACGTGGAATTGAAAACTGGA
AAAAAACTCCACTAGTTGAAGTTAGTATCAAATGAAGAGGCTCATTATCGTCATTCTTTCTTCTTGAAACAATCT
CAAGAGGAGCCAAAGCAAAAGGTTGCTTAATATATAATATCACGGCA

Seq prom *SKI7 N. bacillisporus* reverse
CAAATATAATTATGGTTGGAGT
CCTTTAGTATTGTACCTTTGCAGTATCTGTGTTCTCTCATGCTAGTCTATTTTAGCACCA
TTTTTTTTTTCACTTCTTTCATTAAATTATTATCAATAATCTAGCAACTTCTTCAAACAA
TTAGATAAACCAGCA**ACACCC**CTCTCCGGTTCTCAGAGTTTGGATAATATAAATATGCAG
GGTTCTGATTTTGGGACAGCTAAGCATTTGAATAAACAGCTGAAATTGCGTGGATTGCAA
AAATTAAGGTTCTATTGTCAGATATGCCATAAGCAATGTCGAGACCAGAACGGTTTTAAC
TCCCACATTAAGTCGTCTTCACATCTCCAGAAGATTGAAAATGTCACTGATGATGACATC
GATCGATTCACCCAGGAATTTGAAGCTAGTTTCTTAAAACTTCTGAGAATAGCACATGGA
GAGAAGACCATAGCGGCAAACAAGTTCTACAATGAGTTTATACAGGACAAAGATCACATA

Seq prom *SKI7 T. blattae* reverse
TTATGATTCTTGTTATCGATAATTGTTTAAGGCTTTTCGTCT
TAAACGTATTAATTGCCTTTTATAATTTAGGCTAATGACCTCTAGATATAAGCTTTATTT
ACACCTATTAGTAAATAGCAAGTTTTGGGAAAGAATTTAAGATTTATACTAAAGCGAATG
TTAAAAAAAAAGATATCATGAAAAATTTTTTTGAAAAAA**ACACCC**TAAAAATATTAGATA
TCAGATAATTGTATTACTAAATATTCTTTTTCATTCAAATAGTTTTAATGGATAGTTATT
CTGATGATTTGAGCTTACAAACGCTGATCCAAGAGCATACAAGATCAAATGTTACAGTTA
ATTTTGTCAAAACCCTCCGCCGCTTAGCACAAAATAATAAGGATAGCCAAGAGAACTTCC
TAAAAGGTTGTCAAGCATTGGGGAAATTTTTTAATCAAGATAAATTCTTAATTGATCTTC
ATTTTAAGCTTATCAGGAATGAATCATCTTCCAGAATTCATGGGTCTACAGGGCAGACCA

Seq prom *SKI7 N. castellii* direct
TTGTAAAACTTATTAGCCTCAATTTTCTTTTCTCCATGATTAAGTTTTAACAACCTCAAG
AAGTTCCTCTCAAACTCAGTAGTATAGTTATTAATATCCTCAGCAGTAATTTGTGAAATT
TTTTGTAAGTGAGATGGTGCCTTGATATGGGACCTGAACCCATTCTCATCTCTACATTGC

TTGTGACAAACCTGGCAATAGAACTTCAGTTTTTGAAGGCCTTTGGCCTTCATCTGTTTA
CTGAGAAACTTTGCAGTTCCAAACTCTGCCTTAGCCATCCTATACTTCCTCTTTTTCTCT
CCTTTTATTCATTTATTCTTGATCTATTTACCATCAAGTAAGTTTCTTAATGCTTGCTG
CGGGAAAACGCACTGCACTGAAACTTTTTCCTCAATGTGTTATAATAAGTAATGGAAGAG
TCTCAAAACATTTAATACCAGGACACAGAATACATATAGGCTAGTCTGATATACCTGCAT
CCATTGGCAATAACATA

Seq prom *SKI7 N. bracarensis* direct
TTTAGTTTTTGTAAGCCTCTTGCCTTAAGCTGCTTGTTCATATATTTAGCACTGGTAAAG
TCCCCGGACATCTTTTATCACTGGTTCTTTTTTCTTTCTGCTTCCTCTATTTTATTTATT
ATTATGTTGTTCCTTATAAGAAAATATAAAAGTTCAAATATACCGTCTTGTATTGGAAGT
GGATATAACTATTTTATAGTAATTCATATGGAGACATTCATCACAAAGTAAAGTGTATCT
TTAAGCTTGATCTTAACTTAGTTTTTAGATTTAATACTAATTTATTTATTTTTTTAATTA
ACGCTTTGCGCACAAAGCTAAAGGGCTGAGTAGCAATAGTACTAAATTAAATTAAAAAAA
ATATAAAAAGCTTAATTAGTTTGAATGTGGCAAAGATACTGCGAGAGTACAATATAGGAC
CCATATCAATAAGAATCAACCTAGCTTTACTTTTCTTCGAAGGACTCATAAGGCCAAAAC
CCAA

Seq prom *SKI7 N. delphensis* reverse
ACCAACTATGTAATATGCTTTTAAATACAAAATTAT
TTTCTTATTGATATTTATTTTCCTGATGATTTTCTTTTAAACGACATCTTGAAACCCTTG
GCTTGATCATTTCACTTAATTTCGAGGTCGCTTTTAGAGTGGCACCTCTGCGGTTTTACA
GACTTGTTCTAATTTCTTGGATTCGACCCATGTTGTAGAAAGGGAACCAATACTTCGATT
TTTCTGCTATTGATAAACACTTTGAAGAATTTAAACATCGGGTTTTAAGGTTTATGTGTT
TTATTAACATTTCTTCACACCAGCAAGCTTACAGGATATAACATAAGACAATCAATAAGA
AAGAAATGGCTGATGATTTCACAAGTGCGAAATATATGAACAAGCAACTGAAAGCTCATG
GACTACAAAAACTGAAGTTTTACTGCCAAATATGCCATAAACAATGTAGAGATTCAAATG
GTTTTAAGTCTCATATCAAGTCTCCTTCTCATTTAAGGAATATTGCATCGGTTACCGCCA

Seq prom *SKI7 C. glabrata* reverse
ATCCTGGTTTCATAAAACTTCAAGACTAGCACT
CAAAAATCTTTGTCCCGCTTCAACTTTCAAGTGGCCAATTTAAGGTTACTAGAGGTGCTA
TGACTTAATTATAATTAACTATCTTTATTCCTCTATCTTAACATCTTCTATTTGTTAACA
ATTTCGACGTTTGCTTCTATAGTTCAACCTTTTCAAACTTGGAAAAAGCTCAAGCTGGGC
GACTGCTAATGATAAAATATGATGATCGTTCATGAATAGTACAATAAGAGTAACGGACAG
TGTAAACCATAGGCTTATAATATACTGATAGGATTATTTATACTGATGTCTGAAGATTTC
AATAGTGCGAAGTTCCTAAACAAACAACTGAAAGCTCGGGGACTGCAGAAACTACGATTC
TATTGTCAAGTATGCAACAAACAATGTAGAGATCAGAATGGGTTTAAATCACATGTGAAA
TCGCCTTCACACCTAAAGAATATATCCACTGTGACTCAGGATGATATTTTAGAGTTCACA

Seq prom *SKI7 C. nivariensis* direct
TTGGCGGTTATGGTTGAAATGTTCTTTAAATGGAAGGAGGACTTGATGTGTGACTTAAAC
CCGTTCGCATCTCTGCATTGTTTATTACATACCTGACAATAAAACTTCAGTTTCTGTAGA
CCACGAGCCTTCAGCTGCTTGTTCATGTATTTGGCACTAGTGAAGTTATCCGACATTGTT
CGTTTGTACTATTGCTTAAACTGCTAGCAAGGAATAATAATTTTATATGACTTCTCCCAG
CAAGAGCTTGTTATTCCGTATAGGTCACTATGTTGTCTCTAAGTTTTTGATGGCCAGATT
ATTATTTAAAATTGGTATCCTGCTGATTTTAAAGTAATCTCTTTTTAGGAAAAGACTTTT
TGAAGAAGCGAGTGGTTAAATCTCTTTGAAACCTAAGTGATAAAAGAATTGTTGGCCCAT
CAATAATTTGACTTTTGAGAAAACTATAGTAGTTGTACTACTTAAAGGACAAAAGTGAAT
TATAACATAAGCAAGAATA

Seq prom *SKI7 K. africana KAFR_0D02380* direct
CAATGCATATGCTCACAGCTTTAATATAAATTCAAATATCTGTTATTATGTCTATTCTACACCGGCACTT
CTTGAACGTTTCCAAGCTTTTTTTTTGAGATCACTATAGGTAATGTGGCCGCTACTGAGAAGTTATTCAC
TCCATGGTCGAGTCCTAGGATAGTGAAGTAGGAGCTAAGGTACAAGAAGTGGTAGGATGTGCTATCTATG

ATAATGTTGAGTGTCAGTTGGGCTGCAACCAATTGCCGTGATGGTCATGTTCGATGTTACTAATGCGGTG
TGTGGGTGATATCAGATTTGGAAGAAGTAATCTGCCACTAAGAAAAACCAGTCAATGGATGCCTCTGATT
GTTGAATGATATATAGCTTAGGCATATAATTTCGCCATAGGTTGGTATTATATCCAACGTCTACTGACCT
TCAAGATTCAATGAATATATCAGAGCGTTCTAGTGGGCATTCTGCCCCAATTGAAGAGACTAGTATGTGA
ATAATTGTTAATTAACGTAATTGGTAGTTGAACCAAAAATTTTGAACCATATTACTGACTCACCTTTACT
ATTTCATTTCGTATTAGAACCC

Supplementary file S7: Sequences of the promoters from the *GRX3/4* orthologues. The Aft2-like motifs are highlighted in red/orange. The Aft1-like motifs are highlighted in bright/dark green.

Sequence prom *S. cerevisiae* YDR098c (GRX3) reverse

```
TTAGTCTTTGAAATATATTTTAATG
ACCTCTTAGTTTGGCATCTCATGACCCGGCACACATTCTCTGAAAAAAAAAAAAAGTAAC
TGATGGTTCAACAGAGAAGCCACAGTTAAAAAAGGTCCTACCCGGATTCGAACCGGGGTT
GTCCGGATCAAAACCGAAAGTGATAACCACTACACTATAGGACCGTAACAACTGTGTATT
GTTGTATTACGGGCTCGAGTAATACCGCAGTGTCTTGACAATCCTAATATAAACAGTCTT
AGGGAAGTAACCAGTTGTCAAAACAGTTTATCAGATTAATTCACGGAATGTCACTTATCT
TATATATTATATAAAATATGAATCATACTAAGTGGTGGAAGCGCGGAATCTCGGATCTAA
ACTAATTGTTCAAGCATTTATACGTTTGGGTAGTTCAGCTAGGGAAGGCCGTGTTTTATC
TCATGTTGTTCGTTTTGTTATTGAGATATATGTGGGTAATTAGATAATTGTTGGGATTCC
```

Sequence prom *S. cerevisiae* YER174c (GRX4) reverse

```
ATTTATTGGAGGAAGAGCACAGAAATTTAGTTATATGTAAGTGGAAAAATGA
AATATGCTCAGTACCCAAAGACAACAATGTTACCGGGAAAACGAATCAACGTTCCTGGCA
GTAACAGATTAAAAAATGGTTCAACTGGTACAGTATTAATATGCTATTTAAGCTAAATAT
CAACCAGTCTTCAGATATGGTAACTTTTTCTAGGACATCTGTGAATACGTCGCGAGGCTT
CACCAATTAAAACGCGCGCTCTTACTAAAATTTCACGAATATAATGCTTATTGGCTGAAA
TTTTCAGACCCTTTTGCCAAGTTTGTAAAAAGTATTCCAAACAACTTCAATTTTGGTGTT
GGTGGAAAGTTCCGGCCTCCTCCTTAGTGACTCTTTAATAAACCAATCTGCTACATCTTC
TTTATCTTTGTTCTTTGGATCCTGCTTCCACTGATGGTAAGCGCTCCAGGTTCTGACATA
ATCTGCAAACTCCACTAGGGTGACCTGCTTTCTGATTAGCAATGGCTTCTTTGTATGCTG
```

Sequence prom *Saccharomyces species* GRX4 reverse

```
>Smik:YER174Cu_YER175Cd [1/Smik_5.346/-1/Smik_5.347/-1]
CTTTTTTGGAGGGAAGAACGTAGAAACTCTGCAATATGTAAACGCAAAAATAAATTATACTCACAATCTCAAAAA
AACAATGATACCACCACAAAAAACAATTAACTTTAAATATAATAGGCGTTTAGAAGAATAATTTAATTAGTTATT
TCTAATTTGCTATTTAAGCTATATATTAACCAACTTGAAGATGAAATACCTTCTGTAGGAAATTTGTGAAATAAT
TTACGTCGCGGGGCTCCGCCAATCAAAACGCGCGCTCTTACTAAAAATTCATAAGTCAGATATTTATTGGCCAAG
ATGCTCA
>Skud:YER174Cu_YER175Cd [1/Skud_5.313/-1/Skud_5.314/-1]
CTCACTTGAATCTAAACAGTAAGAAAGAGAAAAATTATGCTCAGAACCCAAAACAACAATGCTGCTACCACTAAA
ACCAATTGACGTCCTTGATAGTGGAAAACTGAAGAAACTATTCAACGGCTACATTTTTAACGTCCTATGTAAGAG
CTAAAATATTTAACAATTTTTCAAATGTAATAGTGTTTTCCAGAAGCTGTGAATAATTAATGACGCGAGGCTCCG
CCAATCGGAAAGCGCGCTCTTACTAAAACTTCACAATTTGGGCGCCCATTGGACAAGAACC
>Suva:YER174Cu_YER175Cd [1/Suva_5.308/-1/Suva_5.309/-1]
CTGGCTTGAAGGGAAAAGGATTGATATAAAACCTGTACGTCAATGAAAAAAAAATATATTTAGGGTCAGGATCCA
AAAACAAAAGTACCTTTAACGGTGAAAGCAATTGGTGTATATGGTCTTTGGAACCACTAAAAAAACCGATAGATG
TGTTTCTAATGTTCTATGTAGATTAAATATTTCACCAATTTCTAGATATAGTACCTTTTCCAAGAATCTGTAAAA
CACAATTAACGTCGCGGGCTCTGCCAATCAGAAAGCGGATCCTTACTAAACTTCCACGAGCGGCCGCTCATTGGC
CGAGACGG
>Scer:YER174Cu_YER175Cd [1/Scer_5.340/-1/Scer_5.341/-1]
ATTTATTGGAGGAAGAGCACAGAAATTTAGTTATATGTAAGTGGAAAAATGAAATATGCTCAGTACCCAAAGACA
ACAATGTTACCGGGAAAACGAATCAACGTTCCTGGCAGTAACAGATTAAAAAATGGTTCAACTGGTACAGTATTA
ATATGCTATTTAAGCTAAATATCAACCAGTCTTCAGATATGGTAACTTTTTCTAGGACATCTGTGAATACGTCGC
GAGGCTTCACCAATTAAAACGCGCGCTCTTACTAAAATTTCACGAATATAATGCTTATTGGCTGAAATTT
>Spar:YER174Cu_YER175Cd [1/Spar_5.371/-1/Spar_5.372/-1]
ATTTCTTGGAGAAAGAGCGCAGAAATTCAGCTATATGTAAATGGAAAAATAAAATAAGCTCAGAACCCAAAAACA
ACAATAATTTTACCGGGAAAACCAATTAACGTCCTTGATAGTAACGGATTAAAAGGTAATTCAACTGGTACAGTC
TTGATATGCTACTTAAGCTAAATATTAACCAATCTCCAGATATGGTAACTTTTTCCAGGTCATCTGTGAACGTCG
CGAGGCTCCACCAATCAAAGCGCGCGCTCTTACTAAAAATTCACGAATCAAATGCTTATTGGCCAAAATAC
```

Sequence prom *GRX3 S. paradoxus* reverse

TTGCTGTAAAGATTTTTGCTGCTATGTACAGTCTTGCTTGGTAGTGTTTGTAAAAAAGAGGTG
AATGAGTAAAAGAAAGTATGTATGAAACTTGAAAAGAGCACACTTAGTCATTGAGATATTCCATTGACCT
CTTAGTTTGGCAAGCTCATGACCCGGCATACTTTGCTGGAAAAAAAAAAATTTCGTACAGAAAAAGAGCCA
CAGTTAAAAAAGGTCCTACCCGGATTCGAACCGGGGTTGTCCGGATCAAAACCGAAAGTGATAACCACTA
CACTATAGGACCGTAACAACTGTGTCTTCGTTTGTGTCACAATGGTCTAAGAATACCAGGAGTCTTGAGG
GCCCAATTATGATTAAGAATAACGTGTTGTCACAACTCATTTTGACAAATGTGCCAGTATTAAGATAAAA
TTTGGGATTTTATTGTTCATGAGGGCTTATTACTAGGTCTATGATAATTTGGTGAATAATTAGATAATCG
TTAGGATTCCATTGTTACTAAAGGCTATAATATTAGGTATACAGAATATACTAGAAGTTCTCCTCAAGGA
TATAGGAATCCACAAAAAGGGAATCGATAGTTCTTCATAGTGTTATTAATTTATCTTCTCTCTTTTTATA

Sequence prom *GRX3 S. kudriavzevii* direct

TTATTTTTACTT
TTTTTTCACTCACCTGTGGCGTTTTTNCCCCGTTGCGCCATCATTTTCGAAATGAATTTTTTTTCAGCTG
TGTCATGCCGGGTCACGAAATTGAAAAACCGCAAGGTCAATAGAATATCTTTATGACTAAGTAAACTTTC
TTCAAGCTTTAAAGATACGTTTGTTTCATCGTTTTCCTTCCCCTAACTACAAACACTCTCAATAATACTA
TACAGAACAGCAAAATTAACTATAGCAA

Sequence prom *GRX3 S. uvarum* direct

AAGATTTCTCAACTTAGCAACAAACATCGGCTGACCTGTTGATTTCAATTGGTAATAGAGATTGATATGT
TTATGTACGTTTATGTATGCCAGAACATGATGTTCATGTTTCTGGGAGAGGTGATTTCCTTATCATAGTA
TTATTCTCTAGCTTCAAGGAAAAATGACCTTTATTCATAAGGTTGTATAATGTTGCTGGTAGTTTCAGCT
TGTTCCGGCAAAAATGTGTCACCCCCAGGGTTTTGATTGGTTTTTGCCAGCCTTTTTCCTCTCTCTTTCA
TAGTTCTTAAGGTCCTATAGTGTAGTGGTTATCACTTTCGGTTTTGATCCGGACAACCCCGGTTCGAATC
CGGGTAGGACCTCTTTTTTAATTTTTTTTTTCTCAACTGTGGCATTCTACACCACGTTTTCTTTTTTTTT
TCATCAATAGTACCGGGTCACGAGAGTGCCGAGCTACGAGGTCAATAGAATATTTCAATGACTAAGCACT
CTTTCCAAGCTCCAATGATGTGTTTCCTGTTCAGCCATCCCTCCTCCTTGAACGACCGTTGAAGATCAAC
TATACACAACAGCGAAGCACTTTTATCAA

Sequence prom *GRX4 C. glabrata CAGL0G08151g* reverse

CACTGCCAGACT
CTCAACTGGTCCCTATTTATACTGCCCGATGAGCTCGGATTACAAAGGATTTAGAAGCGA
TCCCACCGTCGTACAAGATAATGGTAGCTGTGGGGGGG**GGGTGT**GTCTCGGTCTCAGCCA
ATCACCGTCGAGTGCATTACTAATCGCCACCATCGACAGCCAACTGTACAGCCCATCGCC
ACAGCCCATCGCCCCCATCGCCACACTTAGTTGTGTTGTAATTGTATCTGAAGTTGTAGT
ACTTGTGTTCCTGGTTTGCTGGTTTGCTTGGTTTGTCGGTGTATCTGCAGTTGTGTATTT
GCAGGTCTGTGACTT**GCACCC**AGACATCATGTACTTCCCCCCCCCCCCAAATTCCAAAAT
TCCAATACAAATCCTCTTCTCCTGCGGAATCTCCTATTTAGGAAATCTTGGTGTTTCTGC
ATTTGCAAGTGTATCCCTTGGTACGTGTTCATGTGTATCCCGTGTATCAGTACCTTGATT

Sequence prom *GRX4 N. delphensis NADE0s05e04961g* reverse

TTCTTATCTTATCTTTTTTTTCTCTTTTTTTT
TTACAGTTATATATATTTTATTTCGAACTATTCTCTCAAACTATTCCAAGATATTGTTTC
AAAAATAACCCTGCAGCTGAGATATATATATATATATATATATATCTATATCAATATATT
TCTTTGATTACAAATTCACTTGGTTTCATTGCCAGATGCGTTCACATTGTCTTCGATCAT
TATTAATCTCATTGAGAGAGAATGGCAAGAAAAAAGTTTAAACCAAAGCAGAAGAGAATT
GGGAAAAAAAAAAAATTGTGTATCGCGATTGACGAGGCGGTAAATATTATAGCCAATCAGA
TGCGAGAACATTACTAAGTCGGTTAATTTACCCGGACAAATATTTTTTCTTGTGACTGTG
C**ACACCC**AT**ACACCC**ACATTTACCACCCGTACATTTCTAAAAATAAAAAATTTAAAATTT
TTTTCGGAAAAATAATTTTTTTGGACGACGCAAAACAGGGTAATGATAGTTCAATACGGA

Sequence prom *GRX4 C. nivariensis CANI0s20e02904g* reverse

TTTCCAACTTTTTT
ATTTTTTTTTCTTTCTGTTCTCCTTCTCTGAAAGTATAATAAGGTACAAATACCAAACAC
TTCCCTATTTATAATTGTAAGCTCATCACAGTTTGATTACAAATTAAAGGCTAACCACAA
GCTATCAGAACTGCTTCTTCAGAGGCAACGAGAAAGAAAAAAAAATGAAGAACGCCGTAG
AAATTGTGTGGCATCAATCAGGGTAACTAGTCACTCTTTCCCCACCAGCCAATCAGATGC
GAGAGTATTAGTAACGTTCTAATATGGCCTGTAATTGTTTTGACTGCCCCTTGGATGTGT
ACACCCAT**ACACCC**AAAGACTCTCCTTAGACCCCCCCAACCTTCCTGCATTCCAAAACCA
GTTACTGTATTAGGAAACCTTTCTGTTCTTCCAATTTTTTGTCTTTTTTTTGTTTTTTGT
CAGTTTTCCTGTCAATGTCGCAAAACAGGGTAACTATCTTATCTGACGAGAGTGATCACA

Sequence prom *GRX4 C. bracarensis CABR0s37e09009g* reverse

TTTCTTTTTTATTTTTCTAGAGTGTTTTTTTT
TCTTGATTATTACACTTGAACGTCTTTGATAATTTATTTGATACAGGGAGGTTAGAAGAC
CTATTCCTTATTATAGTTTATTTTGTTGAGTGTGTGTTACTATTAATATATAAGGTCTAT
TTTTTAGAACAAGTGTCTATTATATCCTCCTTTAATTTGTTCTTATATATCACCGTTACA
ACTAATACATTACTTATATGTCTATTTTCATTCTGATTACAAATTAGAAGCAGAAGAAAT
TTAAATTAACCTTCCAACTACGCTTTCTACGGTGTTTTCTATACGCAAGTTAAAGAAAGC
ATTGAAATATCTCTCTCTCTCTTCACTTAATACCCTATCTTCAGTAGATTTAACAACATT
TGCGCCTTAGAATCCTACGTACAGATATATAGCTCATCCCAATTAAGTTTTTTCTGTTCA
TCTTTTTGTTCTTCGTTTCTACGTCAAAACCACAGTTTGTGGCGAATTGGTTATTCTTTA

Sequence prom *GRX4 N. castellii NCAS_0B03970* direct

ACAATAGCACATACCTTTTCCTTGCAGTATTAGTCTCTTATGGGGGTTCAAGACAGGAGA
AGTGCGAGAAAGAGGTTCAATACATCTCCAAGTAGTAATATAACGCCAATCTAATCGTCT
GAGAATTCTTTGTCATTGGCCCAAGAGCCTCCGGGTAACGGCCATCGGTCACCCGGATGG
CCGTGCTGTTAGTATATACATCTATAAAGAGCTATTACCACCATTATTCAAGGGCACTAA
TGACCATATGTTGAACCAGTATACATCGATACCATATATCTATAACATTGTAGGTTTATT
TCACCCTATAGGGTTGCACATTACGTAAGCCACAGAGAGTCATATTTCGATGCCACAGTC
GCCGTTTTGAAATTTTCAGGATTACAAATACAACAACTGGATCTCATCTCATATATAAGC
CAAGTCAAATCAAGGTTTCCATCTGCTTATCAGTTCTCGCCGACCAACTCAGCAAACCAC
AGCA

Sequence prom *N. bacillisporus NABA0s32e00154g* direct

GAACCCTAAAACCCTTTCCGCCCTCATCACCTTTGACGTTCCGCTATTGCTCTGTTGGCA
CCTGTGGCCCACACTACGGTATGTCCACAGTTGACTCCTTTGTTTGTCAAGTTTTACTGG
TTCTATAGTGTAGTGGTTATCACTTTCGGTTTTGATCCGAACAACCCCGGTTCGAATCCG
GGTAGGACCTCAATTTTTTCCAGTCCGCCGACGCAGAACTCTGTAGCCCAGTCTTAAGTT
ATCTAAATGCTTTTGTTCTCCGGAGCATATGGCCGGGGACCGTTGAGAGGTTGTCACAAG
TGGTCCATCACGTAGAATGGTCAGCTTGCATTGTTTATTAGGCTGGTTCTTATCGATGAT
TGGCCAGATCACATGCCTCCTCTTAGTAAGCTGTTATTTTTTTTGGAACGTTTTATTCCA
GAATTCAAATATTATATAAAAAGAGGTAATGAGGGTACAGTACTTCTTGAACGGGACAGG
GCCCAAACAACAAGCAAATAACGCTTAGAGGGACAAGATATA

Sequence prom *GRX3/4 Z. rouxii* reverse

CTCGATATAGCTTGATAAAGTTTTAGATCTTTCTTCTATCCCTTTGTTTCTC
TTCTTATCACTTTAAATTAAGTGAATGTGATAGAATCAACGTTAGTACTAGCCTAAAATG

TTATATAGAAGTATCCGTTAGTAATTCTTCTAATATTCGCTTATTATAGTTCTACCCATC
TCGACGGAATTTTTCGACAAACACGAGTCTCTGAACGTGACCACTGGCCAATGATAGAGA
AGACAGTTACTAATTCACGATATCCACGAACTACATGATAAGGTTAATGGAGTGTGCTTA
TCTGGTAATTCCAATCGTTGTTAGTTCCCATAAGCTTATCAATCAGCGCATTCTAGTACC
TTCTTCTGGCAAACATCCAAAACGTTCTCCAGGTGATTTGCAACTCCGAATTCTCATTCA
ATTGAGGGTACAATTCTATGGCACGATCAACAAACGCATCTGTGATATCCTTTTCAGCAC
TAGGGTTATCTTTCTTCCAGGAATGGTAGCCACTCCAACTCTTTATGTATTCTTTCAATT

Sequence prom *GRX3/4 Z. bailii* direct

TTGGTTGAAGTATTTTTGACGAGCTCAAGGAAGGTTTGCACTTAAGAAGGTCGAGCATAA
ACGTCGCTACATCATTTCGAACTTTTCCACTTTGTTATAAAATCTGAAGGGCTTGGTCAA
TAATTAAAGTTTACGTTTGTTTTCTTAGGACTGTCAACCATACGATCTGCTGTCCCACGA
CGTGATCTACTAAGTGTATCTTCTCCCCGAATCCAAGCTCTAACCTTATTATGGCAGTGT
TAGATCCAATTGAACCCTTATGCATTCCTAGTCTTTAATGCTCTAAAGTTGATAGCATCC
ACATCGATGTTATTATGCTTTCCCTTCCTCTTACTAAGTAAATCGGGACTGATTGGTCTA
ACATCACATGATGAAAAACAATATGTGGTAAATGGAAAAATCGTCGAGAAGGGCTAAATC
ATGATTATGCGCAATACTAACATTCATTAGACTCGACATATATTGCTTGATTTTTTACAT
TGATTCTTTCCCCAAAACAATTTCAACTTACTGAATCAAGATTGCC

Sequence prom *GRX3/4 L. cidri* direct

TGAAAACGTTGAAACTTAAGCAGCGATGATACACTGCACGCGCTATAATAACGTTTCAGT
GGCAATTCAATTCGCTAGACTTCCAAGCGTCTTCGAGCACGTGATCCATCATGTCGAGAT
ATATATATATATATATATATATATATACAAAAAAACGTCTTGCCATATTCAGAGCGCTGTCT
AATAAATCTGAAGCTCTCAAACGTATATTACGTAGCTGTTATACCCATCGATTGCCTGAG
TAGTTCTAAGTTAATATCCGGAGGGTTTTAAAAACAAAATCATCAATGGTTATGCGAATT
AGTAATGGAGAAAAATTTCATTGGATATATGTCAGGTGATAGCCGAGAAAATCGACGAGG
TGCGTTGAACTGGTT**GGGTGC**GGTATACTAGAACCGTTTTCGATATAGTCAACGTGTGTC
TACTTTTTTAACAAGAGCAAGCAACTATCTCAACGAAGTGGCAAGCTACTTGTTCCACAA
TTCCATCCCGTTTGATCTCTTTTCATTGTTAAGGTAGA

Sequence prom *GRX3/4 L. fermentati* direct

GGTCAGCCAACACCAAGTCACCAAACTACAGAGTTAAATCAAGCCAGGCAGAAGTTTCAC
GTGTGAAATTCGATTGCAACATGAACAAGAGGTCTTTCAGAGGGTCATTCGCTGTTACTC
AAAACACGTGATCGCATCCCATTGTATTCTCTAGCGAGCTTCTTGGCTTGGCATAACCCT
AAGTGTTGATTTGCCGTGCCCAATCTGCTTCTAGGCTCACCATCAACTCCTATATCGGCC
GTTGGTAAAAGCAACGATCGCAATTGGCATTCTACAGGCTTTCTTACTGTTCCACTATAA
GCATCTAATGCGAATTAGTAATGAGACAAAATTCTATTGGGCTAAAGTCAGGTGATGCGC
TTGAAAAAGCTGTGAGGTGCATTCAGTAAGGTC**GGGTGC**GAAACATAAGCACGGTTTTTG
ACCAAGCAAAACGTGTAACATCTGAGCTTTATTAAGGAGCGACCTTTGTGCAATTGCTAA
TCTCTTTAATCTCATCTACTAAGTTGCTATTCCTTGGTAGTTATCAGAAA

Sequence prom *GRX3/4 L. thermotolerans* direct

TCTGTAAAGGGTTTCTGGGCAGGTTGCGGCTAAATACAGGGACCAGTTCAAGTTCGACTT
TTACAGTTGCCTGGAATTCAGTCTACAAGTTCGCCAAAAGGCGAGTTTGACCTCAAATTA
TGCAGGATGGCACGCTGAACCAGATGGTTTGTTTCGAAGGACAGAAAACGATGCCAACCT
TTTTCATTTCCGAAGTGCCACCGGTTAGCTGACTGGACCAGTGAATTCCTTGCGAAGTTT
GTTCTTAGTAATGAAGTCTACGCTGATTGGATACCAATCACATGATCGAATACTCTTGAC
GGTGTTCGTCACGAAGGGACCAAATATAGCGAAATGCGGGTATAAGCAGCATTGGAATTC
AAAGCTAACAGAGCCTTTCAGAATTAATCTATTGTAAGTAGAGGCTCGATATTGCTCTTG
AAATAGCTTTCAAACGTTAGTTCAAGCTTTCTGTGCCAAAGACAAAGAACTATACAACAA
TTTGCTTGAA

Sequence prom *GRX3/4 L. mirantina* reverse

TGTTTT
GTTGGTTACAAGAGCTCCTGAGGAATTTAATCAACAGTGTAATTCAAACTCTCGTCAAGG
GGATTGGTTTGCGTTCAAAAGGTTATCGATCCAAACGTGGTGATGAGTTGTCAGCATACC
ATTATAGTTACTTGCC**ACACCC**ACTATAAGAGTTCTAGAAATCCAAAGTTTGTCACGTGC
TTTCAAGCCAATGATATGTGGTTTCATTACTAATAACAAGCAAAACGACATTATAGTACT
CTTAAAGGCCTCGTACTCGAAGCAGGGGTTAGTCCCCATCATAGCAGCGTGGTAAGAATC
CGAAATGCCCAGAATGCTGCCGTTCGTCTCTTGCTTGAAATGAGTTTCACCAAAAAGGGT
AGAATGCACCAATTTCACAACTGCCGCACAAGGAGCCGCTGATAGAAAAGATCAAATTCG
ATGGGGAGGTTTTTCATTTGTTTTTATTTTGAGCAGAGCAGAGAGTTCAGCCAACGATGC

Sequence prom *GRX3/4 L. dasiensis* direct

CCATCAGAGGGAATAAAATGGGAGAGGGACTACTGTGTCTTCCACACGTTGGATAAGCGC
TTTCAATTTATTCATTTGTCAGTTCGATTATTTATGTTAACAGGGCACCTTATTGACTTT
CCGGCATTGGAAACCTTACTCAACTCCCAGAGACTTGCGAATAATCATTTCAAAGATGAT
ACAGAGCCTTTATAAGAAACAGTGGGCCCCAAATATTCTGTTGATGCCGCTTACTAATAG
AACCTTGTGTGATTGGTCAGTGATCACATGACATGGAGCTGTGTCTTTACAGTCATTTTC
TTGGTAGTTCAAATGGCTAGCGATGCAACTTGTTAAAATAAGAGTTGGGTGGGAGCACCG
TAACAGTTAGATTCTTCTTGTTACATATTCAGGTTCACATCGGATACTAACTCCCAATAT
CTGGAAGAGGAAACTACGCTTAGCTGTGGAATTCGAGATATATTTCAGCGGATTTTAAAA
CTTCAGAATAACCTTTTCAAACTCAAACATATACCGCC

Sequence prom *GRX3/4 L. waltii* reverse

TATGTTAATG
AAATGGGGAAACTTTTATCTCAGTGTCTGTGTTAATATGGTGGAAACAGGATCTGTCCTG
GAAGTAAAGGGAGACGATTTTGGTTTTCAATTGCTC**ACACCC**GTCACTTCAGAGACTGTT
TTAATCATATTGGTGGCAGGCCATGAAATGTGGTCAAATTCATGATTTTATGTGTCAGAT
GATTAACAGCCAATCAAATTCTAGAACATTAGTAATACACACCAAAAAGTTCCAATTCAT
GTGTGATTTTAAAAGAAGGGTTACGATGACTTCATGGCTCACTCTACTTTAATAAATCAA
GTTCCATTGTCATCAGCTTCAGTCTCATTGCCAGATTCTTTGTAAACGAATAAAAAATAT
TATCGCCTTCTTGCAAGCTTGTTGACAGTGCTCCATGCGACCTTTAAAGGAGTATTGGGA
TCCTGACGAGTATGGCCACTAACCCGGCGCATGAATTTCTTGCATATGTCCTGTTTTTTT

Sequence prom *GRX3/4 K. lactis* reverse

ATTTAGTTTGATGGA
GCAAAAAATAAGATGTGCTCTTGAAATGGTTTGTCAGAAACGGTTCGTTGAACTATAGTC
GAAAAGAAAAAAAACTACCCTCGTAGAATAGCTCTCCCGCCAGTTTAGATTAATAGATAC
TAGATTATCACTGAAGTTGTTCTAGTCGCACAGAGTCTTTTATAATCGTGCTCTGTAACC
CTTTAAACGATGTGTTATGTATAAAGCCCAAATTTTGTTCAGAATGCTTGAAATATTTCA
CTAATTTCAATATTTCACAGTTTCACCCAGAGTACGCAAAAGACAACAGACGAAAGAGAT
GAGATGAGGAATGTGAAAACATGCGTCGTCAGCACCGGTTGAAGTTAAATTACATGAATT
TCAACTAATTCCGTGCATTTACCATTAGTGTAGTGTCTTGCTAATGTTTGAACAAAAATA
CAGATATCTTGCTTATATATATACATATATATATATATTCAATACGTATTGATAAGAGAG

Sequence prom *C. castellii CACA0s33e02508g* direct

ATTTTTTTATTCGATGTGCTTTATTTCCTCTTATCAAGACCCCCACCTAGAATTGCAGGG
ACAAACGCGATAGACTTGTCATAGTAGACTAGAGCCTGCAAATGCCCACTTACCGTCCAC
GAGAAACAAGGCTTCATGAAGCTCAGTAACTACTGAAGATTAGTAATTTCCTTTGTTTTG

ATTGGGTGAAATTTCGAATATTTTCGAAAAGTTCATCATTACAAAGAAAACCTTTTATAA
AGTAAGGTTATACGGACACGATGAATATCGATGAAATGACATGTATCAACGATACTATCA
AGAGTGAAATATCTGTTACCCTCAATATTGCAAGTGGTTTTTGAGTTTTATAGTTATTAG
TTCTCGTTTGTACTTGAATAAGCTCTATAGCAAAAGTGATAGTTACCTGCAACATTTGGC
AACTAGTATAGCATCAGTACGGCATTACTTGAGAACACAGTTCAACCGTAACTGAACAAA
GAGATAGAGTTAGAATACGCAACGCTGTAAGAGGCACCATAAAAAAATAAAAC

Sequence prom *T. blattae TBLA_0F03860* reverse

TGTATTTTAAATATTAAGCGCAATAATTTATTTTTTTAAT
TTTTTTTTTTATTCTTTGAGAGAAAAGAAATAATGCTTAACCTCGATAAAGTTATTTAGA
ACAATGTCTTAATATATACTATAATTAATAATAGTTAAATAACTTAACTCAACGACTCTA
CAAATCTAGTATTAAAAGTTCGTACATTATTACTAATCGAATTGATGAGACACCTTTATT
TCAATCGTGGGTCAACTGGTGTGAATAAAATTCATATGCAAATTTGCGCAAGTGGTATTT
CATACGACATCCAATCAAAAACGAGTTATTTAGTAAGCCGGAGATAAGTAGGGTTTCGGG
TTTAGAAAGTAGGGTTCACAAGGGTTAGATTTTTAAAATCTCATAAACATATATATAGAG
GATAAATAAAGATATTCCGAAGACTATGTGTGCCTGTTTCTTTGTTGTATATGAGCTGAT
CAGATCCTTCTTGCAATCTTAAAGGCAATGGAAAGTTTGAAATTTATCATGGTTACGCCA

Sequence prom *GRX3/4 L. meyersii* direct

AAAGTCATAGAAACAAGAGCTAAAAACACACAGTTTTGACGACTCCAGTAGCGACAATTA
CGACGACTGAGCGAGCAAAAATTGCGAGTCGATAAGCTTTAGATATACGTGTAAAAGCTG
GGTTCTAGTAGTCTTTTACTAATGCGAGCGGCTCTTATTGGACACAGATCACGCGACCCA
CCGCCCACTATAGTTTTTCAGATTTTGAGATTTCGCCCAGACATTTGATTATAGCCTCTG
GCTTGATCAGGACACGGCTGTTAATCTTTTGATCTCATTACAGACCACAAAGTACCTTCA
AAAACGAAGCCAAATAGGTGGTTTTTGGCTAGTTTTCAAGATCAAATCTCACAGTGTTGA
ACTCGCTAAAATTTGACCTCGACACCGCTTTTCAGGCTAAATTTTGGACCATTTTTCATT
AAGATCTTTAACGCGCAGCACCGTTTTAGTATATATTCAGCAGTATAGAACGCCATCAAC
GAACAAC

Sequence prom *GRX3/4 L. nothofagi* reverse

TGTAAGCGATTTGTATGTT
GTTGGATGAATCTCTCGAAGAATGCTCTATGACTTTTCGGTCTATGAAGAAGTGCCTCAA
CAGTAAACTCGAATCCCTTCCTAATTATTATCAGATACAAAAATCTTCCGTGTAAGAATA
GAAGTCTTGTAAGTATATTGGTGTATGCAGATTTAGAAGCCTTACGGTGGAGCAGTTAAT
AATCTGCTAGAAACCTAAATGTCATATTATATTCAACGAGGCCAAGGCCTATTTCCATAA
TCTTGCAATTCTAGAAAAACTATTATTCTCACCTGACATTGAGCCAATGAAAGCCACGTT
CATTAGTAAAAAGTACTAACCTGGCGCTACAATGTGCTAATGGATAGGCTCAGAAGATGT
CGACGTTTGTGAAGAACATGGGACTATCATAGTTGTCTTCTTGGCAGTTTCTTGGCTTTG
CAGCGAAAACCTAATTTTCCTAGGTTCTTTTACGGGCAAATTTGTAGACGGTATTCCAAG

Sequence prom *K. naganishii KNAG_0H03030* reverse

ATTCTGTGGTGTGTTCTTGGTCCAACCTAC
AGACAAGTTAGAACTGTATCACACCTATTACTAATCTACTCACCACATCTTAAATACTAT
TACAGTCTCAGCGAACTGCGCAGGTTATATTTTTTTTTTCACTTATCCTTCCACCGTCGA
TCAAAAGGGAACAGAAGAAGAGCAAAGAGGCTCGAAGTGTGGTTCAACCGCGATTCTGGC
ATTGGCTCCTGTCCAATGAAAGGAGAGGATATTAGTAATAATGGGAGAAAAAAATGGAGGA
AAAAATAAAGAAAAAGGTCCTACCCGGATTCGAACCGGGGTTGTCCGGATCAAAACCGAA
AGTGATAACCACTACACTATAGGACCAGACAATTTTGAATTTTTGTTCACGACAATTCTT
TATACATCTTGGTGTGATCGGCGTTACTATAGTTCGATTAAGTCCAAAAGCCCTGGGTAC
TGGTAACTGGAACAAGAGGAGTCCATCTCTTGTTCTGAATAGCAGGAACAAGGTTATTAT

Sequence prom *GRX3/4 L. kluyveri* direct

CGTGATCACTTTTTTCACGAAATATTCTCCTCGACCAAGCTCTCCTGGCAAACAGAAATA
CGTGTTGTATGGAATACCGTTTACAAGTTTGCCAGGAAGCGTTGATGCCCACCAAAAAAA
AAAGCGTCTAGCTACACCTATGCAAAAGAGCTACTATATCCAACCGCTAGCCATATACCA
ACGAGAGCCAATGGGTAATTTTGTATGAGTTATCGAGTAAATACTTGTATTCATAGCCAT
CAGTGGACTCAAAATATAGTTTACACCAAAGCTGTCCGTTTTGCATTACTAATAACTAAT
ATTACGATTGGCTGAAAGTCACGTGAGAAGTTGAAGAGCAAGCAAAGTTGCGCCCTTTGC
ATCT**GGGTGT**CTTCTTTCTCTGGAAGTTGCCAAGTCAGCCGTAAGTGTATAAATATCTTT
CTTCGTGAACGACCAGCTACCCAATTTCTAGTCTTTCAACGAATAAGACAGTTTCCACTT
TTTCCGTATACATATCTTAACCACAAGGGACCTCAAGTGTATCACA

Sequence prom *GRX3/4 L. fantastica* direct

AAAGTCTTAACCAGGTGAAGTCAGTCAAAAACTCCATTCGGCTTAGGCCAATCAGAGCGA
GTTCCTAGCTTTACGCTGGCTTAATGCTGGCTTAACGCTATGCTTACTATGGCCTTTTGC
CGCTTTTACTAATGTCGACTCACCCTATTGGTCTCAGATCACGTGCTCTCATCGATTTCC
AAAAATGCTCTTTCAACTGACTATCACGCCTAGTCAAAAGACACTCATTGATTTTAGAGT
TCGAGCTAGGCCTCATCAGGAGATGAACTAGAAGCTTAGTTGTCAAGTTGAAAAACCAGA
GTTTAAATACCAGGCATCAAGAAGATTCAAACTGGAAAAATTCGTTTCAACTTACAAAAA
ACCAGAACTCCGTGCTAGTTTCTGTTGTTGCTTTGAAAAAAACTGAGTTACCTTCCGAAG
CTAAAGATTTGACCCACCTACTAGAAATACTTGGCAAGAACACTTACACATCACATACAG
TCCTTTAACAACATTTCCAA

Sequence prom *N. castellii NCAS_0B04890* reverse

TGCAGTATTGGGCTAGTTTCTTGGGTAAAGCGTTAC
GTGAAAAATTTGATAACTCCGAAGCTGATCTGATCTGGCGAGTGCAACAAGCGAGAGTGA
AGAAAGTGTTACAGTTAAATAGTCGGCCCAGTACTTAAAC**GGGTGT**TGATTAGTGAGGTG
TTGCTATTTTAGTACCCCAACATGCACAGGGATAAGAGGTGCATTACGGGCATGCACTAG
CCTGTCCATTCTGTGTTTC**GCACCC**CATACTCTCTTCTTCTACTTTTCACCACCAGGATC
AACACTGCTATTGTCTCACAATTTGTAAGCGCGCTTGGCCAATCACGCGCGAGCTTCTTA
CTAATG**ACACCC**GCTTTGCAGAGTGTCAGCAATTAAACAATCATAGCATTTATTACAACT
ATACCCGTCTTCCTAGTTTATAGAATGTATTCCATGCCACACGGACCCTGGACTGAGGGG
TCAACTCA**GGGTGT**TTTGCCAGTATCAACTTAATCAGCTCGTCACAAACATCCGGCTTAT

Sequence prom *GRX3 C. glabrata CAGL0L11990g* reverse

ACTCTCTCTCTCTTGTTTTGTAACTACA
ATTTATACACGTAGTCTTCTGTAAAGTTATAGTCTTACACACAAGAAATGAGACATTAGT
AGTACTAATGCCACCTAAATATATACAAACAAGTTAAACTCAA**GGGTGT**GATACAATGCA
CATACTCCCC**ACACCC**CTCCCAGTTTATCCATCTATAATATACCCCTCCTCCTCACTACA
ACAATCCACCAATATGACTATTAATAATCAAATTAGTAGTCATCCACTTTTATTTGCACA
TTACTGTGCTGTATCTTCGAGCATTTTTCAGTTGGATTTTTTTCACTTAACAG**ACACCC**A
TAAAGAGGCAAATGGAGTTAGGAGATGGTCATCGAGGTGCGCAGATGAGAGT**GGGTGC**GT
TAAGATAAAGACACAAT**GGGTGT**TGTGTAAGTTCTTGATACAATGGTAAAGCTACAACTC
TGAGTTTCTACACTTCTGAGTTTCTACATTTCGTTGGATTTTCCTCTATTTTGACACCTT

Sequence prom *GRX3 C. nivariensis* reverse

TATTTGTTATTTCGTTTATTCTGTTTTAGCGTGTTGGAGGAAG
AAAGTTCTACAAGATTATTGTTGTATTTTGTTTATATAGTTTTTGCATATTTACTTTTCT
ACCTGAGA**GGGTGT**GTAACGTTTTGTTTATCTGTTTGTGG**GGGTGT**AACAATAGGCTGAT
TAATCAATAATAAGAAGTAGTCATCTTAAGCTTTTTTTTTTCATCTTTACACCTCTTGCA

GACTCCCCCTTCCATTGATTTTAT**ACACCC**ATTTTTTTCTTTGTGCAGGTCCTACTTAGC
ACCAAGGAGAAAAGC**GGGTGT**CTTCTTTTAATTAGAACAAAGGTGCCATTGGAAGGGGGG
AACAAACGGCGATGCTCAATGAGCTTTCTTCTATAAACTTCCCAACTTGTTTATTGGACA
ATGCTGCATTGTATTAGTTTCATCGTTAGTACAACTTTAGCTGGTTCGACTATATTCTTG
TGTGCTTTAATTGCGGGGGGGAGCGACTGCTGTTCTGTATGGCTATCTCCTTCATGCAGT

Sequence prom *GRX3 C. bracarensis* reverse

GATATCCAACCACTATTTGATAATGATTGAGCAGCTGAAGTGAGATGGCCACACTATAC
AAACTAAAAACAAGTAATACGGACTATGTATCTGCTCTATATATATATTAATCCCACATA
AGGGG**GGGTGT**GGTGGAAGGTAATACAGCTTGAATCAGAGAGGTGACTAGGCTATTAATA
AGCCATAGTCATCGCCGAAACGCCTCCCATTGTTTCTTGATAAAC**ACACCC**GTATATGTC
TCTTTTCTTTATAACGAAAAGT**GGGTGT**GTGGTGCAGTGGATATCGCCTTTGTTAAGAAA
AGGT**GGGTGT**CTTTATGCAACACAAAGATACTTTTAACAAGGTGCAAAAATCAAAGCCAG
TGGCGATGTGAATACAGTTGAACAT**GGGTGC**AGAACTAAGCTAATAGATCACCAAGTAAA
ATATGCACGGAGTTTATCTCTAATTGTTTCCTTTCACTGATGGGCTGAACTTCATCCATT
TCTATTAAGAGGACATGCTATATTCTTATTAAACGCGGGATGATATATAATACGTACTTC

Sequence prom GRX3 N. delphensis direct

TATCTCCAAATTCGTGTTATTAAATCCGTTAAAATGGTGAGGAATGGCAATTCGATGCTA
CAAAAAGAGAAGACAATACACTGAATGGATGAACTGGTAAATTTCTTTAATCTCCAACAT
TCACATGTTATAAACAGTCGCTAATGCACCTTCTTGGTCTAATATTCACTCTATTTAGCT
ATGCTCGCTACTAAG**GCACCC**GCTTTTTCCCTTAAGAGCCACGCCAACGCGCACACGCAC
ACGCACACACAC**ACACCC**CACGAAGAAAAT**GGGTGT**GAGTAGCTAGACTATGAGAAAGGA
GAGGTGTGTGTATGTAGCAATGATGACTACTTCTTACTGTAAACTTGATTAATAAGGCAG
GCAATAACTC**ACACCC**TATTTAAGATCATCTATATAAACAAGAGAACATGGGATACTTCT
GTTTTATAATGGTGCCTTTCTTGGCAATTTAAGAGAACCGTAGATTCCTGTAACGAAAAG
TATTTAGACG

Sequence prom *K. naganishii KNAG_0G01920* reverse

CGAGTTTATGTGGGTTGGATGGTAAACGGAGTCACTTACAAGCAAGGTCTACATGCATG
TGTACGTGTGCGTGTGTATATATATACACACGCTGCATGCACGGGCCTCCCGGACACATC
CAATATG**GGGTGC**TCTTATCATTATTATTAATCAAACGGTTTGAGAATATTAAATAGTGG
GTGAACAAAAAACCT**GGGTGT**AGTGTCCCTGGCGTGTCTTGGTGCCCCCCCCCCCCTGAT
C**ACACCC**TTCTTGCAAACTTGTAGAACGTGTTCCATACTAGGTGTACCTCACTATCTAGT
TCCATTCCT**GGGTGT**CTCTGCATGATCGCGTGTAGAAACTCATCGCATACGTCTGTTTTG
TCTTTGTTTGCTACGTCTGCCTTCCATGAGTGGTACGCGCTGAATGTACGTAGGTACTCT
CCGTACTCCCGCAGAATGCACCGTTTCGTGATGGACAGG**GCACCC**T**GGGTGT**CTGTTGCA
TGGGAGTAAACCTCTTGGACATCCCTGAACAAGTCCTTTC

Sequence prom *GRX3/4 E. gossypii* direct

GAGCAAATGAACAGCTACCTGTCGACGTGGAGCGCTTTCCACTCCTGGAGGAAGCAGGAC
CCCGCAGGTAGTATCGCCGCGAAGTCTGCGTTTTTCCAATTCATATTCTCAAAGACCTCT
ATGTCCTGGTCCTCTGAAGTATGCCTGACCTGGAATAGTGTCGGGATCCTGGCCAGAAGG
CGCTAGGCAGACATTTGAGCCCCGCGCCGCCCCAGTAAAAAGAACACCAAAGAGAACCAA
CCCCAGCCACTTCGTAGGCCTTAGATCCACTTATCTGACTGATAGCAGCCAGCAATTAGC
GACTATGTATGGTTTATGTATGACTAATGTAGTGTCACGCCGTTGGCTGTTAATCACGTG
ATTTATGTTGAAGTCTTGGTGTCTGTTGAAATGGAAAATTATGCATGGTTAAGGCAGAAC
TTTACGTCTAGAGCGCCAAGAACTCTAGACTATTCCTAATTGTTTTACTGACTAGAGCAG
ACTGTACATTACTGGAT

Supplementary file S8: Sequences of the promoters from the *MAK16a/b* orthologues. The Aft2-like motifs are highlighted in red/orange. The Aft1-like motifs are highlighted in bright/dark green.

Sequence prom *MAK16 S. cerevisiae* reverse

```
ATCCTCGTTGTATTC
TAACCGTTGTAGTTATGTACTGAAGAGAACACTGTCAAAAGAAAGAACTAAGCAATGCAA
TATCTGCCTCTATACCAATCACTTTTTCATTTTTTTTCAAAAGCTCATCGGAAAATTTTT
CAAAAAAAAAAAAAAAAAAAAAAAAGGTTTATTACCCTACTGCATTTTGATAATCTGAAC
ATAATGAGCTAATGAAAGCAATTCTCATTTAAAAACAAGTATTCTCTCTTATTGAAGTAT
GCATTATCTATCATTATAAATTCTTTTATTCTGTTCGAGTCCATGTTTTTAAAAAAAAAA
AAACATGTATGTATGCTCCATCTATATATGCTCCATCTGTATATTTTATATGCAAAGTTT
TTTACAAGAGGAATTTGGGAACTGGAGGAAAGTGGCACAATACCTCATGTGGATAGTTCA
TTAATCTCTTCTTGTGTTAATGTGCTAATATAAACACACTTACTCAGCAATTCGTGGTTA
ACTTTGAAAGTGTAAAACTTGGACCATTGAACCCTTTGAATGAAATTCTTGACTATTTGA
ACGTTCGTATCGAATTTATTGTAATTCACTACTTTATCTTCTAAGATCCAGTCTTTCTTC
```

Sequence prom *MAK16 Saccharomyces species* reverse

```
>Smik:YAL025Cu_YAL024Cd [1/Smik_1.41/-1/Smik_1.42/-1]
ATTTCCTCGTTATATTTCTACAGTTGTACGTTATAACAAGGCAAACTCCACCAAACGAAATTTTAAATAGATGCA
ATATCTGTCTATATACCAATAACTTTTTTCAGTTTCTTTTAAAAAGCTCATCGGAAAATTTTTCAAAAAAAAAAA
ACTGTCATTACCCTACTTGTTTATGTATTCTGATCGTAATAAGCAAATGGAAGAAAATTCCGGTTCTCATTTTAA
AAACTAACATTCTATTTTAATAGAGCATGCATTGTCTATCGTTATAAATTATTATATTAGGTATGAGTCTATGAA
AAAATATGCATATGCTTGACTTCTATGTTTTATAATCAAAGTTTTTACAGAAAGAGAATCTTGGTACCAGAAGAA
GGGTAGCATGCTCC
>Suva:YAL025Cu_YAL024Cd [1/Suva_1.41/-1/Suva_1.42/-1]
ATTTTCTCGTTATATTTCCACTTGCAGCTACCTGTCTTCAACCCAACCCAAAACTAAAGAATATAAAAGATGCAT
CATCTGCCTATATACCAATAGCTTTCTCAAATTTTTCAAAAAGCTCATCGGAAAAATTTTCAAAATAAAAAAAAA
AATAAAAAAAAAAAAGTAACTCATTACCCGCCTAATACTTTACTTCTAACTATAATAAGCTTCTAATCATGGGAG
AACATGCTTTTGAAACCAGTATTCTTCCTGCCCTGCCATGCATGTTTTATTGTCATAGTTTGTTTTGGTTTATCT
ACGATGAAAGAAATTATATGCTTTTTCTATATATTTTATATGCAAAGGTTTTACAAGAAAAGAGTTCTTGGGTAT
TAAATGTTCGTTTTGCGGCGTTCGTC
>Spar:YAL025Cu_YAL024Cd [1/Spar_1.44/-1/Spar_1.45/-1]
ATTTCCTCGTTGTATTCCCACCGTTGTAATTTTCTATCAAAGAGAACACCACCAAAAGAAAGGATGAAATAGTGC
AATATCTGCCTATATACCAATTGCTTTCCCAGTTTTTTTTCAGAAAGCTCATCGGAAAATTTTTCAAAAAAAAAA
AAAAAATTAATTACCCTACTGCTTCCTGATACTCTGTTCATAATAAGTCAGTATAAGTAACTCTCATTTTAAAGA
CTAATATTCTCTTTTATTGGAGTATGCATTATCTATCATTATATATCCTCTTATTCAATTCGAGTCCATGATCAA
AAATATATATGCTCCATCTATATATTTCATATGCAAAGTTTGGAACTGAAAAAGGATGGCTTATTGCC
>Scer:YAL025Cu_YAL024Cd [1/Scer_1.63/-1/Scer_1.64/-1]
ATCCTCGTTGTATTCTAACCGTTGTAGTTATGTACTGAAGAGAACACTGTCAAAAGAAAGAACTAAGCAATGCAA
TATCTGCCTCTATACCAATCACTTTTTCATTTTTTTTCAAAAGCTCATCGGAAAATTTTTCAAAAAAAAAAAAAA
AAAAAAAAAAGGTTTATTACCCTACTGCATTTTGATAATCTGAACATAATGAGCTAATGAAAGCAATTCTCATTT
AAAAACAAGTATTCTCTCTTATTGAAGTATGCATTATCTATCATTATAAATTCTTTTATTCTGTTCGAGTCCATG
TTTTTAAAAAAAAAAAAAACATGTATGTATGCTCCATCTATATATGCTCCATCTGTATATTTTATATGCAAAGTTT
TTTACAAGAGGAATTTGGGAACTGGAGGAAAGTGGCACAATACC
>Skud:YAL025Cu_YAL024Cd [1/Skud_1.36/-1/Skud_1.37/-1]
ATTTTCTCGATATATTCCCACTGTTGTAGATCTCTAACAAGAAACTATCACCAAAAGAAAGAGCAAAATAGATGC
AAATACTGCCTATATACAAGTAACTTTTTCAAATTTTTCAAAAAAGCTCATCGGAAAATTTTTCAAAAAAGAAAA
ATCATTACCCTACTGGTGTTTCATTTTCACCGTAATAAGCCAACAGCCAGAATCATTGCTTTTATTTTGAAGTTA
ATTTCCCTTTTGACTGGATCATGTATGATCTACAATTATAAATTATTTCATACGTTTTTCATCCTATGATCAAAA
ATGTATATGCCTCATTCTATGTATTTTATATGCAAAGTTTTCGACGAAAATAAAGATTTCTTGGAAAATAAAGGT
TTGTGGCTTGCTTCC
```

Sequence prom *MAK16a C. glabrata* direct

```
AAATTGAACATCTAACAGCAGAATTCGCAAGGTGAGTTTAACTTTATACTGCTTCAAAAA
ACTACAAATTCTCCAACCGCATGTGCTATAATTTCATATATAGTTAATTTTTTGATGTTT
GTTTTCATTTTTATTTTTTCTAAACAACTAGAATAAAATTTTGGGAGTAACAGAAACCGT
ACATTATCTGCATAGTTTATACACGGGTATAGACAACTACTAAATACTATATAGTAAATT
```

AATTTATTGCATCTCTCCAACTACAACTGCTTTTATATTCTCTCCAAGGCGCAACCTTTA
TTCTACATATGAACTGACCTCCGGGTAACCAATATTCGCGATGAGCTCTGGAAAAAAATT
TAAAGAAAATTTTTTGCGATGAGCATCTCGATGAGTTTAGTACTGTAACGGCTAACAATC
AGTGGAACTTTACCATCTGTAAGGCGATCTCTCCCTGATTAGCCAAGGATTATTCAT**GGG**
TGTTTTAGTAATCTCGGTGCATTTACTTATCTGATTGTTTAGTTATAGATAGTCTAGAGA
AGGAGATCCCATTTTGTGATTTTGTCAGGTACATTCAAGAGACAGATCAGCAAATAAAGA
GCC

Sequence prom *MAK16a C. nivariensis* reverse

AGTTCTTTTATTGGTAGTTTTTCT
AGTTCAATGTGTAAAACCAATAGAGTGATAATTGTATTCAGGATATACCAAGCTCAATAA
TATGCTAAATAAGTTAGCAACTTCGAAAAAAAAAAAAAACAACTTGCGATGAGCTCATCG
CAAAATTTTTCAGAATTTTTCAACTTGAGAAGGAAAAAGAAAAGTTACACCAAAGTTACC
CTGATATAGAATTCTCTCAGATAATTAACAATACTTTATTTTATCGTATTATACTATATA
GTATCAAAATAATATTCTCGTAAAAATTATAAATCATAGATTAATGTAAATTATGCAAAA
GTTTTTATGTCTATATTAAATTATATTATTATTATTACTGTTTTAACTATCTGGCGTATT
CAGAAGTTAAGAACTTGATTTCGTCTTGTGATAGAGAGCTGATGTACACACATTTACTCA
ATAGTTCCTGATCCGACTGTATCTTGTAGTATTTGGACCATTGTACCTTCTGTATGAAAT
TCTTTACAATTTGAACATTTGTGTTAAACTTGCTATAGTTAACAACCTTTTTTTCTGTAA
TCCAAGTCTTCTTCTCTGCGTTCAGAGATAAATCAGACAAATAAACAACAATGAAGGGGA

Sequence prom *MAK16a N. delphensis* direct

TATTGTGGTTTACTTATCTGACCTATCTATAAATGCTGAGAAAAGAACCTGGATAACACC
AAAAAAGGTTGTAAACTACAGCAAATTCAACACAAACGTGCAAATTGTCAAGAGTTTTAT
ACAAAAAGTGCAATGGTCGAAATATTATAAAATAGAATCAGATCAAGAACTCTTGAGTAA
ATGTGTCTATATTAGCTCTCTATCCCAAGATGAAATAAGGTTTCTAACATCCGAGTTTGC
CAGATGAAGTTGTATTTATAGTTTTCCAAAAGACATTCATTTCAAAAATTACATTCCCTT
AATAATAGCATATAGAAATAGCATAGTATAATAGATTAGAACATGGTCTTATTATGATTT
AGTTGATAGGCTACTTTTTGAAGGCGGGGTAACGTGATCTAGTTTGCTTGATTTCCGTTT
CTTTTTTGTTTCGTTATTTAATTTTTTAATTGAAAAATATGAAAAATTTTTGCGATGAGC
TCATCGCCTGTTGAATTATTCCACATTTTAAATGGCTACAGTTTATAATATGCAAATATT
GAATATTTTAGACCTGATCCTTTGTAAAAATAACCATTTTTGGAATACAAACACGCTGAA
ATTGTTCTGAGTT

Sequence prom *MAK16a C. bracarensis* reverse

ATCTTGCAATATATTTGGCCAACACCTGATTTACT
TGATTCACGAAATAAATAAAAAGCTAATAGGTTGAAAAAGTGTTTAAGAATAAAATTATT
ACTAAAAATGCTGCTAAATATCTTAATCGTTAACATCTATAAACTAGAACAAATAACAAA
AATAAAATATTCCAATGCGATGAGCTCATCGCAAAAATTTTCAGAATTTTTCAGGCTCAA
TAAAAAAAGAAAAGATGTAAATATGTCGGGAAAATATCGTTACCCTGTGTAGGCATGTGC
AAAGGTCTCTAAATTCAGAGCGTTACCAGATGGGTAGTATTTATATGGGAAGCCATATGT
ATGATGATATTATATGCATATGCGTTTATGATTAATTATGGGAAAGATAACTCTCTGTAT
ATATTAAATAAAATTATAACGGTAATAGAAAAATGCGATGCGGGTTTTATCTGGCAAACT
CGGAGGTTAGGAATTTAATTTCGTCCTGCGACAGAGAGCTGATATAAACACATTTGCTCA
ATAGTTCTTGATCTGATTCTATTTTATAGTATTTCGACCATTGGACCTTCTGTATGAAGT
TCTTTACAATTTGGACATTGGTGTTGAATTTGCTGTAGTTCACTACTTTCTTCTCAGTAA

Sequence prom *MAK16b C. bracarensis* direct

TTTTTAATTAATTATTACCCGGATAACACATGTGAAAGATACTCTAAGACGAAATATTCC
AAAATAGTTACCCGGCGTTACGTGAATCACAAAAAAATAT**GCACCC**GACTTTTTTGATTTT
TTTATTTATTTTTCAATTTTCTGAGCCGTCTTTTCAGGCAACTTTTTGAA**GGGTGC**AGTC
CTTTCTTCTTTCTTTCTTTTTTTTTTTGTTTTTCTTGATATTGAGTATTGTGGGATGCGA
TGAGCTTTATATTTTCTTTTTTTTTATTCATTTTTAATTAAAGGTTTTTTGAGATGATTT

CATTTATTAATTAATAAGCTTGTTAAAGATCAG**ACACCC**ATTTTTTTAAAGTTCATTTCC
TAGGTATTCCAGGCTTAATATACAGAATATTCTATTCTAGTAAAAAGGAAAGTAAAGAAA
AATTTTCTTGGTTTTGATTTACTTTTTTTAATATATAATATCGATATATAAATATATATC
ATCATTAAAGATAGATGAGAGCATACATTTATCTAAGTATTAGTATATTGTACTTAATAT
TTTTCTAAAGTATAATTAGTATATAACGTTAAAGAGGATAAGGACCAAAAAATAAAATAA
AATAAAATAAAATAAAATAAAATAAAAAGG

Sequence prom *MAK16b N. delphensis* reverse

GGTTATATATATACTACTATCTTTTTTTTTTTTCC
TACAAAAGTCGAAAACCTAAGAGTACAAGGGGAGAGCTTTTTGAATATAAACCCTCATAT
GTATATTTATACTTCTTCAGATGCTATAAACAGGAGGGAATGGGG**GGGTGT**CTGCAATTA
ATAATCAAAATCAACATTTTAGTCTTTATCAACTACTATTCTTGAGATGAGCTCATCGTC
AATTTTTTTCACTTTTTTTTTCACTTTTCATTGT**GCACCC**AAAGCCGCAGCCTCTCCGCA
CAGAA**ACACCC**GCACATACTCATTTTTTTTTTAAAAT**GGGTGT**ATGTACGTTTTTGTGCG
GCTGGTTCTGTGCGTGTTAAATTTTTCCGGGTAAACTGAAATTTTTCCTAAAAATTCAAA
AAATTGTGAATTTACAACTGATTTATTGATATTTGCATTGTATATATTGATAAAGAATGT
CATAATAATTGAGTTTTACTATACTTTTACTGGCTTCAAACGAGGAATTAGGTATTTGCC
AAATTAAAAGAAGAAGAAAAAAAATTGCTACTGTTCCTTGCTTTTTTTTTTTTTGAAGGC
TAAAAAAGTTTTGGATGCTAGGTTTTCAGAAACTGACCGG

Sequence prom *MAK16b C. nivariensis* direct

AGCACGAGTTATTGTTAAAACAAAAAAGGGTGGTTTAATGCAATGTCTTTTAATGATCTG
TCTTTGATCAAAGATTTCATTAATGACAAAGAATGAACCCAGTCAAAAAAAATCTAAAAA
ATTCTTATTTTTAGAAAAAAAAAAAAAAAACCAAACAGAAAAACCGTTACCCTGAACTAT
GATTCACACTTACCCGGTTTTCATAAAATCAAAACGTACGTAAATATCACATGACACTGT
TTATGTAAAAGTGACAACAACCTTTGCTTTTTTCA**GGGTGC**CTCGGGGAAAAAAAATTCT
TTGGTTGGCAAAGAAATCACGGAAGATTGTCAATAAAATGAAAAAAAAAAT**GGGTGT**AAA
ATATGGAAAAAAAGAAAGAAAGAGGATTTTTTGAAGCGATGAGCTTTACTATTTGGATTT
GACTTTTAATGCTCTTTACTATCCAG**ACACCC**TTTTTAAAAGAAAGGATGAGTGAAAATT
TTTCCTTGTAATCTATATCTAGATAAGATGAGATGAGATCAAGAATATGTATATATTTCC
TTTATATTTCTGTATACTAAGTAGTCTGTAATTAGAGATACGTTGCGGGAGGGTGAAAAA
CATAGCGAAGACTCATAAAG

Sequence prom *MAK16b C. glabrata* reverse

CTTCTCAAATCCAATATTTACAATGAAGAAAACACAGCCAATTT
CTATTTTCACACAGCTAAATAACTTTCAATTCAATGCCTGGAGTTGAAAGAGCTTATTTT
ACAAATATAATAAATTAGGCTAGTATTTTTCTACTTTTACGCTGGCGTTTTACAAAAGAT
ATGATACATATTCCGCAAGTTATTCGGGTTCACTGATCTTCAACTATCCGTATATATACC
ATGTAAGTTGTATAACACATTAATCTATGGCCCAGCTATCTGTTTGCCTTTTCTAAT**GCA**
CCCATTATCAGTGAGCTCATCGCAACTTTCATTTCTTTTCAGCATTTTGCCAAAATTTTC
AGAATTTTTCAATATTTTTTCACTTTGTTATCGGGACTCCTACGGCTTGCTCTAAGATAT
TCTTTTTTTCACAATCAAG**GCACCC**TATTTCAAATAATGGCCTTTGCGATGAGTTTGAAT
GCTGTATGTCCTGTACAAGTTTATTATAAGTTTGG**GGGTGT**CTGATCAGAGTTTACTTCA
CTGCCTCCTGCTCCAAAGATAGCGCATATCAAACTTACAATAATCAGGCAGTTTCTAGTT
CTTTCCACTAGTTGATCATCCTCATTAGTAGCAGATTTCTCTATTCAGTTCCCTCGCTAT

Sequence prom *MAK16 L. meyersii* direct

ATTCTACGACTTCAAGGCAAATGAAGAGCTACTAAGCAAATGTGTCTACATTAGCTGTCT
CTCTTCTGCTGAGATTACGCGATTGAGAGAGGCATCACAGTAGCAACATTTACTGGCGCA
GGATCTTGAACTCCACAATGGGGCGTCAATATCCAGCAAGATAAACAAAAATGTGGAGTG
TGAAAGTTCATACTTATAATATACAAGGACTACATTTACAAAACCCAATGGAATACACTC
GACGTTCGATTAATCCGCACTCA**ACACCC**AAGGATTTAATTCGCAGAATTAATGGGCTAC
ATCTAAATTCAATTGCAATCACTACCCACTACAATGAATAAACACTGAACATATGAACAG

ATAAAATGACAAGCATGCTGTCATGAACTTCCCAGTGTGTCGTCAGACTGCTCATCGCTC
ATCGCTTTCTGGCATAAGGTCAGCCGTTAACGAGCTGTCTACGCATCATTGTTTATTTTC
CTGTCGTCGAGAAATCTGAAAAATTTTTGATAAAATTTATTAGAGCGATGAGATGAGATG
AGCTCTAGAAAGCTTTAACCAACGTAAACAACTGAGAGTGAAGCTTTCTTTGAATATCGA
GACAGCGTTTAAACACGGTTAACAAGCTCTACA

Sequence prom *MAK16 L. fantastica* reverse

TTCAGGTGCTTGGTTGAGCTATGAAACCCTT
CTTTGACTTCGATCAAACAAGTTCTTAGCTTACTTGTGTATTGGCGACTGATGCTCTTGA
AGCTCATCTCATCGCATCGCTTTCTGAAATTTATCAAAAATTTTTCAGCTCTTTCAACGA
AATTAAAATGAGAGAATGATAAGCTGACGTAAGCTGATCTGGAAAGATGGTATGACGTAA
GCGGCGTTAAAACTTGGAAAGCTGGACGACACGAAAGTTTCTACAATTGTGATATCCTTT
TGAATGTGCGTACCAATTGGTCTGTTAACGCAAGTAAAAGAATGGCTTGTATGCCTTTTT
ATTCCCCGTCGCTTACGGAAAAAAATCAATTTCAACCTTCAATTTCGGTAGCGTCCAAGC
GTGGAGACGCTAAAGTGTCCTTCAACTGCGTAATCTCAGCCTGCGAAAGACAGCTTATGT
ACACGCATTTGCTCAAGAGCTCGTCATTTGTTTGGATATCGTAGAATTTGGACCATTGTG
CTCTCTGAATAAAATTCTTCACAATCTGGACATTAGTCTGAAATTTGCTGTAGTTTAGGA

Sequence prom *MAK16 L. kluyveri* direct

ACAGACACTTGGAGGTTGATTGAGCCTGGTGATTTACTTACATGGGAAGAATTAAAGAAG
GTGCCGTCATTGGATGGGAACTATTCAAATGTCAGAAAGCTGTTGAACGAAATCAATCCG
ATTAAAGGTTGTATTCCATTTATTGTTGTTTATTTGTCAGACCTTTCCTTGAACGCAGAA
AAGAAGAACTGGATAATTCCCAACAAGGTGGTCAACTATAGCAAGTTTCAGACCAACGTT
CAGATCGTCAAGAACTTTATTCAGAGAGTCCAGTGGTCAAAATTCTACGATATCGAGGTT
GATAATGAATTATTGAGCAAATGCGTATACATTAGTTGCCTTACCCATGAAGAAATCAAA
CATTTGGCTACCATTAAATAAAATGACCTCTAACTATTTAGCACAGTAAGTTACCTAGAA
ACGTTATGTAACTAATAAACAATCATCATTTGGAGGATTGATATTTTATATAGTTGTCTA
TATATAGGCGGGTAACCCTCTATTCTTGGCTGGCAAGGCGTTTCTTTTTCTGAGAAAAAA
AATTTTTTTTCTGAAAAATATTTCATGTGATGAGATGAGATGAGCATTCAAATAGTGTGT
TGATATCCCGGAAGCTAATCAGATTATAACTACGGCAACG

Sequence prom *MAK16 L. mirantina* reverse

GGCAATCTATGATCGATTCGCCTGTATTCGCTGTACTGCAGCTC
TCTAAAACTACGAAATTTTTCAAGCTCATCTCATCGCATCGCCAATTCTTGAAAATTTTC
TAGCAAAAAGAAATACGGTTTTCGATATTCATGGACTTGAGTTACCCGACATCTTGAATT
TCTATGGACAACTCAACTACAACAGAAAACAGGTCTAAGGACGTCGACTTAGGCCAGGTC
TGGGCCAGCTAAT**GGGTGT**CTTTGTATCGTCTATTTGGTTTATGACTACTTAATTGAGCT
AAGCTGTGTACTATTAGTTTTGGAGAGCTGGTCGATTTCATCCGGACTCAGACAGGTGAT
ATAAACGCACTTGCTGAGAAGTTCATCATCCGCTTTGATTTGGTAGAACTTGGACCATTG
GGCTCTTAGTATGAAATTTTTGACAATCTGTATATTTGTCTGGAATTTACCATAATTCAA
GACTTTGTGAGGAACGATCCAGTCTCTTTTCTCGGCGTTGAGTGTCAGATCTGATAAATA
CACCACTAGGAAGGGAATGCATCCCGTCATTGGATTCATGTCGTTGAGCAGCACTCTCAT
GTTCCGGTAGTTTCTTTCCAACGACGGTAAATCTCTGAGGTTCTGCCACGTGAGAAGATC

Sequence prom *MAK16 L. waltii* reverse

ATTTAAATAAG
TATCAGAGAGCTATTGGTTCGTGCTACCCTGAAGGACAATACTGTCTGTTTTTGAACTAA
TGTCGGCTATTTCATCAAGCTCATCGCCTAGTCAAATTTTTTTCGCATTTTTTTTCAATA
TTGGGATGCAAGGAAAAGAAAATGTGATGAGCTAAATTTTGACATGGTAGAAGGCGATGA
GATGTGGATTCTATTAAGAATATGGCAGGAACAAAACAATTTGTTCCGAAGTTGCAGAGG
GCAGTCTTCGTAATCTAATGTTTATTAAATTTACACGGTGGTGCCATTGATAGGCCACCT
CTTGTACATCGTGGAAGTAGTTTAATACCTGCGATAAACAGTGGAGAAAGTTTCAAGGTA
CAGTTTGGGTGAGGTCCAAAGCTCGCCTGCTTGACCACGAGCCTTAGCTAGCTGGGCTAC
CTCGGACAAGGTGATTTATCTCGTTTTGGGTAAGGCAGCTTATGTATACGCATTTGCTGA

GCAGCTCCTCATTAACGCTGAACTGATAGAACTTCGACCATTGCGCTCTTTGAATAAAGT
TTTTGACAATCTGTACGTTAGTCTGGAATTTGCTGTAGTTCAATACCTGATTTGGAACAA

Sequence prom *MAK16 L. dasiensis* direct

TATAAAGGGCTGCGTTCCATTCATTGTCGTGTACCTTTCTGACTTGACACTTAACTCCGA
GAAGAACAATTGGATATCACAGGATCAAGTGCTGAATTACAGTAAGTTCCAAACAAACGT
TCAAATAGTCAAAAATTTCATACAACGCGCTCAGTGGTCGAAATTTTATGACTTCAAGAT
AGAGGAGGAATTGTTAAGCAAATGCGTTTACATAAGTTGTCTTTCACAAGATGAAATAGC
ACATGTTACGCAAAATTTGCTTTAATCACCACATATGCGACTTGAGGCCATCAGAAGTTA
GTCGCCTAGAGTTTTGGTAGCCATAATATGCTCGTTTTGAAATATACTTCAAATGCTCAA
TATATTCTGGCCGCTTGACTCACAGCTGATAAAATATCGCGTTTTCCACATTTGCATCAT
CTCATCTCATCGCATCGTTCTACTTTATCATTTTGTTTTTCACTGTCGTCCATAAATTTG
AAAAATTTTTGTTAAAAAGCAGCGATCCGATGAGATGAGCTCACTTAACATTATTTGAGC
GACTAAAACTGAGATAGTCCAAGAACAAAAGGAAATCCTCATTAGTCTCTCGTTGTTGTG
TTTTATAAGAG

Sequence prom *MAK16 N. castellii* reverse

GGTATATGCGATTGTCGATATGATATCAAAAGGTCCTACTGCACTAA
TCAATTAATCAATCACTTGTAACATACTAAAACTAAGCTCATCGCAGAAAATTTTTCACT
TTTCCAGTAAGTTGGTAGTGTCCGGAAAACCCCCGGTATGGTCACGGACACGGCATGACT
GGCTCGAAATTTGAAATTCGAAATTTCAGAGACAATGACAAAATAGGATTTCAGTGAATA
ACTGAGGTGTCGTGAACAAGGCAGTGAGTGAGTGATTGAGTCACTGGAACCATATTTGTC
TCTTTACTTTGCGTTATAATTAAGTGCAGATTGCCGCTTTTTATCCTTTGTATCGAGGAT
ACCACCAGTTTAACAAAGATAACTGATAGCGAAAAATGCTACCGTTTGCCAAATTAGCAC
AGTCTTCTAGCATAAAGTCGCTGAATCCATCGAAAAATGTTGCTACAGAGCATCCAATAG
AATTGAGCATTAATGTGGAATCGCCTCCCTGCGTACTTTATGGATCAGCAGCGGATTCCA
CGGGGTCATTATTAAGCGGTGTTGTCACATTGAATGTCAAGGATCCATATGCTGATGTAT
CCATACCGACTTATGATTCCATCAAACCTTCCAAAAGTGCCCCTCAGAAGTCCCCAAGAC

Sequence prom *MAK16 L. nothofagi* reverse

TGATAGGTACTGTTATATCAA
ATAGTTCAATAGATGGGTTCTTCAAATGACTTTCTATAATTCTAATTGCCTTAGACTTTA
GTCGGCTAAATATCGATTTTGAAGCTCATCTCATCGCATCGCAGAAATTTTTCAAAAAAT
TTTCAACTTTTAGCGACGGGTAAAAGAAAAGCGAGAGGTAGAGACAGACAAACAGAGCTG
TAATTATGTAGCATTTCGATGAGAACAGAGAAGAACTCAGCTTCAACATAACCCAGTATT
AAAGCAGGAATCGGCATTCTGTGAATTTCTATTTGAGTGGTTGAACTATGCGAATAACTC
ATTGCTCAGAATGCATCAGGTGTTAATTTGTTATCTATCATAATTGATTTTCTATTTCTT
AATATTACAGGATTTCTTCGAAAGAATGTCTCCGGGCTTTGACGGTGACCTACTGTTTTG
TTTCTCTCAAGTGAGAAATCTCTTCTTGGGAAAGGCAGCTTATGTAAACGCATTTGCTCA
ACAACTCCTCGTTGATCTTGATAGTGTAGAATTTCGACCACTGAGCTCGCTGTATGAAGT
TCTTCACAATTTGAACATTGGTCTGGAACTTGCTGTAGTTTAAAACTTGCTTCATAACAA

Sequence prom *MAK16 K. lactis* reverse

CTGCTAATGTT
CAGTCAATCCTACAGATACTGGTTTATGCTTAGTATGAGTTTATTTAAATTGGTTATGAC
TATCGCTAAGTTTACTTGCTATTCCGCTAGTCGCTGCTTCTTTCATTTCATCTCATCTCA
TCGCATCGCATCGATATTTTTCAGTTCTCAAAAATTTTCAGGCAGAAAGAGAAAAAAGAA
ATGGATTACTAAATTTTTAGTCACATGACCAATGATATTTTATTGTTATAATCGGAAACT
TAATCAAGAGTCGGAATATGCATGATTAGATCTAAAGAGATTAACCTTCATTCAAATATG
TTCGAAAAAAATGTGGATGAAACTATTTGAAACTGATAAAATCGTGTGTTGATATATATA
TTATATGAGGTGAAATGAACTGAGACTAACTAGTTCTATGTCAAATACTTTACTCGACAT
TAAAGAATTAAATTGGGGATTTATGTGAAATTGAATTGATTTCGTCATGGGACAATGATG
TGATGTAAACACACTTGCTTAACAACTCATGGTCAATATCAATGTTGTAAAATTTGGACC

ATTGCATCTTCTGTACGAAGTTTTTCACTATTTGAACATTTGTTTGGAATTTGTTGTAAT

Sequence prom *MAK16 L. cidri* direct

CTCTATTGTCGTTCAAAGCTTCAGAGATGCATGGAGGCTGATTGAACCAGGCGATCTCTT
AACGTGGGGCCAACTTAAGAACATTCCATCTTTGGAAAAAAACTACTACAATGTCAGAAA
GATGCTGAATGAAATCGACCCAATTAAGGGATGCATTCCTTTTCTCGTTGTCTATTTGTC
AGACCTATCGTTGAACAGTGAAAAAAGGGACTGGATTGTCAGTCAAGACATTCTAAACTA
CAGCAAATTTCAAACGAATGTTGAAATTGTCAAAAATTTCATTCAAAGGGCAGAATGGTC
AAAATTCTACGAAATTGACGTCAATGAAGAGTTGTTGAGTAAGTGTGTTTACATCAGCTC
CCTTGCCCCAGAAGAATTGAGGCACCTTACCACTTCATAGTAGATATATTATCTAAACTA
ATTGCATATATTGTTGTTATAGAACATGCCATTAACTTCTTACACGTGATCCCTGTCTGA
CACATTTCTTCAGAATCTAATTGTTGAATCTACTACGATTATTGAACAATCAATCATATA
TCCGGGTAAGTTTATGTTTCTTTTTCACTTTCTGAAAATTTTTTTGTTAGAATTAAAGC
GATGAGATGAGATGAGCTTAGTTATTTTGAAAGAAACGTACCGATAACGATCTAGA

Sequence prom *MAK16 L. fermentati* direct

ACAAAGCTTCAAGGAGGCGTGGCGTTTGATAGAACCCGGTGACCTTCTGACTTGGGAGCA
ATTAAAAGAGATGCCCTCTTTAGAAAAAAACTATCACAACATTAGGAAAATGCTAAATGA
AATTGATCCGATAAAAGGATGCATTCCTTTCATTGTTGTGTACCTGTCAGATCTTTCCTT
GAACGCTGAAAAAAGGGATTGGCTTGCCGGGAGGACCTTTATCAACTACAATAAATTTCA
AACCAACGTGGACATTGTCAAAAATTTCATTCAAAGAGCTCAATGGTCTAAGTTTTACGA
GATCCCGGTCGATGAAGAGTTATTGAGCAAGTGTGTTTATATAAGCTCTCTCACGCCAGA
AGAATTGAGACAATTGACTACTTCGTGATATTTCAGATACATATAAACCTGCATAAACTG
TGTTATACATCTTAAAAATAATTTAAAGTCAATAGTATTTGAACACTCGATGTCCATGGT
CAGTCCATCAAAATTTAAACTAGAACACGTAAATAACAAAGCCGGGTAATTAAATATTTC
TTTTTCACTGTTTGAAAATTTTTTTGTAAATTTCAGCGATGCGATGAGATGAGCTCCACA
GCATCGTTGGGATCCGCTATTGGAATTAATTAGAA

Sequence prom *MAK16 Z. rouxii* reverse

CCTTATTCCAAAGA
GGTACAATCAAGTATTCAGATATCCGCCAACTGTTATCTTGTTGGGAGTAATTGTAATTG
TTATAGTAGTGAGATAGTAGCTCATCTCATCTCATCTCATCTCATCTCATCGCTTCGAAA
ATTTTTCACTTGAAAAATTTCCGAGACAGCGACAGCGCCAGCGACAGCGTCAGTAAATAA
ATAAATAAAAATATAAAATAGAATAAAGTTAAATTAAATAAGAAGTAAAAAAAAATGTTC
TCAGCGGCTATCAAATTACCTTCATAAATATTTTTTTTTTCTTCTTTTTTTTTTTATTGAA
AGCAGAAAAATCGGGAGAAAATGCATAATTTGTTATATAAGAGTTTGTTATTTGTTTAGT
TTGTTTATGATCTGGGTGATCTCATCGTGTGACAGTGCTGTCAAATAAACACATTTACTC
AGCAGTTCATGATCTACAGGGAAGTTGTAAAATTTGGACCATTGAACCCTTTGAATGAAG
TTTTTCACAATTTGTACGTTCGTTTCAAATTTGTTGTAATTAACAACTTGTTTTTCTTCG
ATCCAGGTCCTCTTCTCATTATTCAAGGTCAGATCAGATAGATAAACCACTATGAAAGGT

Sequence prom *MAK16 L. thermotolerans* reverse

TATTGAATAGA
TATCAGAAGTTCGTCCTTTTGGCGTATCTAGTCAATGTTTATTGCGATACTTCTTGTGAT
AACTATACACTTCAAGCTCATCGCGCTCATCGCATCTCAAATTTTTTTTACAAATTTTTC
AGACTTGAAAATGGAAGGAAAAGAAAATGCTGCCATAATACAAGATGCGCTAAGGCATCG
CGAAGTTGCAGATTAGAAGAATTAACAGGGAAACAAATTACAACGAAGGTAATATATTAC
AGAGTTGGGGTTACGTACTTGATAAAATGAAATGCATAGATCGATTCAATGAAACTCTTG
AATAACATTGAGAATGTCAGAAATCTGTTATGGGGTTGTGCGCTTTGCAAAGCTTTTCAA
ATGAACATGGCAGTAAAGTTCAGCTCCTCTATGTTGCGCTTTTGGTTTGCTGACTTTTCA
AATGGTCTATCTCTTCATGTGTTAGGCAACTGATGTAAACGCACTTGCTCAGTAATTCAT
CATTAACACTAATATTGTAAAACTTTGACCATTGAGCCCTTTGGATAAAGTTCTTAACAA
TCTGAACATTTGTTTGAAATTTACTGTAGTTTAGGACTTGATGTGGGGTAATCCAATTGC

Sequence prom *MAK16 T. blattae* direct

AATTATAACAAATTTGAGACTAGTGTTCAAATTGTCAAAAATTTTATTCAGCATGTTCAA
TGGGGTAAATTCTATGATTTTAAAGTAAACCACGATCTACTAAGCAAATGTGTCTATATA
TCTATTCTTACTCGTGATGAAATTAACCAACTGTCAAGTTAATGTAACTTTATTATAACT
GAAAAAATTATTTCAACAAGCCCACTTATTGTTATTTTAAGAAAAGACTATTGTTTTATT
TTAAAAGGACTTTTATATTCTACATAAAGAACTAATATTTAATAAATAATCATATAAATA
ATTTTAATAATATTTTGCTTTCATATGTGTCACAGTATTGAGTTCATCATAAGAGAATTG
AAGTGAAAAAGGTGTAAGGTTCAAAATATTACTAAAAAGCCTGTTGGCAGCAGATTTCTC
AAATTTCTTATGACCGGGTAAGGCATCGGAATTTTTCAAAAAAATTTTCATATCTTAAAA
AAACGATGAGATAGAGACATTTATGCAATAGTTAAAAAATATTCAGAAAAAAGGATACTT
GCTAATTTTTTCATTTGTATTGATTAAATAATCAGCTTTATAGTCTATATATCGTTATAG
AGTTCATTCTGGTTATTAAAAAAGCAAT

Sequence prom *MAK16 E. gossypii* direct

GCGGTAGCCTCTCTATAAACCTCGCGTAGTATGTCTTCTCGTCGATCGCCAGGCGGTACG
TCCTCACCTCCTCAGTGGCAGTTCCAAAGTACGGGATTGTCCTGAAGCACAGCAGAGGCG
CTGCCCTCTCTTGTGCGGAAGGTTCTATACCTTCGTACTGCGCGAATATCCGTAGTAGCA
CCAGGCGCTCGGCTGAGGTAGCGCCTGAGAGG**ACACCC**TCAAGCATTGCCAAGTCCGTGA
CTATCCTGCTCATTCGAACACTATCCCGAGATGACGTCCTATACCCTACTCGTGTATCTT
TTTGGCCACCAGGGTCGCCGCAAGGCCACAAACTATACAAAGAACATATCGGGCTTTCAC
ACGGGACGGCGAGGGTTGATTTACCAGTGCGCGGGTTTGACGCACACTCCATGCAGAGGT
CTTAACTCGGCGTCTCTTCAGCTACAGTGGCAGATACATGAGAGTCCGGGAGAGCCACAT
TGCGGCGCGGGAACCAGCGCAAGAGGAGCATCCGCACTGTGTCGTAGATCTTCAATCGGC
GGAGCCCGGGCGGGCTCGAGCGCTGGCTGATTAAATAAGCATCACGTGACCGTTTCTTTT
TTGAAAATTTTTCAACATTAGGAACTATAGATGCG

Sequence prom *MAK16 C. castellii* direct

ATCGGACCAATGTCAGGGTAGTTAAGAGTTTTGTGCAGCGCGTTCAATGGGCGAAATTCT
ACAGAATCAAAGTAGATCATGAACTACTCAGTAAATGTGTTTACTTGACATGTTTAACGC
CAAGAGAGATCGATATTTTGCTGGGTTATAGTATGTGAGTTGTATCAGGATGCAGCTTAT
CGACCCAAACCCCCTGTTTATTTTACAGGGTTAATGACAGTACTTTATAAATATTACAAA
CGTAAATAATTCACGACGTTCCGTACTAATAGACATTTGCTCAATTTTTATCAACTAAGA
AAGCGACTACATACAACAGCATGTACCGGTCAACATAAAATCACCCAACTCTTGACGATT
CATCGATTCCAGACACCTCTCAATCCATAGATAATATATGGTTAAGTATATGGATGGAAT
GAATGTCCGGGTACTCTGACATGAATTTCAAGTTTTAATATTTTTCATCATTTATAGATC
TTGAAAAATTTTTTGATGGGCATCGCATCGCATCGCGCTTAGACATCATAATTCATACAG
TCCTATTTAGCACCAATTGACAGACAGACTAAAGCATTATACCCAGATAAGGTTGAAGTA
TATATCA

Sequence prom *MAK16 N. bacillisporus* reverse

TTCTGTATTTCTGCACTGATATTTA
TCACTATGGTTTAATAATAACACAAATAAACTTGAATCCTGAACGGTATTCAACTGTGCT
TAGAATAAATCAATTATATAATTGCAGGTTATATTGTTATTGAAGCTCATCGGAAAATTT
TTTTCACTTATTAATTTTTTATTTTTTCAAGCTCATCGCTAATTGATGAAAAATTGTATA
ACAATCAAATAGACTTAAAGAGGAATAATAGCCTGTCAAGTGACTAAAGTATGCATTAAT
TATATATAATTATTTTTAAATATAAGTTTATAAATGTGAAGTATTTTCAAAGTCTCTTTG
CTTAGACTACCAAAGAGTCAATCTCTGTTGGTGTCAAGCATGATATATAAACACATTTGC
TTAAAAGTTCATGATTTACCTCAAAATTGTAGAATTTGGTCCATTGCACTCTCTGAATAA
AGTTCTTTACAATTTTAGCACTAGTTGCAAATTTATGGAAGTTGATTATCCCATTGTCCC
TGGAGATCCAGGTATTTTTTTGGTTGTTTAACGAAAGATCCGATAAGTATAATACAATAA
ATGGGACACATCCAACAGCTGGATCACTTTCATTGAGTAATCCTCTGATGTTTGCATAAT

Sequence prom *MAK16 Z. balii* direct

TGAACTAAAGAAAATGCCATCCCTTGACAAAAACTACGCATATATCAGGAAACTACTCAA
TAATATCGACCCAATGAAAGGCTGT**GCACCC**TTTATCGTGGTGTACCTTTCTGATCTCTC
ATTAAACTCCGAGAAAAGAACTTGGATAAAGGACAATGAGATTGTTAATTATAACAAGTT
TGAAACTAATGTGCAAATTGTTAAGAATTTTATCCAAAGAGTTCAATGGTCGAAATTCTA
TGACTTTCGCGTTGATCATGAATTGTTAAGCAAATGTGTCTATCTAACCGCTTTGTCACA
CACCGAGATGACACAAGTCATCAATAGATCAACAAATAATAAATAACCACTGTGTAACAA
ATAATGCATTATAAGTAATATGGATTTTTTTTAATTTTAATTCGTTAATTTCATCTTCAC
CTTTTTTTTTTTTTTTTTTTTTTTTTTTTTTTTTTTTTTTTTTTTTTNTTCACTTTCGTT
CGTTTCTCAGTGAGATGAAAATGAAAAATTTTTGAAGCGATGAGATGAGATGCGATGAGC
TTATTATACTCTACTTATAGATTGCAACGTTGAAAAATAATTGGTTCTTTCACCTGTCAA
ACGTTCACGGGCTAGAAAAGGAA

Sequence prom *MAK16 K. africana* reverse

CTCTCAATTCTATTTCAATCACACACCTCTGCCTTACTAAAA
CTCTTGCTTCATATATAGTTAAAGCTCATCTCATCCACTAAAAATTTTTCAGCATATCAAAAAAAAAAAA
AAAAGAAAAATTTTCGTAACATCCACACATCAAAAAAAATACAGAAAGTATGAAAAATTGAATATTTAGA
CTTTAAAGAGGTGTGTGAGTCAGCTTAGACAGAGTTAGAAGTTATATATACTAATAAGTCTAGTGTACGG
CAACGGTAAGACTGCTGGATAGTTTTACCCGAGAAAGACAGTTTATTGTACATTAATTTAGCTAGTTTCA
TAGCATCTAGCATTCGTTTCTGTAAGAACAGGTATCACTGGTACGGCTGGACTACCTTAAATTCTCATCA
TACCAGAAGCGTTTCCGTGTATAAATGGTACTGAAAAAGCTTGCACCTACTACATCTGCTCCCGGTATGA
GGACCCATTCGGGCAATAATAACGATGACCTGTCTTTACGGCAAAGGATGGACTCTCCCATCGCTTTATC
TTTGAGCGTAGAATCTCCTCCATGTGTCATGTATGGGCCTGCTATCGATTCACCAGGTGCTATCTTATC

Supplementary file S9: Expression profiles (Log2(selenite/mock treatment expression ratio)) as a function of time (1 unit = 10 minutes) for genes of the conserved Environmental Stress Response, as defined by Roy et al., Genome Research 2013. Left panel: genes of the Ribosome Biogenesis (RiBi) regulon. Right panel: genes involved in proteasome biogenesis and functioning.

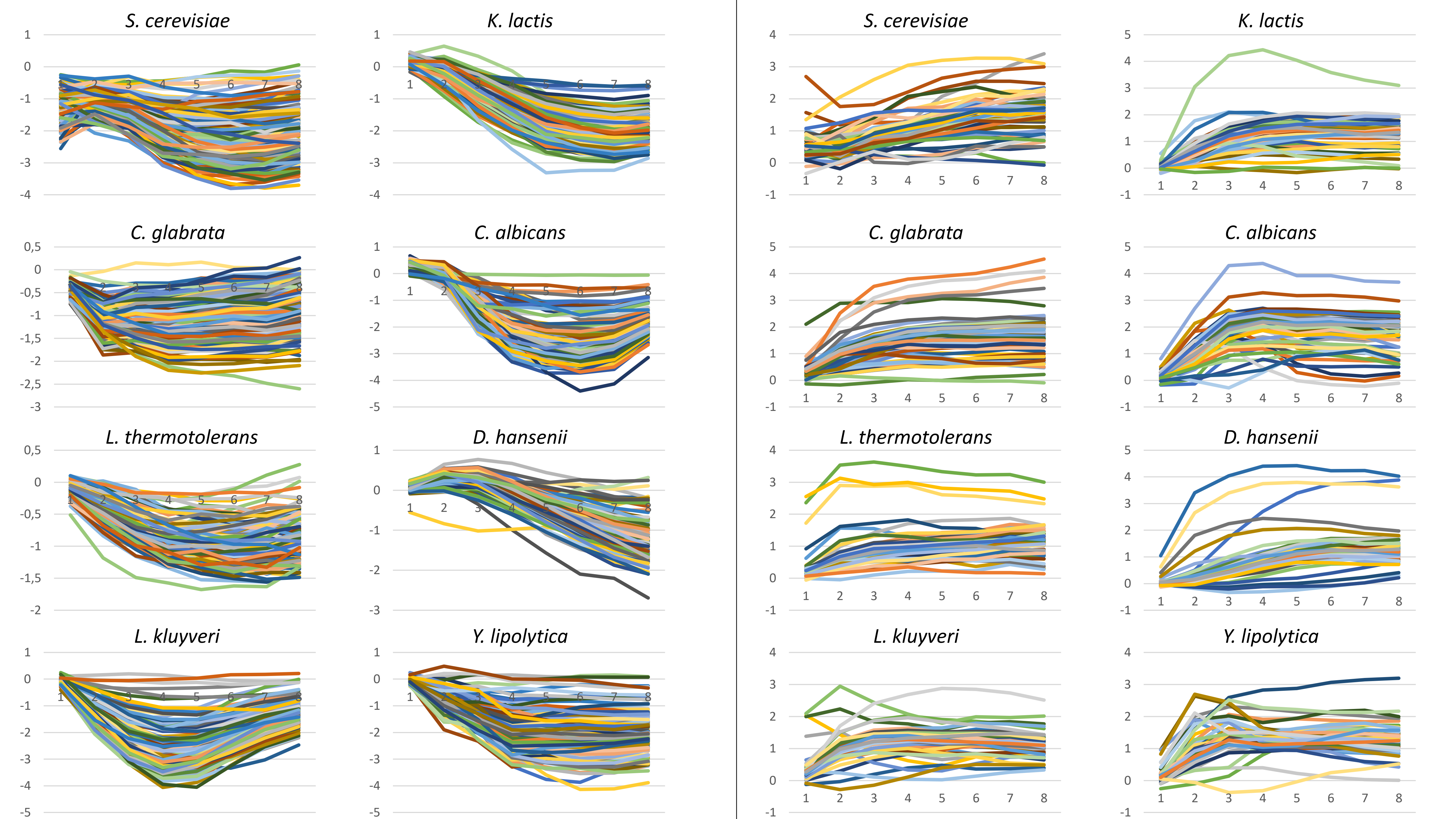

S. cerevisiae
K. lactis
S. cerevisiae
K. lactis
C. glabrata
C. albicans
C. glabrata
C. albicans
L. thermotolerans
D. hansenii
L. thermotolerans
D. hansenii
L. kluyveri
Y. lipolytica
L. kluyveri
Y. lipolytica

S. cerevisiae	*C. glabrata*	*L. kluyveri*	*L. thermotolerans*	*K. lactis*	*D. hansenii*	*C. albicans*	*Y. lipolytica*
62	38	11	6	15	17	87	53
NS	alpha amino acid biosynthetic process (p-value = 1.82 e-05) Ribosome disassembly/no go decay (p-value = 0.033)	Glycine catabolic process (p-value = 0.0008)	NS	NS	NS	arginine biosynthetic process (p-value = 2.13 e-07) Carboxylic acid metabolic process (p-value = 5.08 e-07)	NS

Supplementary fileS10: Gene Ontology analyses of the regulatory outliers for each species. The main categories and the corresponding p-values are indicated. NS= Nothing Significant.

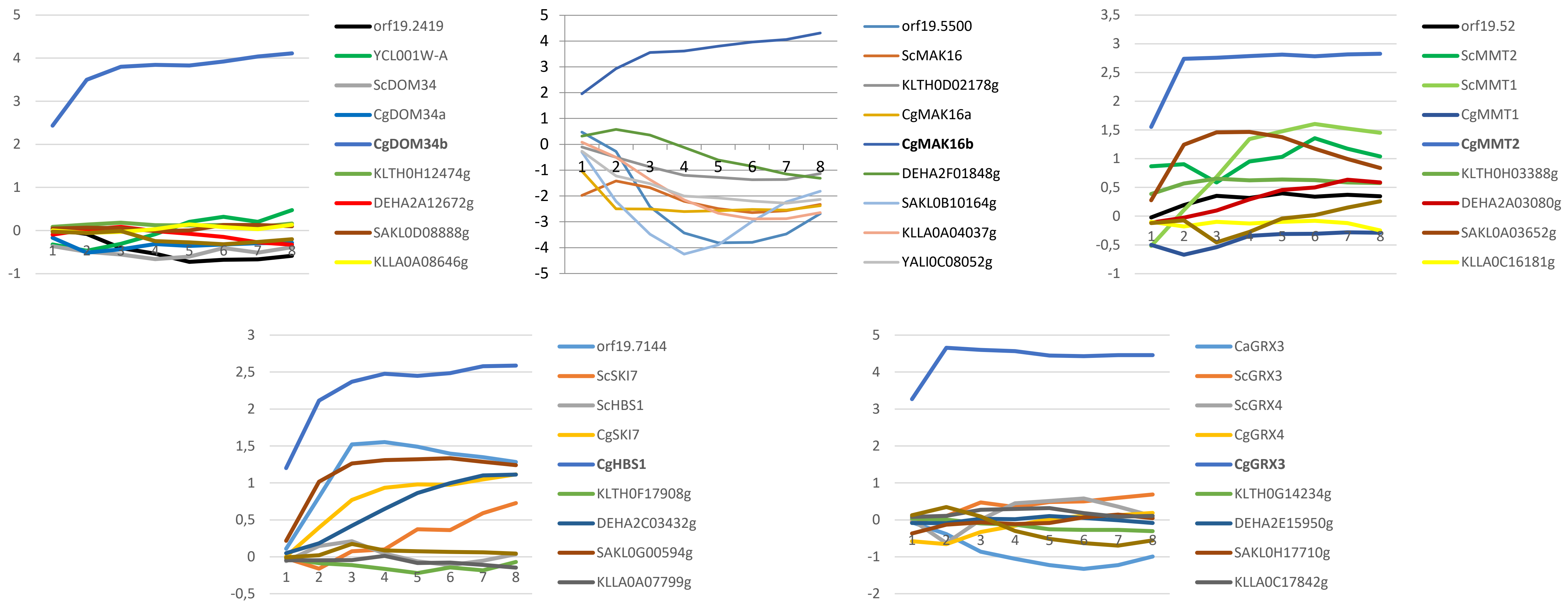

Supplementary file S11: Expression profiles (Log2(selenite/mock treatment expression ratio)) as a function of time (1 unit = 10 minutes) for the orthogroups *DOM34*, *HBS1/SKI7*, *GRX3/GRX4*, *MMT1/MMT2* and *MAK16*. The names of the genes in the orthogroup are indicated at the right of each graph. The names of the regulatory outliers in each group are in bold.

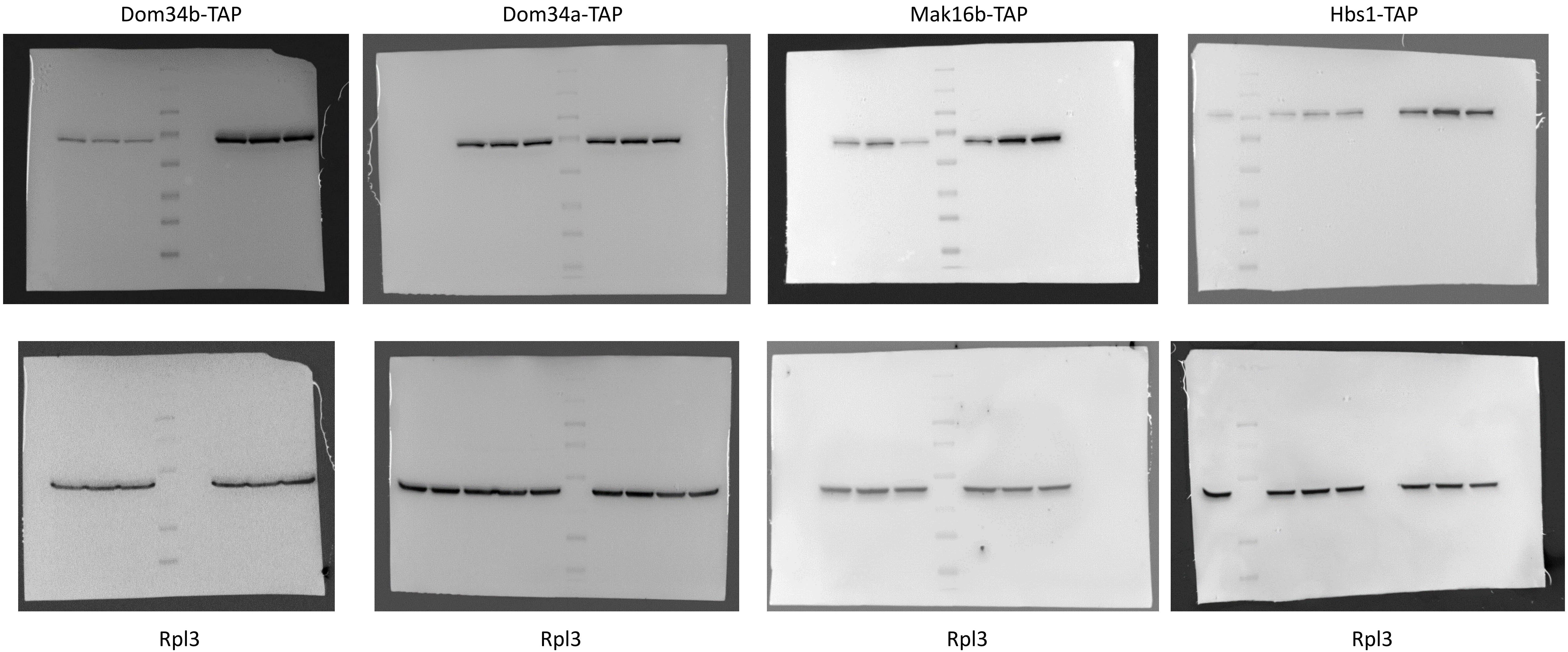

Supplementary file S12: complete gel pictures for the western blots presented in figure 4. Upper part: TAP signal. Lower part: Rpl3 signal.

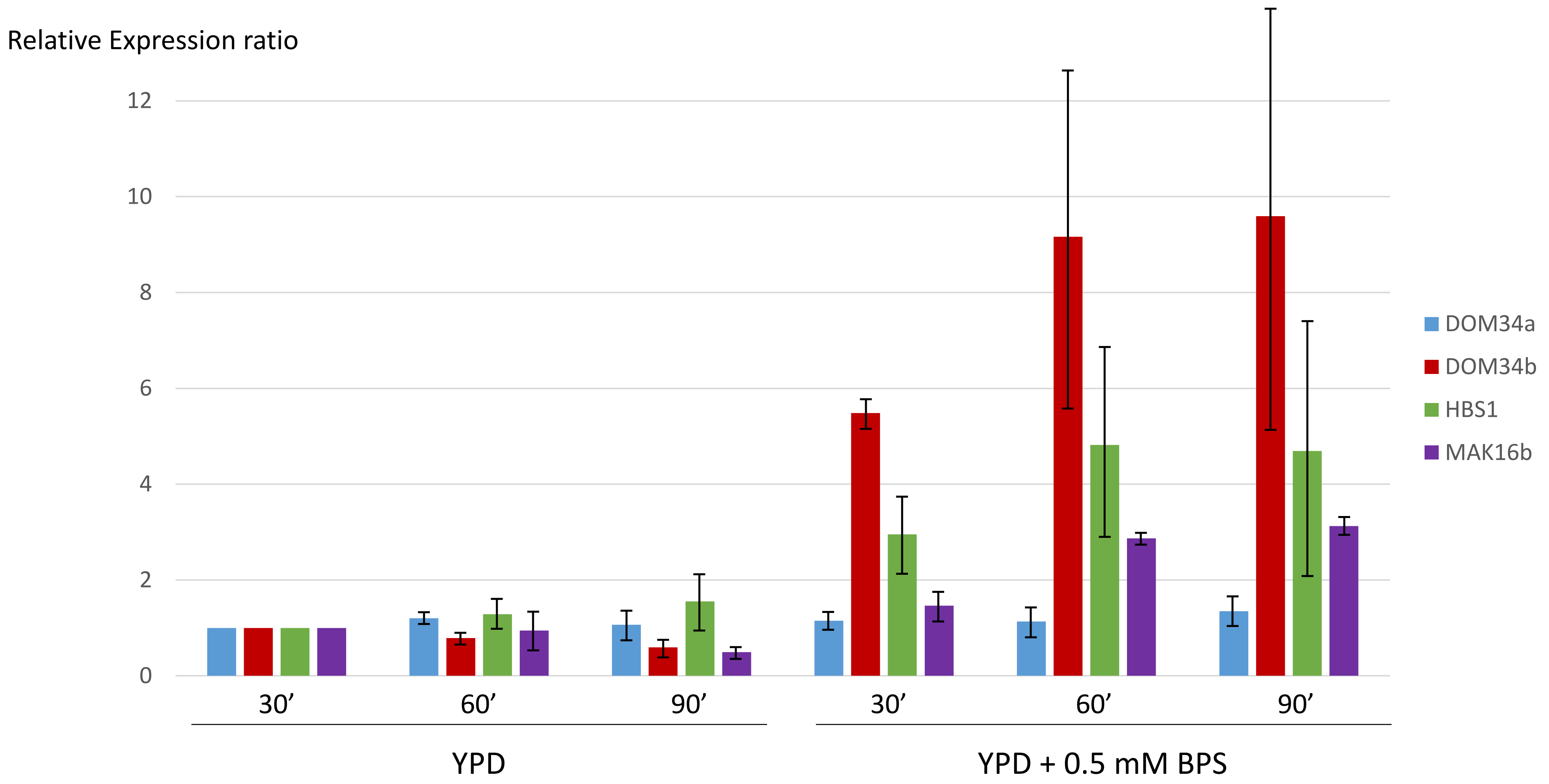

Supplementary file S13: Quantification of western blot analyses. The signal of the western blots were quantified using ImageJ. The signals obtained for time 30’ of the mock treatment condition (YPD) was used as a reference. All signals were normalized using the corresponding Rpl3 signal as a loading control. Two biologically independent experiments were quantified. The values represented here are the averages of the two. Then, the error bars represent the deviation of the two measurements.

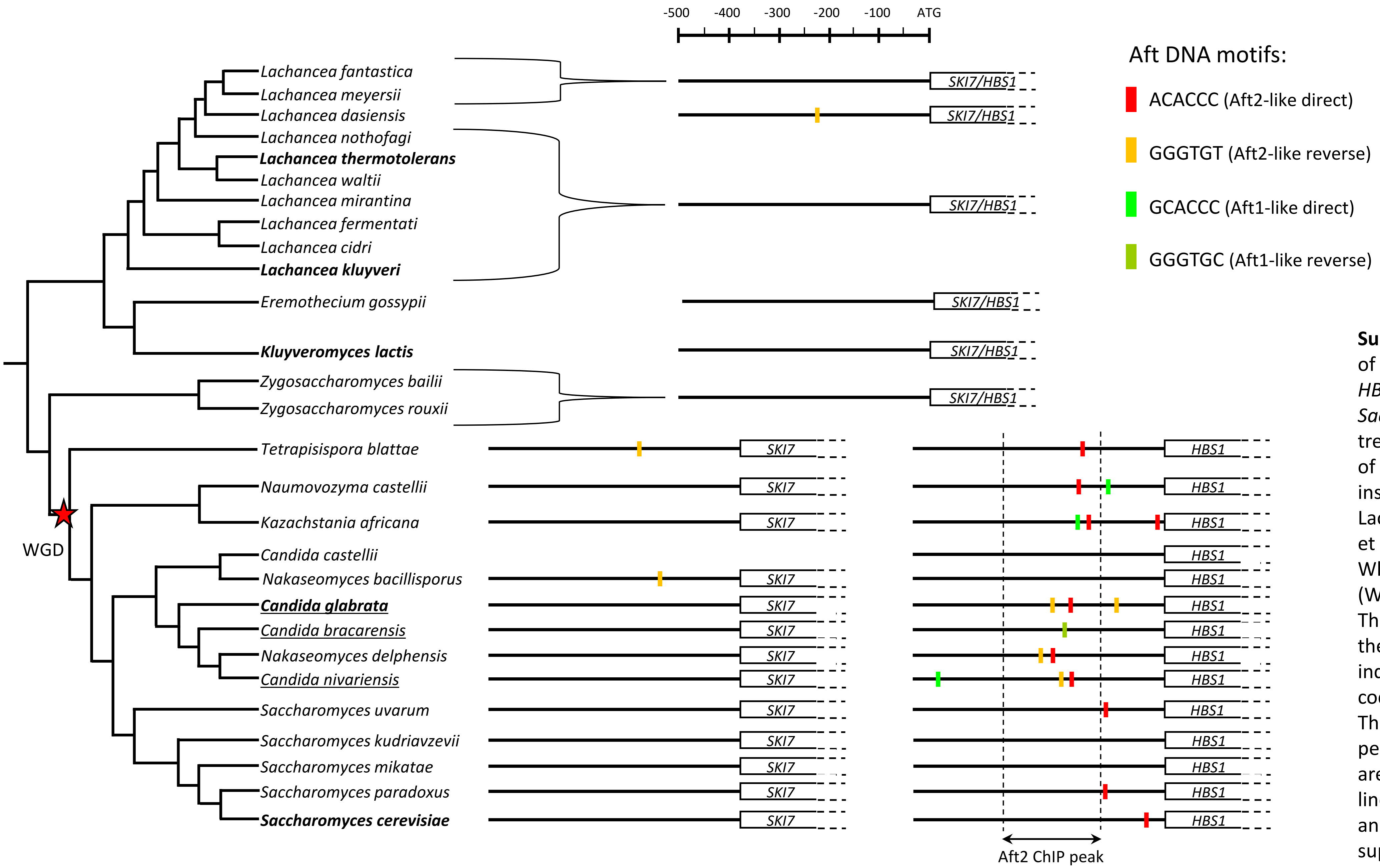

Supplementary file S14: Analyses of the promoters of the *HBS1/SKI7* orthogroup in 28 *Saccharomycetaceae* species. The tree is a schematic representation of the phylogeny of the species inspired from Vakirlis et al. for the Lachancea species and from Shen et al. for all the other species. The Whole Genome Duplication (WGD) is indicated by a red star. The positions of the Aft sites in the promoters of the genes is indicated by colored boxes (color code is on the upper right part). The boundaries of the Aft2 ChIP peak in the *C. glabrata HBS1* gene are indicated by dashed vertical lines. The sequences used for this analysis can be found in supplementary file S6.

```
C.bracarensis       ---------------------CGTAAGTCTAC-CGATTAAGGAAA------ATGGGTGTC
N.delphensis        ----TTAGATGGCTAACTTTTTCACTTCTAG-TCAATAAGCAGAT-----GGGGGTGTC
C.niviariensis      CGGAGTACCAGATTAAACATTTTGATTTCCACACCGATGAGATGGTCATTGAGGGGTGTC
C.glabrata          -----------------GAAGGCTACCACTAT-TCAATATGCGAAC-ACTCGCAGTTTTC
                                            *   * *      *  *             * * **

C.bracarensis       TGCCATAGAAATATA---GAGAAAAAAGAAATTTAGGAAAG--CAGTTCAAATGGAAGAG
N.delphensis        TAAAGAATATCCTCGTCTGGGTGTCTGACTATTTATTTTAG--TAGCT--ATTAAGAGAG
C.niviariensis      TGGCTCAAGGGTGCAAGGAAGAAAACAGAAATG-ATCTTTT--TGATTTTATTTAA----
C.glabrata          GTAATCAAAAATCCATCGATACAATAACTAGTATACTTTGGAACAATAACATTTACTGA-
                          *                        *  *               * *

C.bracarensis       CATTAGTAATCTTAATCCACTT--TTTAAAACTCCATGAAGCAAGGTTATTAGGTGAGGT
N.delphensis        TTTAAGTGAGTTTAGCTTGCATGCTCTAGAGCATGATGCGATGGGGT-GTGGGACTACCA
C.niviariensis      --TCAAAAACTGTTTGTTACTGAAATTGTTGGATACTGCGATGGGGT-GCAAGA----AT
C.glabrata          -TCAACCCAACTACCGCAGCATAAACTACTTCATCTTGTGTTCGAATTTTGAGTATCGTG
                        *   *          *      *         **        *     *

C.bracarensis       GCATTTGCACAACTCGTACCTTTGTATCGTTCCTTTCTTTTTTTCTAACATATAAGTCAA
N.delphensis        TCATCGATTTGAACAATATCAATTTTTTATTCTTTCTTTCTTTTTT----TTTTATCCAA
C.niviariensis      GCATGAATGCAGTATTTCTTTTATTTTTTTTCTTGCCAATTTTTCT--TTTTAAAAGCAA
C.glabrata          ATAAGAACTTTTTGTTATTCGGATGGGGGTGCGAGATGAGATGGTT--CATTTGGCTTTA
                      *                          * *         *   *    *        *

C.bracarensis       ATTACACCCATATTTTCAGGTACTTTATA-TAAGTTG-TTAATTATATATAT-ATATTTG
N.delphensis        TTTACACCCTTGTTTTAATGAATATATTAATAATCTGCTTAACAATTTATGCCATATATA
C.niviariensis      TTTACACCCATGTTTCTAATAACA--ACAATTAATAATCAGAAGATGTCTAC---TTGAA
C.glabrata          AA-GCACCCATTTCTTGCACACCCCCGAATGTTTTACGTATGAGCATA-ATAAAATGCA
                        ***** * * *             * *                *        *

C.bracarensis       GGCCTTTGCTATACAAGTAAGTTTCCTG--AGGTTAATAAGATGCTTGATTTACTGTTAC
N.delphensis        AGTATC-ATTTTGACAGTATAATCAAAG--AAGATCAGGAGAAA--TAGTTCTTGGCTGC
C.niviariensis      TGCAAAGGATATAAAAGACAATCATATGCTAAATGTACATAATTTTTGTTATATAGTTTT
C.glabrata          CTTGCCTTGTAATATAACTTGAACAAGGTTGAATATATAAAATGTACATTTGGATGCTGC
                             *     *           *        *    *       *     * *

C.bracarensis       CTATTAGAACTTGTATATATTATCATTTTGT-TACGTACTCACATTAATATTTAGGTAGT
N.delphensis        ATCTT----CTTATATCTATTCTTCTTCAACGTATATAAAAAATTAAAGATATAAGGTAA
C.niviariensis      ATTTTAGGAATTTTTTATTTATTTTTTTCTTTAATGTTTT---GTACCCACTTACAGTGT
C.glabrata          -TGGAGTGGGCCCTATATAGTTTTCTTTCAGTCCCATTAGCGTTTA---GTTTAGTTAGT
                     *           * * *    *  **         *       *       **

C.bracarensis       TATTTAGCGCCATATACCTTATAAGAGTCATATATAACTACTAGGAAAGGATAAAG
N.delphensis        CGTTTAGACACTTA-ATACTGAAGTATTAATAGTAAATTAAAAG------------
C.niviariensis      AAACTAGCGCTAAGTAT-TTAAAATAGTAATAACGAAATA----------------
C.glabrata          CAAAGAGGTTTATAGAATTTAGTGTGG-AATAACTAGCTCAAATA-----------
                         **        *   *         ***   *  *
```

Supplementary file S15: Multiple alignment of the promoters of the *DOM34b* genes in four *Nakaseomyces* species. The alignment was performed using Clustalw (www.genome.jp/tools-bin/clustalw) with default parameters. The stars indicate perfectly conserved positions. The alignment of a Aft site in the four species is undelined by a black box. The presence of a perfect ACACCC motif in the *C. glabrata* sequence, close to the optimal alignment has also been underlined.

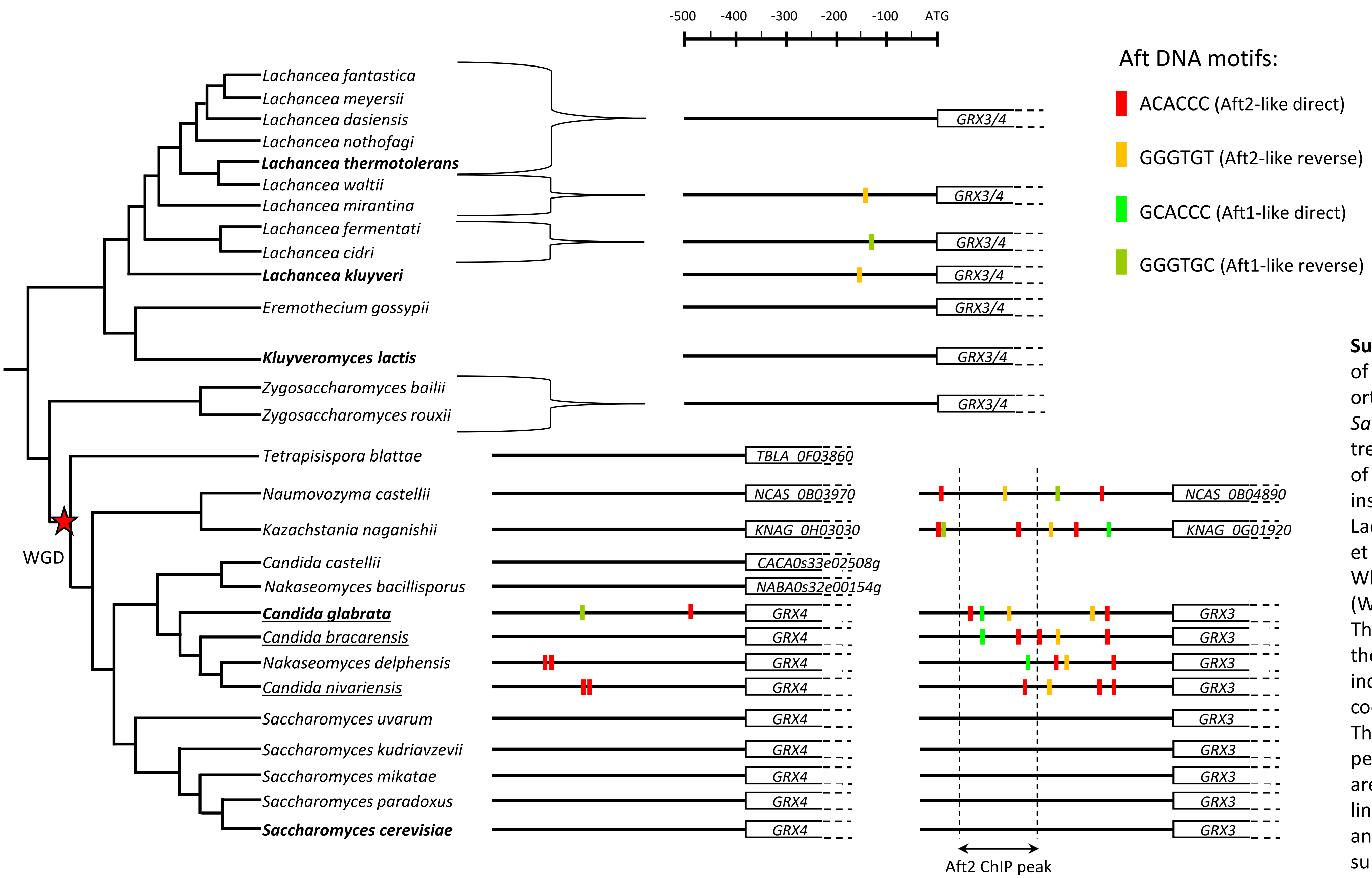

Supplementary file S16: Analyses of the promoters of the *GRX3/4* orthogroup in 28 *Saccharomycetaceae* species. The tree is a schematic representation of the phylogeny of the species inspired from Vakirlis et al. for the Lachancea species and from Shen et al. for all the other species. The Whole Genome Duplication (WGD) is indicated by a red star. The positions of the Aft sites in the promoters of the genes is indicated by colored boxes (color code is on the upper right part). The boundaries of the Aft2 ChIP peak in the *C. glabrata GRX3* gene are indicated by dashed vertical lines. The sequences used for this analysis can be found in supplementary file S7.

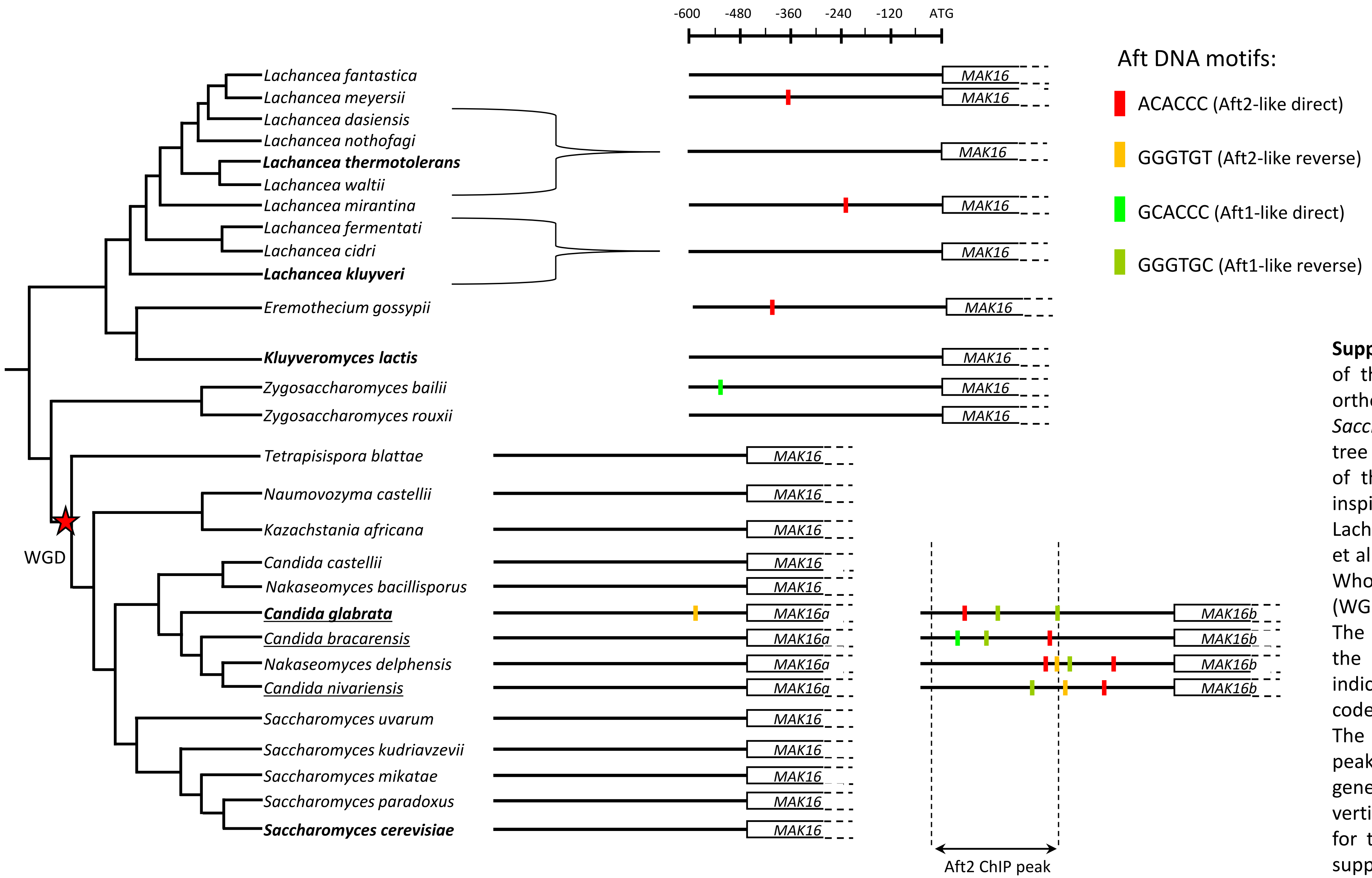

Supplementary file S17: Analyses of the promoters of the *MAK16* orthogroup in 28 *Saccharomycetaceae* species. The tree is a schematic representation of the phylogeny of the species inspired from Vakirlis et al. for the Lachancea species and from Shen et al. for all the other species. The Whole Genome Duplication (WGD) is indicated by a red star. The positions of the Aft sites in the promoters of the genes is indicated by colored boxes (color code is on the upper right part). The boundaries of the Aft2 ChIP peak in the *C. glabrata MAK16b* gene are indicated by dashed vertical lines. The sequences used for this analysis can be found in supplementary file S8.
